# Supplementary material for: Translational Potential of Metabolomics on Animal Models of Inflammatory Bowel Disease—A Systematic Critical Review
Source: Int J Mol Sci. 2020 May 29;21(11):3856. doi: 10.3390/ijms21113856 (PMC7312423; doi:10.3390/ijms21113856)
Supplement: Supplementary file 1 [file ijms-21-03856-s001.zip › Supplementary Table S1,S2_resubmission_proofread.docx]

**Supplementary Table S1: Descriptive data from included human studies**

| Reference | 1st author Year Country | Groups | Sample size | Sex  (% female) | Age range,  (mean ± SD if available)  [median] | Ethnic origin  in all groups | Disease activity | Disease activity index(es) used | Treatment  Stated | Controls are  relatives | Sample type | Measurement  of specific  metabolites | Analytical platform  Targeted or untargeted |
| --- | --- | --- | --- | --- | --- | --- | --- | --- | --- | --- | --- | --- | --- |
| [1] | Ahmed 2016 UK | CDa  CDi  UCa  UCi  Ctrl | 62  55  48  52  109 | 52  30  48  40  63 | 18-80 (39)  18-80 (36.5)  18-80 (38)  18-80 (34.5  18-80 (33.3) | Caucasian,  British Asian, Asian, Others | Active  Inactive  Active  Inactive | HBI  SCCAI | Yes | Yes | Feces | VOMs | GC-MS  Untargeted |
| [2] | Alonso 2016 Spain | CD-d  CD-v  UC-d  UC-v  Ctrl-d  Ctrl-v | 203  200  213  200  100  200 | ~50% | 18-n/r | Caucasian | Active, inactive | HBI Lichtiger | Yes | No | Urine | No | ^1^H NMR  Untargeted |
| [3] | Balasubramanian 2009 India | CD  UC  Ctrl | 26  31  26 | n/r | n/r | n/r | Active, remission | CD: CDAI UC: Truelove and Witts' criteria | n/r | No | Colonic mucosa | No | Proton MRS  Untargeted |
| [4] | Bjerrum 2015 Denmark | CDa  CDi  UCa  UCi  Ctrl | 13  31  19  29  21 | 54  58  42  52  38 | 18-80 (44)  18-80 (36)  18-80 (48)  18-80 (48)  18-80 (40) | n/r | Active, inactive | HBI Mayo | Yes | No | Feces | No | ^1^H NMR  Untargeted |
| [5] | Bodelier 2015 Netherlands | CD  Ctrl | 191  110 | 63  54 | (43.6 ± 14.8)  n/r | n/r | Active, remission | HBI | No | No | Exhaled breath | VOCs | GC-tof-MS  Untargeted |
| [6] | Cracowski  2002 France | CD  Ctrl | 23  23 | 70  70 | 18-76 (41)  18-76 (45) | n/r | Active, remission | CDAI | Yes | No | Urine | Isoprostaglandin F2α type III | GC-MS  Targeted |
| [7] | Dawiskiba 2014 Poland | CD  UC  Ctrl | 19  24  17 | 42  38  53 | 18-70 (35.8)  18-70 (40.7)  18-70 (44.7) | n/r | Active, remission | HBI  SCCAI | No | No | Serum  Urine | No | ^1^H NMR  Untargeted |
| [8] | De Preter 2013 Belgium | CD  Ctrl | 45  40 | 64  70 | 31-52 [42]  21-36 [27] | n/r | Moderate, mild, inactive | HBI | Yes | No | Feces | VOCs | GC-MS  Untargeted |
| [9] | De Preter 2015 Belgium | CD  UC  Ctrl | 83  68  40 | 59  37  70 | 18-78 [41]  18-78 [48]  18-78 [n/r] | n/r | Moderate, mild,  inactive | HBI  UCDAI | Yes | No | Feces | VOCs | GC-tof-MS  Untargeted |
| [10] | Fathi 2014 Iran | CD  Ctrl | 26  29 | n/r | (34 ± 11)  (35 ± 12) | n/r | Active | None | No | No | Serum | No | ^1^H NMR  Untargeted |

(Supplementary Table S1 – continued (I))

| Reference | 1st author Year Country | Groups | Sample size | Sex  (% female) | Age range,  (mean ± SD if available)  [median] | Ethnic origin  in all groups | Disease activity | Disease activity index(es) used | Treatment  Stated | Controls are  relatives | Sample type | Measurement  of specific  metabolites | Analytical platform  Targeted or untargeted |
| --- | --- | --- | --- | --- | --- | --- | --- | --- | --- | --- | --- | --- | --- |
| [11] | Hicks 2015 UK | CD  UC  Ctrl | 18  20  18 | 61  50  55 | 21-82[45.5]  21-82[49.5]  21-82[44.5] | Caucasian | Active, inactive | HBI  SCCAI | Yes | No | Exhaled breath | VOCs | SIFT-MS  Targeted |
| [12] | Iwamoto 2013 Japan | CD  CD-PR  UC  Ctrl | 17  4  10  26 | 18  0  30  23 | (32.3 ± 2.5)  (44.5 ± 9.8)  (45.2 ± 5.5)  (34.9 ± 1.8) | n/r | Active, inactive | CD: CDAI UC: Mayo-score | Yes | No | Serum | Sterols and bile acids | LC-ESI-MS/MS  Targeted |
| [13] | Jacobs 2016 USA | CD  UC  Ctrl | 26  10  54 | 30  30  51 | n/r  n/r  n/r | Caucasian,  Jewish,  Mixed | Remission | HBI  Partial Mayo score | Yes | Yes | Feces | No | UPLC/ToFMS Untargeted |
| [14] | Jansson 2009 Sweden | CCD  ICD  Ctrl | 8  6  20 | 25  50  50 | 5-70 (48)  5-70 (49.9)  5-70 (36.4) | n/r | Remission | HBI | No | Yes (MZ or DZ twins) | Feces | No | FT-ICR-MS  Untargeted |
| [15] | Johnson 2006 USA | CD  Ctrl | 28  72 | 54  26 | (38.8 ± 1.7)  (60.9 ± 7.5) | n/r | n/r | None | No | No | Urine | Prostaglandin E_2_ | LC-MS  Targeted |
| [16] | Kohashi 2014 Japan | CD  UC-t  UC-v  Ctrl-t  Ctrl-v | 39  60  60  60  60 | 33  65  63  62  62 | 13-77 (35.4 ± 12.0)  13-77 (42.8 ± 14.1)  13-77 (41.9 ± 15.0)  13-77 (45.4 ± 11.1)  13-77 (45.4 ± 10.8) | n/r | Active, remission | CDAI  Rachmilewitz (CAI) | Yes | No | Serum | No | GC-MS  Untargeted |
| [17] | Le Gall 2011 UK | UC  Ctrl | 13  22 | 62  68 | 26-63 (49.0 ± 13.5)  26-63 (46.55 ± 8.8) | n/r | Moderate, mild, inactive | Mayo | Yes | No | Feces | No | ^1^H NMR  Untargeted |
| [18] | Machiels 2014 Belgium | UC  Ctrl | 127  87 | 42  55 | 30-53 [43]  30-53 [41,5] | n/r | Severe, moderate, quiescent | Mayo | Yes | No | Feces | SCFAs | GC-MS  Targeted |
| [19] | Marchesi 2007 Ireland | CD  UC  Ctrl | 10  10  13 | 60  70  54 | (30 ± 14)  (49 ± 12)  (33 ± 9) | n/r | n/r | None | Yes | No | Feces | No | ^1^H NMR  Untargeted |

(Supplementary Table S1 – continued (II))

| Reference | 1st author Year Country | Groups | Sample size | Sex  (% female) | Age range,  (mean ± SD if available)  [median] | Ethnic origin  in all groups | Disease activity | Disease activity index(es) used | Treatment  Stated | Controls are  relatives | Sample type | Measurement  of specific  metabolites | Analytical platform  Targeted or untargeted |
| --- | --- | --- | --- | --- | --- | --- | --- | --- | --- | --- | --- | --- | --- |
| [20] | Martin 2016 Switzerland | CD  UC  Ctrl | 15  6  27 | 47  67  41 | 10-18 (14.9 ± 1.3)  10-18 (15 ± 1.2)  10-18 (12.9 ± 1.9) | n/r | CD: Moderate, mild, remission UC: Mild, remission | PCDAI  PUCAI | Yes | No | Urine | No | ^1^H NMR  Untargeted |
| [21] | Ooi 2011 Japan | CD  UC-s  UC-ti  Ctrl | 21  13  22  17 | 29  46  46  29 | 14-85 [34.9]  14-85 [39]  14-85 [43.9]  14-85 [38] | n/r | Active, remission | CD: IOIBD score UC: Rachmilewitz (CAI) | Yes | No | Serum  Rectum | Amino acids and TCA cycle-related low molecular weight metabolites | GC-MS  Untargeted |
| [22] | Patel  2014 USA | IBD Ctrl | 62  55 | 47  36 | 5-21 (15.7 ± 3.3)  5-21 (12.1 ± 3.0) | Caucasian, Black, Other | Active, remission | CD: PCDAI  UC: PUCAI | Yes | No | Exhaled breath | VOCs | SIFT-MS  Untargeted |
| [23] | Rieder 2016 USA | CD  UC  Ctrl | 24  11  53 | 54  64  68 | (45.9 ± 12.9)  (43.9 ± 15.9)  (42.6 ± 14.6) | Caucasian, African American, Asian, Hispanic | n/r | HBI Lichtiger | Yes | No | Exhaled breath | VOCs | SIFT-MS  Targeted |
| [24] | Schicho 2012 Canada | CD  UC  Ctrl | 20  20  40 | 10  35  23 | 20-85 (39.6)  20-85 (46,2)  20-85 (53,7) | n/r | Active | HBI  SCCAI | Yes | No | Urine  Serum  Plasma | No | ^1^H NMR  Targeted |
| [25] | Sewell 2012 UK | a. CD  a. Ctrl  b. CD  b. Ctrl  c. CD  c. Ctrl | 8  7  13  10  5  5 | 50  39  54  50  40  80 | 19-63 (36.2)  19-63 (44.1)  22-70 (36.5)  22-70 (33.5)  19-63 (33.1)  19-63 (40.0) | n/r | a. Remission b. Remission c. Active, remission | HBI | Yes | No | a. PBMC Macrophages b. PBMC Macrophages c. Ileal tissue | a. Sphingolipids b. Phospholipids c. Lipids | a. HPLC-MS  Untargeted b. ESI-MS  Targeted c. ESI-MS  Untargeted |
| [26] | Sharma 2010 India | CD  UC  Ctrl | 9  12  26 | n/r | (43 ± 12.4)  (37.5 ± 12.6)  (n/r) | n/r | Active | CD: CDAI UC: Truelove and Witts' criteria | n/r | No | Colonic mucosa | No | ^1^H NMR  Untargeted |
| [27] | Shiomi 2011 Japan | UC-ti  UC-s  Ctrl | 5  5  5 | 60  40  40 | 24-61 (44.2)  24-61 (47)  24-61 (42.4) | n/r | Moderate, mild | UCDAI | Yes | No | Colon  Serum | No | GC-MS  Untargeted |

(Supplementary Table S1 – continued (III))

| Reference | 1st author Year Country | Groups | Sample size | Sex  (% female) | Age range,  (mean ± SD if available)  [median] | Ethnic origin  in all groups | Disease activity | Disease activity index(es) used | Treatment  Stated | Controls are  relatives | Sample type | Measurement  of specific  metabolites | Analytical platform  Targeted or untargeted |
| --- | --- | --- | --- | --- | --- | --- | --- | --- | --- | --- | --- | --- | --- |
| [28] | Stephens 2013 Canada | CD  UC  Ctrl | 30  30  60 | 53  53  50 | 18-72 (37)  18-72 (39)  18-72 (41) | n/r | Severe, moderate, mild, remission | HBI  Mayo | Yes | No | Urine | No | NMR  Targeted |
| [29] | Thyssen 1996 USA | UC  UC res  Ctrl  Ctrl res | 11  4  8  3 | 46  25  50  33 | 21-85 (41)  21-85 (34)  21-85 (56)  21-85 (66) | n/r | Active | n/r | No | No | Colonic mucosa | PAFs, lyso-PAFs | GC-MS  Targeted |
| [30] | Williams 2009 UK | CD  UC  Ctrl | 86  60  60 | 45  50  50 | 16-66 [33]  16-66 [40]  16-66 [30] | Caucasian | Active, remission | HBI  SCCAI | Yes | No | Urine | No | ^1^H NMR  Untargeted |
| [31] | Williams 2010 UK | CD  Ctrl | 16  16 | 56  44 | 22-66 [42]  22-66 [33] | n/r | Remission | HBI | No | No | Urine | Hippurate | ^1^H NMR  Targeted |
| [32] | Yau 2014 Australia | CD  UC  Ctrl | 25  19  9 | 68  63  56 | (36 ± 14)  (42 ± 17)  (30 ± 9) | n/r | Severe, moderate, mild, remission | CDAI  Mayo | Yes | No | Plasma | a) No b) Picolinic acid, Quinolinic acid | a) LC-MS/MS  Untargeted b) GC-MS  Targeted |
| [33] | Zhang 2013 China | UC  Ctrl | 20  19 | 55  53 | 22-62 (42)  22-62 (37) | n/r | Active | Mayo | Yes | No | Serum | No | ^1^H NMR  Untargeted |

n/r = not reported; CD = Crohn’s disease; IBD = Inflammatory Bowel Disease; UC = Ulcerative colitis; CDa/UCa = patients with active disease; CDi/UCi = patients with inactive disease; UC-t = subject groups in training set; UC-v/CD-v/Ctrl-v = subject groups in validation set; CD-d/UC-d/Ctrl-d = subject groups in discovery set; UC-s = serum measurement group of UC patients; UC-ti = tissue measurement group of UC patients; UC/Ctrl res = tissue taken from surgical resections; HBI = Harvey-Bradshaw Index; PCDAI = Pediatric CD Activity Index; PUCAI = Pediatric UC Activity Index; SCCAI = Simple Clinical Colitis Activity Index; UCDAI = UC Disease Activity Index; CDAI = CD Activity Index; CAI = Colitis Activity Index; IOIBD = International Organization for the Study of IBD; MZ = monozygotic; DZ = dizygotic; RT = room temperature; VOMs = volatile organic metabolites; VOC = volatile organic compounds; SCFAs = short-chain fatty acids; PAFs = platelet activating factors; UPLC/HPLC = ultra/high performance liquid chromatography; ToF = time-of-flight; MS = mass spectrometry; GC = gas chromatography; NMR = nuclear magnetic resonance; SIFT = selected-ion flow-tube; LC = liquid chromatography; ESI = electrospray ionization; MRS = magnetic resonance spectroscopy; FT-ICR = Fourier-transform ion cyclotron resonance.

**Supplementary Table S2: Descriptive data from included animal studies**

| Reference | 1st author Year Country | Disease modeled | Sample size (cases/controls) | Species Strain | Sex | Age  (weeks) | Housing  (type) | Housing (# of animals) | Model*  (Acute/  Chronic) | Sample type | Time of sampling | Measurement  of specific metabolites | Analytical platform  Targeted or untargeted |
| --- | --- | --- | --- | --- | --- | --- | --- | --- | --- | --- | --- | --- | --- |
| [34] | Baur 2011 Germany | CD-like ileitis | 48/48 | Mouse C57BL/6 | M/F | 4-24 | Conventional | 1 | TNF^ΔARE/WT^  (Chronic) | Distal jejunum  Distal ileum  Proximal colon  Distal colon | n/r | a) No b) No c) Lipids | a) ^1^H NMR  Untargeted b) LC-MS  Targeted c) LC-MS  Targeted |
| [35] | Dong 2013 China | UC | 12/12 | Mouse BALB/c | M | 10-11 | SPF | n/r | DSS (3%)  (Acute) | Urine  Plasma  Colon  Liver  Spleen | 19:00-22:00 | No | ^1^H NMR  Untargeted |
| [36] | Gu 2015 USA | Colitis | 4/4 | Mouse C57BL/6J | M | 8-10 | n/r | n/r | DSS (3%)  (Acute) | Serum Colon | n/r | No | GC-MS  Untargeted |
| [37] | Hong 2010 Korea | Colitis | 5/5 | Mouse ICR. | M | 7 | Conventional | 1 | DSS (2.5%)  (Acute) | Feces | n/r | No | ^1^H NMR  Untargeted |
| [38] | Hou 2016 China | CD | 9/9 | Rat Sprague-Dawley | M | n/r | n/r | n/r | TNBS  (90 mg/kg BW)  (Acute) | Serum  Urine  Feces | 9.00-  17.00 | Organic acids, amines | UPLC-MS/MS  Targeted |
| [39] | Jacobs 2017 USA | Colitis | 12-18/12-18 | Mouse C57BL/6 | M/F | 3,5,9 | SPF | n/r  (but co-housing) | Hz floxed T-synthase, intestine-specific deletion; SPF  (Chronic) | Distal colonic mucosa  Cecum mucosa  Luminal content | n/r | No | UPLC/ToF-MS  Untargeted |
| [40] | Köhnke 2013 Germany | Colitis | 6/3 | Mouse C57BL/6 | F | 7 | n/r | n/r | DSS (3%)  (Acute) | Colon | n/r | Lipids | HPLC-MS/MS  Targeted |
| [41] | Kominsky 2011 USA | Colitis | 6/6 | Mouse C57BL/6 | n/r | 13 | n/r | n/r | DSS (3%)  (Acute) | Colon | n/r | No | NMR (^1^H, ^1^C, ^1^P)  Untargeted |
| [42] | Lin 2009 New Zealand | CD | Discovery: 15/8 Validation: 10/6 | Mouse C57BL/6 | M | 6-12 | Conventional | 1 | IL10^-/-^  (Chronic) | Urine | 9:00-  16:00 | No | GC-MS  Untargeted |

(Supplementary Table S2 – continued (I))

| Reference | 1st author Year Country | Disease modeled | Sample size (cases/controls) | Species Strain | Sex | Age  (weeks) | Housing  (type) | Housing (# of animals) | Model | Sample type | Time of sampling | Measurement  of specific metabolites | Analytical platform  Targeted or untargeted |
| --- | --- | --- | --- | --- | --- | --- | --- | --- | --- | --- | --- | --- | --- |
| [43] | Lin 2010 New Zealand | CD | 30/30 | Mouse C57BL/6 | M | 6-10 | Conventional, IVC SPF | 1 | IL10^-/-^  (Chronic) | a) Urine  b) Plasma | 10:00-12:00 | No | a) GC-MS  Untargeted  b) LC-MS  Targeted |
| [44] | Liu 2016 China | Colitis | 8/8 | Mouse C57BL/6 | M | 15-16 | n/r | n/r | DSS (2%)  (Chronic) | Colon  Liver  Spleen | n/r | No | LC-qTOF-MS  Untargeted |
| [45] | Lu 2012 USA | IBD | 10/10 | Mouse 129/SvEv Rag2^-/-^ | n/r | 17-19  /27-29 | Static microisolator cages | n/r | H. hepaticus  (Chronic) | Serum | n/r | No | UPLC-ESI-TOF-MS  Untargeted |
| [46] | Martin 2009 Switzerland | Colitis | 20/20 | Mouse 129S6/SvEvTac (Ctrl)/129(B6)-Il10^tm1Cgn^/J (Cases) | n/r | 1-24 | n/r | n/r | IL10^-/-^  (Chronic) | Plasma | n/r | No | ^1^H NMR  Untargeted |
| [47] | Martin 2015 Switzerland | Colitis | 10/10 | Mouse C57BL/6 | F | 12-16 | n/r | n/r | T cell transfer to Rag2^-/-^  (Chronic) | Urine  Plasma  Liver  Stool | n/r | No | ^1^H NMR  Untargeted |
| [48] | Murdoch 2008 Canada | IBD | 4/3 | Mouse 129/SvEv | M | 5-20 | Conventional | 1 | IL10^-/-^  (Chronic) | Urine | 22h period | No? | NMR  Untargeted |
| [49] | Otter 2011 New Zealand | CD | n/r | Mouse C57BL/6 | M | 6-11/7-12/6-10 | Conventional, SPF | 1 | IL10^-/-^  (Chronic) | Urine | n/r | No | Short-column LC-MS, LC-MS/MS, UHPLC-MS  Untargeted |
| [50] | Qi 2014 USA | Colitis | 4/4 | Mouse C57Bl6/N | M | 7-9 | n/r | n/r | DSS (3%) TNBS (2 mg)  (Acute) | Serum Colon | n/r | Lipids | UPLC-ESI-qTOF-MS  Targeted |
| [51] | Qu 2017 China | Colitis | 16-20/16-20 | Mouse BalB/C | M | 9-10 | n/r | n/r | DSS (3%)  (Acute) | Plasma | n/r | a) No b) Tryptophan catabolism | UPLC-MS  a) Untargeted b) Targeted |
| [52] | Robinson 2016 Australia | Colitis | 6/4 | Mouse *Winnie* and C57BL/6 | n/r | 12 | n/r | n/r | *Winnie*  (Chronic) | Feces | n/r | No | GC-MS  Untargeted |

(Supplementary Table S2 – continued (II))

| Reference | 1st author Year Country | Disease modeled | Sample size (cases/controls) | Species Strain | Sex | Age  (weeks) | Housing  (type) | Housing (# of animals) | Model | Sample type | Time of sampling | Measurement  of specific metabolites | Analytical platform  Targeted or untargeted |
| --- | --- | --- | --- | --- | --- | --- | --- | --- | --- | --- | --- | --- | --- |
| [53] | Schicho 2010 Canada | UC | 5-11/5-11 | Mouse CD1 | M | 4-6 | Conventional | n/r | DSS (4%)  (Acute) | Serum | n/r | No | ^1^H NMR  Targeted |
| [27] | Shiomi 2011 Japan | Colitis | 6/6 | Mouse C57BL/6J | F | 8-10 | SPF | n/r | DSS (3%)  (Acute) | Serum  Colon | n/r | No | GC-MS  Untargeted |
| [54] | Vassilyadi 2016 Canada | Colitis | 6-7/6-7 | Piglet Yorkshire × Landrace | n/r | 3 | N/A | n/r | DSS (1 g/kg/day)  (Acute) | Liver  RBCs  Distal colon  Spiral colon  Small intestine  Longissimus dorsi  Masseter | n/r | Gluthathione metabolites | UHPLC/MS-MS  Targeted |
| [55] | Wang 2016 China | Colitis | 5/5 | Mouse C57BL/6J | M | 8-10 | n/r | n/r | DSS (3%)  (Acute) | Serum  Colon | n/r | a) Lipids b) Fatty acids | a) UPLC-MS b) GC-MS  Targeted |
| [56] | Willenberg 2015 Germany | Colitis | 5/4 | Rat Fischer 344 | M | 8-14 | Conventional | n/r | DSS (4%)  (Acute +  Chronic) | Plasma  Colon | n/r | Eicosanoids, oxylipins | LC-MS  Targeted |
| [57] | Zhang 2012 China | Colitis | Urine: 7/10 Plasma: 9/9 | Rat Sprague-Dawley | M | n/r | Conventional + Metabolic cages | n/r | TNBS (30 mg/rat)  (Acute) | Urine  Plasma | n/r | No | UPLC-ESI-qTOF-MS  Untargeted |
| [58] | Zhang 2013 USA | IBD | 20/20 | Mouse C57BL/6J | M/F | 25 (M),  38 (F) | Microisolator cage | 3-5 | IL10^-/-^  (Chronic) | Plasma | n/r | Eicosanoids | LC/MS-MS  Targeted |

*TNBS and DSS models have been allocated as ‘acute’ or ‘chronic’ according to the criteria in Wirtz et al. [59] for chemical induction of acute and chronic colitis models. Models were allocated as ‘chronic’ if they were genetically modified or adaptive. CD = Crohn’s disease; UC = ulcerative colitis; IBD = inflammatory bowel disease; SPF = specific pathogen free; TNBS = trinitrobenzenesulfonic acid; DSS = dextran sulfate sodium; UPLC/HPLC = ultra/high performance liquid chromatography; qTOF = quadrupole time-of-flight; ESI = electrospray ionization; MS = mass spectrometry; GC = gas chromatography; NMR = nuclear magnetic resonance; LC = liquid chromatography.

**References**

1. Ahmed, I.; Greenwood, R.; Costello, B.; Ratcliffe, N.; Probert, C.S. Investigation of faecal volatile organic metabolites as novel diagnostic biomarkers in inflammatory bowel disease. *Alimentary Pharmacology and Therapeutics* **2016**, *43*, 596-611.

2. Alonso, A.; Julia, A.; Vinaixa, M.; Domenech, E.; Fernandez-Nebro, A.; Canete, J.D.; Ferrandiz, C.; Tornero, J.; Gisbert, J.P.; Nos, P., et al. Urine metabolome profiling of immune-mediated inflammatory diseases. *BMC medicine* **2016**, *14*, 133, doi:10.1186/s12916-016-0681-8.

3. Balasubramanian, K.; Kumar, S.; Singh, R.R.; Sharma, U.; Ahuja, V.; Makharia, G.K.; Jagannathan, N.R. Metabolism of the colonic mucosa in patients with inflammatory bowel diseases: an in vitro proton magnetic resonance spectroscopy study. *Magnetic Resonance Imaging* **2009**, *27*, 79-86.

4. Bjerrum, J.T.; Wang, Y.; Hao, F.; Coskun, M.; Ludwig, C.; Gunther, U.; Nielsen, O.H. Metabonomics of human fecal extracts characterize ulcerative colitis, Crohn's disease and healthy individuals. *Metabolomics : Official journal of the Metabolomic Society* **2015**, *11*, 122-133, doi:10.1007/s11306-014-0677-3.

5. Bodelier, A.G.L.; Smolinska, A.; Baranska, A.; Dallinga, J.W.; Mujagic, Z.; Vanhees, K.; Van Den Heuvel, T.; Masclee, A.A.M.; Jonkers, D.; Pierik, M.J., et al. Volatile organic compounds in exhaled air as novel marker for disease activity in Crohn's disease: A metabolomic approach. *Inflammatory bowel diseases* **2015**, *21*, 1776-1785.

6. Cracowski, J.L.; Bonaz, B.; Bessard, G.; Bessard, J.; Anglade, C.; Fournet, J. Increased urinary F2-isoprostanes in patients with Crohn's disease. *American Journal of Gastroenterology* **2002**, *97*, 99-103.

7. Dawiskiba, T.; Deja, S.; Mulak, A.; Zabek, A.; Jawien, E.; Pawelka, D.; Banasik, M.; Mastalerz-Migas, A.; Balcerzak, W.; Kaliszewski, K., et al. Serum and urine metabolomic fingerprinting in diagnostics of inflammatory bowel diseases. *World journal of gastroenterology : WJG* **2014**, *20*, 163-174, doi:10.3748/wjg.v20.i1.163.

8. De Preter, V.; Joossens, M.; Ballet, V.; Shkedy, Z.; Rutgeerts, P.; Vermeire, S.; Verbeke Phd, K. Metabolic profiling of the impact of oligofructose-enriched inulin in Crohn's disease patients: a double-blinded randomized controlled trial. *Clinical and translational gastroenterology* **2013**, *4*, e30, doi:10.1038/ctg.2012.24.

9. De Preter, V.; Machiels, K.; Joossens, M.; Arijs, I.; Matthys, C.; Vermeire, S.; Rutgeerts, P.; Verbeke, K. Faecal metabolite profiling identifies medium-chain fatty acids as discriminating compounds in IBD. *Gut* **2015**, *64*, 447-458.

10. Fathi, F.; Majari-Kasmaee, L.; Mani-Varnosfaderani, A.; Kyani, A.; Rostami-Nejad, M.; Sohrabzadeh, K.; Naderi, N.; Zali, M.R.; Rezaei-Tavirani, M.; Tafazzoli, M., et al. 1H NMR based metabolic profiling in Crohn's disease by random forest methodology. *Magnetic resonance in chemistry : MRC* **2014**, *52*, 370-376.

11. Hicks, L.C.; Huang, J.; Kumar, S.; Powles, S.T.; Orchard, T.R.; Hanna, G.B.; Williams, H.R. Analysis of Exhaled Breath Volatile Organic Compounds in Inflammatory Bowel Disease: A Pilot Study. *Journal of Crohn's & colitis* **2015**, *9*, 731-737.

12. Iwamoto, J.; Saito, Y.; Honda, A.; Miyazaki, T.; Ikegami, T.; Matsuzaki, Y. Bile acid malabsorption deactivates pregnane x receptor in patients with Crohn's Disease. *Inflammatory bowel diseases* **2013**, *19*, 1278-1284.

13. Jacobs, J.P.; Goudarzi, M.; Singh, N.; Tong, M.; McHardy, I.H.; Ruegger, P.; Asadourian, M.; Moon, B.H.; Ayson, A.; Borneman, J., et al. A Disease-Associated Microbial and Metabolomics State in Relatives of Pediatric Inflammatory Bowel Disease Patients. *Cellular and molecular gastroenterology and hepatology* **2016**, *2*, 750-766.

14. Jansson, J.; Willing, B.; Lucio, M.; Fekete, A.; Dicksved, J.; Halfvarson, J.; Tysk, C.; Schmitt-Kopplin, P. Metabolomics reveals metabolic biomarkers of Crohn's disease. *PloS one* **2009**, *4*, e6386, doi:10.1371/journal.pone.0006386.

15. Johnson, J.C.; Schmidt, C.R.; Shrubsole, M.J.; Billheimer, D.D.; Joshi, P.R.; Morrow, J.D.; Heslin, M.J.; Washington, M.K.; Ness, R.M.; Zheng, W., et al. Urine PGE-M: A Metabolite of Prostaglandin E2 as a Potential Biomarker of Advanced Colorectal Neoplasia. *Clinical Gastroenterology and Hepatology* **2006**, *4*, 1358-1365.

16. Kohashi, M.; Nishiumi, S.; Ooi, M.; Yoshie, T.; Matsubara, A.; Suzuki, M.; Hoshi, N.; Kamikozuru, K.; Yokoyama, Y.; Fukunaga, K., et al. A novel gas chromatography mass spectrometry-based serum diagnostic and assessment approach to ulcerative colitis. *Journal of Crohn's and Colitis* **2014**, *8*, 1010-1021.

17. Le Gall, G.; Noor, S.O.; Ridgway, K.; Scovell, L.; Jamieson, C.; Johnson, I.T.; Colquhoun, I.J.; Kemsley, E.K.; Narbad, A. Metabolomics of fecal extracts detects altered metabolic activity of gut microbiota in ulcerative colitis and irritable bowel syndrome. *Journal of proteome research* **2011**, *10*, 4208-4218.

18. Machiels, K.; Joossens, M.; Sabino, J.; De Preter, V.; Arijs, I.; Eeckhaut, V.; Ballet, V.; Claes, K.; Van Immerseel, F.; Verbeke, K., et al. A decrease of the butyrate-producing species roseburia hominis and faecalibacterium prausnitzii defines dysbiosis in patients with ulcerative colitis. *Gut* **2014**, *63*, 1275-1283.

19. Marchesi, J.R.; Holmes, E.; Khan, F.; Kochhar, S.; Scanlan, P.; Shanahan, F.; Wilson, I.D.; Wang, Y. Rapid and noninvasive metabonomic characterization of inflammatory bowel disease. *Journal of proteome research* **2007**, *6*, 546-551.

20. Martin, F.P.; Ezri, J.; Cominetti, O.; Da Silva, L.; Kussmann, M.; Godin, J.P.; Nydegger, A. Urinary metabolic phenotyping reveals differences in the metabolic status of healthy and inflammatory bowel disease (IBD) children in relation to growth and disease activity. *International journal of molecular sciences* **2016**, *17*, no pagination.

21. Ooi, M.; Nishiumi, S.; Yoshie, T.; Shiomi, Y.; Kohashi, M.; Fukunaga, K.; Nakamura, S.; Matsumoto, T.; Hatano, N.; Shinohara, M., et al. GC/MS-based profiling of amino acids and TCA cycle-related molecules in ulcerative colitis. *Inflammation Research* **2011**, *60*, 831-840.

22. Patel, N.; Alkhouri, N.; Eng, K.; Cikach, F.; Mahajan, L.; Yan, C.; Grove, D.; Rome, E.S.; Lopez, R.; Dweik, R.A. Metabolomic analysis of breath volatile organic compounds reveals unique breathprints in children with inflammatory bowel disease: A pilot study. *Alimentary Pharmacology and Therapeutics* **2014**, *40*, 498-507.

23. Rieder, F.; Kurada, S.; Grove, D.; Cikach, F.; Lopez, R.; Patel, N.; Singh, A.; Alkhouri, N.; Shen, B.; Brzezinski, A., et al. A Distinct Colon-Derived Breath Metabolome is Associated with Inflammatory Bowel Disease, but not its Complications. *Clinical and translational gastroenterology* **2016**, *7*, e201, doi:10.1038/ctg.2016.57.

24. Schicho, R.; Shaykhutdinov, R.; Ngo, J.; Nazyrova, A.; Schneider, C.; Panaccione, R.; Kaplan, G.G.; Vogel, H.J.; Storr, M. Quantitative metabolomic profiling of serum, plasma, and urine by 1H NMR spectroscopy discriminates between patients with inflammatory bowel disease and healthy individuals. *Journal of proteome research* **2012**, *11*, 3344-3357.

25. Sewell, G.W.; Hannun, Y.A.; Han, X.; Koster, G.; Bielawski, J.; Goss, V.; Smith, P.J.; Rahman, F.Z.; Vega, R.; Bloom, S.L., et al. Lipidomic profiling in Crohn's disease: abnormalities in phosphatidylinositols, with preservation of ceramide, phosphatidylcholine and phosphatidylserine composition. *The international journal of biochemistry & cell biology* **2012**, *44*, 1839-1846, doi:10.1016/j.biocel.2012.06.016.

26. Sharma, U.; Singh, R.R.; Ahuja, V.; Makharia, G.K.; Jagannathan, N.R. Similarity in the metabolic profile in macroscopically involved and un-involved colonic mucosa in patients with inflammatory bowel disease: An in vitro proton (1H) MR spectroscopy study. *Magnetic Resonance Imaging* **2010**, *28*, 1022-1029.

27. Shiomi, Y.; Nishiumi, S.; Ooi, M.; Hatano, N.; Shinohara, M.; Yoshie, T.; Kondo, Y.; Furumatsu, K.; Shiomi, H.; Kutsumi, H., et al. GCMS-based metabolomic study in mice with colitis induced by dextran sulfate sodium. *Inflammatory bowel diseases* **2011**, *17*, 2261-2274.

28. Stephens, N.S.; Siffledeen, J.; Su, X.; Murdoch, T.B.; Fedorak, R.N.; Slupsky, C.M. Urinary NMR metabolomic profiles discriminate inflammatory bowel disease from healthy. *Journal of Crohn's and Colitis* **2013**, *7*, e42-e48.

29. Thyssen, E.; Turk, J.; Bohrer, A.; Stenson, W.F. Quantification of distinct molecular species of platelet activating factor in ulcerative colitis. *Lipids* **1996**, *31*, S255-S259.

30. Williams, H.R.T.; Cox, I.J.; Walker, D.G.; North, B.V.; Patel, V.M.; Marshall, S.E.; Jewell, D.P.; Ghosh, S.; Thomas, H.J.W.; Teare, J.P., et al. Characterization of inflammatory bowel disease with urinary metabolic profiling. *American Journal of Gastroenterology* **2009**, *104*, 1435-1444.

31. Williams, H.R.; Cox, I.J.; Walker, D.G.; Cobbold, J.F.; Taylor-Robinson, S.D.; Marshall, S.E.; Orchard, T. Differences in gut microbial metabolism are responsible for reduced hippurate synthesis in Crohn's disease. *Gastroenterology* **2010**, *138*, S579.

32. Yau, Y.Y.; Leong, R.W.L.; Shin, S.; Bustamante, S.; Pickford, R.; Hejazi, L.; Campbell, B.; Wasinger, V.C. Bimodal plasma metabolomics strategy identifies novel inflammatory metabolites in inflammatory bowel diseases. *Discovery medicine* **2014**, *18*, 113-124.

33. Zhang, Y.; Lin, L.; Xu, Y.; Lin, Y.; Jin, Y.; Zheng, C. 1H NMR-based spectroscopy detects metabolic alterations in serum of patients with early-stage ulcerative colitis. *Biochemical and biophysical research communications* **2013**, *433*, 547-551.

34. Baur, P.; Martin, F.P.; Gruber, L.; Bosco, N.; Brahmbhatt, V.; Collino, S.; Guy, P.; Montoliu, I.; Rozman, J.; Klingenspor, M., et al. Metabolic phenotyping of the Crohn's disease-like IBD etiopathology in the TNFDELTAARE/WT mouse model. *Journal of proteome research* **2011**, *10*, 5523-5535.

35. Dong, F.; Zhang, L.; Hao, F.; Tang, H.; Wang, Y. Systemic responses of mice to dextran sulfate sodium-induced acute ulcerative colitis using 1H NMR spectroscopy. *Journal of proteome research* **2013**, *12*, 2958-2966.

36. Gu, X.; Song, Y.; Chai, Y.; Lu, F.; Gonzalez, F.J.; Fan, G.; Qi, Y. GC-MS metabolomics on PPARalpha-dependent exacerbation of colitis. *Molecular bioSystems* **2015**, *11*, 1329-1337.

37. Hong, Y.S.; Ahn, Y.T.; Park, J.C.; Lee, J.H.; Lee, H.; Huh, C.S.; Kim, D.H.; Ryu, D.H.; Hwang, G.S. 1H NMR-based metabonomic assessment of probiotic effects in a colitis mouse model. *Archives of pharmacal research* **2010**, *33*, 1091-1101.

38. Hou, W.; Zhong, D.; Zhang, P.; Li, Y.; Lin, M.; Liu, G.; Yao, M.; Liao, Q.; Xie, Z. A strategy for the targeted metabolomics analysis of 11 gut microbiota-host co-metabolites in rat serum, urine and feces by ultra high performance liquid chromatography-tandem mass spectrometry. *Journal of Chromatography A* **2016**, *1429*, 207-217.

39. Jacobs, J.P.; Lin, L.; Goudarzi, M.; Ruegger, P.; McGovern, D.P.B.; Fornace, A.J.; Borneman, J.; Xia, L.; Braun, J. Microbial, metabolomic, and immunologic dynamics in a relapsing genetic mouse model of colitis induced by T-synthase deficiency. *Gut microbes* **2017**, *8*, 1-16.

40. Kohnke, T.; Gomolka, B.; Bilal, S.; Zhou, X.; Sun, Y.; Rothe, M.; Baumgart, D.C.; Weylandt, K.H. Acetylsalicylic Acid reduces the severity of dextran sodium sulfate-induced colitis and increases the formation of anti-inflammatory lipid mediators. *BioMed research international* **2013**, *2013*, 748160, doi:10.1155/2013/748160.

41. Kominsky, D.J.; Keely, S.; MacManus, C.F.; Glover, L.E.; Scully, M.; Collins, C.B.; Bowers, B.E.; Campbell, E.L.; Colgan, S.P. An endogenously anti-inflammatory role for methylation in mucosal inflammation identified through metabolite profiling. *J Immunol* **2011**, *186*, 6505-6514, doi:10.4049/jimmunol.1002805.

42. Lin, H.M.; Edmunds, S.J.; Helsby, N.A.; Ferguson, L.R.; Rowan, D.D. Nontargeted urinary metabolite profiling of a mouse model of crohn's disease. *Journal of proteome research* **2009**, *8*, 2045-2057.

43. Lin, H.M.; Barnett, M.P.G.; Roy, N.C.; Joyce, N.I.; Zhu, S.; Armstrong, K.; Helsby, N.A.; Ferguson, L.R.; Rowan, D.D. Metabolomic analysis identifies inflammatory and noninflammatory metabolic effects of genetic modification in a mouse model of Crohn?s disease. *Journal of proteome research* **2010**, *9*, 1965-1975.

44. Liu, J.; Xiao, H.T.; Wang, H.S.; Mu, H.X.; Zhao, L.; Du, J.; Yang, D.; Wang, D.; Bian, Z.X.; Lin, S.H. Halofuginone reduces the inflammatory responses of DSS-induced colitis through metabolic reprogramming. *Mol Biosyst* **2016**, *12*, 2296-2303, doi:10.1039/c6mb00154h.

45. Lu, K.; Knutson, C.G.; Wishnok, J.S.; Fox, J.G.; Tannenbaum, S.R. Serum metabolomics in a helicobacter hepaticus mouse model of inflammatory bowel disease reveal important changes in the microbiome, serum peptides, and intermediary metabolism. *Journal of proteome research* **2012**, *11*, 4916-4926.

46. Martin, F.P.J.; Rezzi, S.; Montoliu, I.; Philippe, D.; Tornier, L.; Messlik, A.; Holzlwimmer, G.; Baur, P.; Quintanilla-Fend, L.; Loh, G., et al. Metabolic assessment of gradual development of moderate experimental colitis in IL-10 deficient mice. *Journal of proteome research* **2009**, *8*, 2376-2387.

47. Martin, F.P.J.; Lichti, P.; Bosco, N.; Brahmbhatt, V.; Oliveira, M.; Haller, D.; Benyacoub, J. Metabolic phenotyping of an adoptive transfer mouse model of experimental colitis and impact of dietary fish oil intake. *Journal of proteome research* **2015**, *14*, 1911-1919.

48. Murdoch, T.B.; Fu, H.; MacFarlane, S.; Sydora, B.C.; Fedorak, R.N.; Slupsky, C.M. Urinary metabolic profiles of inflammatory bowel disease in interleukin-10 gene-deficient mice. *Analytical Chemistry* **2008**, *80*, 5524-5531.

49. Otter, D.; Cao, M.; Lin, H.M.; Fraser, K.; Edmunds, S.; Lane, G.; Rowan, D. Identification of urinary biomarkers of colon inflammation in IL10-/- mice using Short-Column LCMS metabolomics. *Journal of biomedicine & biotechnology* **2011**, *2011*, 974701, doi:10.1155/2011/974701.

50. Qi, Y.; Jiang, C.; Tanaka, N.; Krausz, K.W.; Brocker, C.N.; Fang, Z.Z.; Bredell, B.X.; Shah, Y.M.; Gonzalez, F.J. PPARalpha-dependent exacerbation of experimental colitis by the hypolipidemic drug fenofibrate. *American Journal of Physiology - Gastrointestinal and Liver Physiology* **2014**, *307*, G564-G573.

51. Qu, C.; Yuan, Z.W.; Yu, X.T.; Huang, Y.F.; Yang, G.H.; Chen, J.N.; Lai, X.P.; Su, Z.R.; Zeng, H.F.; Xie, Y., et al. Patchouli alcohol ameliorates dextran sodium sulfate-induced experimental colitis and suppresses tryptophan catabolism. *Pharmacological research* **2017**, *121*, 70-82.

52. Robinson, A.M.; Gondalia, S.V.; Karpe, A.V.; Eri, R.; Beale, D.J.; Morrison, P.D.; Palombo, E.A.; Nurgali, K. Fecal microbiota and metabolome in a mouse model of spontaneous chronic colitis: Relevance to human inflammatory bowel disease. *Inflammatory bowel diseases* **2016**, *22*, 2767-2787.

53. Schicho, R.; Nazyrova, A.; Shaykhutdinov, R.; Duggan, G.; Vogel, H.J.; Storr, M. Quantitative metabolomic profiling of serum and urine in DSS-induced ulcerative colitis of mice by 1H NMR spectroscopy. *Journal of proteome research* **2010**, *9*, 6265-6273.

54. Vassilyadi, P.; Harding, S.V.; Nitschmann, E.; Wykes, L.J. Experimental colitis and malnutrition differentially affect the metabolism of glutathione and related sulfhydryl metabolites in different tissues. *European Journal of Nutrition* **2016**, *55*, 1769-1776.

55. Wang, R.; Gu, X.; Dai, W.; Ye, J.; Lu, F.; Chai, Y.; Fan, G.; Gonzalez, F.J.; Duan, G.; Qi, Y. A lipidomics investigation into the intervention of celastrol in experimental colitis. *Mol Biosyst* **2016**, *12*, 1436-1444, doi:10.1039/c5mb00864f.

56. Willenberg, I.; Ostermann, A.I.; Giovannini, S.; Kershaw, O.; Von Keutz, A.; Steinberg, P.; Schebb, N.H. Effect of acute and chronic DSS induced colitis on plasma eicosanoid and oxylipin levels in the rat. *Prostaglandins and Other Lipid Mediators* **2015**, *120*, 155-160.

57. Zhang, X.; Choi, F.F.; Zhou, Y.; Leung, F.P.; Tan, S.; Lin, S.; Xu, H.; Jia, W.; Sung, J.J.; Cai, Z., et al. Metabolite profiling of plasma and urine from rats with TNBS-induced acute colitis using UPLC-ESI-QTOF-MS-based metabonomics--a pilot study. *The FEBS journal* **2012**, *279*, 2322-2338, doi:10.1111/j.1742-4658.2012.08612.x.

58. Zhang, W.; Liao, J.; Li, H.; Dong, H.; Bai, H.; Yang, A.; Hammock, B.D.; Yang, G.Y. Reduction of inflammatory bowel disease-induced tumor development in IL-10 knockout mice with soluble epoxide hydrolase gene deficiency. *Molecular Carcinogenesis* **2013**, *52*, 726-738.

59. Wirtz, S.; Popp, V.; Kindermann, M.; Gerlach, K.; Weigmann, B.; Fichtner-Feigl, S.; Neurath, M.F. Chemically induced mouse models of acute and chronic intestinal inflammation. *Nature protocols* **2017**, *12*, 1295-1309, doi:10.1038/nprot.2017.044.
