# Supplementary material for: Translational Potential of Metabolomics on Animal Models of Inflammatory Bowel Disease—A Systematic Critical Review
Source: Int J Mol Sci. 2020 May 29;21(11):3856. doi: 10.3390/ijms21113856 (PMC7312423; doi:10.3390/ijms21113856)
Supplement: Supplementary file 1 [file ijms-21-03856-s001.zip › Supplementary Table S10, S11_resubmission_proofread.docx]

**Supplementary Table S10a: Metabolite classifications – metabolites increased in inflammatory bowel disease (IBD)**

| **↑ in IBD in humans** | |  | **↑ in IBD in animals** | |
| --- | --- | --- | --- | --- |
| **Subclass** | **No. of different metabolites in each subclass** |  | **Subclass** | **No. of different metabolites in each subclass** |
| **Common in humans and animals** |  |  | **Common in humans and animals** |  |
| Amino acids, peptides, and analogues | 33 |  | Amino acids, peptides, and analogues | 43 |
| Carbohydrates and carbohydrate conjugates | 13 |  | Fatty acids and conjugates Carbohydrates and carbohydrate | 23 21 |
| Bile acids, alcohols and derivatives | 8 |  | conjugates |  |
| Carbonyl compounds | 8 |  | Eicosanoids | 18 |
| Fatty acids and conjugates | 8 |  | Glycerophosphocholines | 18 |
| Alcohols and polyols | 6 |  | Fatty acid esters | 13 |
| Dicarboxylic acids and derivatives | 4 |  | Phosphosphingolipids | 10 |
| Alkanes | 3 |  | Dicarboxylic acids and derivatives | 6 |
| Beta hydroxy acids and derivatives | 3 |  | Bile acids, alcohols and derivatives | 5 |
| Fatty acid esters | 3 |  | Phenylpropanoic acids | 5 |
| Short-chain keto acids and derivatives | 3 |  | Alcohols and polyols | 4 |
| Alpha hydroxy acids and derivatives | 2 |  | Amines | 4 |
| Amines | 2 |  | Beta hydroxy acids and derivatives | 4 |
| Benzoic acids and derivatives | 2 |  | Glycerophosphoserines | 4 |
| Carboxylic acids | 2 |  | Alpha hydroxy acids and derivatives | 3 |
| Cholestane steroids | 2 |  | Benzoic acids and derivatives | 3 |
| Eicosanoids | 2 |  | Carbonyl compounds | 3 |
| Glycerophosphocholines | 2 |  | Lineolic acids and derivatives | 3 |
| Lineolic acids and derivatives | 2 |  | Pyrimidines and pyrimidine derivatives | 3 |
| Quaternary ammonium salts | 2 |  | Quaternary ammonium salts | 3 |
| 1-hydroxy-2-unsubstituted benzenoids | 1 |  | Cholestane steroids | 2 |
| Alpha-keto acids and derivatives | 1 |  | Indolyl carboxylic acids and derivatives | 2 |
| Benzenediols | 1 |  | Monoterpenoids | 2 |
| Fatty alcohols | 1 |  | 1-hydroxy-2-unsubstituted benzenoids | 1 |
| Glycerophosphoserines | 1 |  | Alkanes | 1 |
| Indolyl carboxylic acids and derivatives | 1 |  | Alpha-keto acids and derivatives | 1 |
| Monoterpenoids | 1 |  | Benzenediols | 1 |
| Phenethylamines | 1 |  | Carboxylic acids | 1 |
| Phenylpropanoic acids | 1 |  | Fatty alcohols | 1 |
| Phosphosphingolipids | 1 |  | Phenethylamines | 1 |
| Pyrimidines and pyrimidine derivatives | 1 |  | Short-chain keto acids and derivatives | 1 |
| Ureas | 1 |  | Ureas | 1 |
|  |  |  |  |  |

**Supplementary Table S10b: Metabolite classifications – metabolites increased in inflammatory bowel disease (IBD)**

| **↑ in IBD in humans** | |  | **↑ in IBD in animals** | |
| --- | --- | --- | --- | --- |
| **Subclass** | **No. of different metabolites in each subclass** |  | **Subclass** | **No. of different metabolites in each subclass** |
| **Exclusively increased in humans** |  |  | **Exclusively increased in animals** |  |
| Glycosphingolipids | 14 |  | Purines and purine derivatives | 5 |
| Carboxylic acid derivatives | 4 |  | Benzene and substituted derivatives | 4 |
| Acyclic olefins | 3 |  | Pyrimidine ribonucleotides | 4 |
| Ketones | 3 |  | Glycerophosphoglycerols | 3 |
| Aldehydes | 2 |  | Ceramides | 2 |
| Other non-metal sulfides | 2 |  | Glycerophosphates | 2 |
| Toluenes | 2 |  | Glycosyl compounds | 2 |
| Acidic glycosphingolipids | 1 |  | Quinolones and derivatives | 2 |
| Acyclic alkanes | 1 |  | Steroid esters | 2 |
| Alkylthiols | 1 |  | Tricarboxylic acids and derivatives | 2 |
| Androstane steroids | 1 |  | Arylsulfates | 1 |
| Benzoyl derivatives Carbohydrates and carbohydrate | 1 1 |  | Benzenesulfonic acids and derivatives Bilirubins | 1 1 |
| derivatives |  |  | Carboximidic acids | 1 |
| Dialkyl ethers | 1 |  | Diradylglycerols | 1 |
| Dialkylthioethers | 1 |  | Diterpenoids | 1 |
| Dithioacetals | 1 |  | Ethers | 1 |
| Fatty acyl glycosides | 1 |  | Fatty amides | 1 |
| Glycerolipids | 1 |  | Flavin nucleotides | 1 |
| Glycolipid | 1 |  | Gamma butyrolactones | 1 |
| Heteroaromatic compounds | 1 |  | Gamma-keto acids and derivatives | 1 |
| Imidazoles | 1 |  | Glycerophosphoethanolamines | 1 |
| Indolecarboxylic acids and derivatives | 1 |  | Glycerophosphoinositols | 1 |
| Monoradylglycerols | 1 |  | Hydropyridines | 1 |
| Olefins | 1 |  | Hydroxysteroids | 1 |
| Organic cyanides | 1 |  | Isoflav-2-enes | 1 |
| Organic nitro compounds | 1 |  | Methoxyphenols | 1 |
| Organosulfonic acids and derivatives | 1 |  | Naphtalenes | 1 |
| Oxepanes | 1 |  | Non-metal phosphates | 1 |
| Oximes | 1 |  | Phosphate esters | 1 |
| Phenylacetaldehydes | 1 |  | Purine nucleosides | 1 |
| Phenylpropenes | 1 |  | Purine ribonucleotides | 1 |
| Phenylpyruvic acid derivatives | 1 |  | Pyridine carboxaldehydes | 1 |
| Piperidinones | 1 |  | Pyrimidine nucleosides | 1 |
| Polyols | 1 |  | Quinoline carboxylic acids | 1 |
| Styrenes | 1 |  | Steroids and steroid derivatives | 1 |
| 1-hydroxy-4-unsubstituted benzenoids | 1 |  | Thiophenol ethers | 1 |
| Pyridinecarboxylic acids and derivatives | 1 |  | Tryptamines and derivatives | 1 |
| Quinone and hydroquinone lipids | 1 |  |  |  |
| Saccharolipids | 1 |  |  |  |
| Sulfated steroids | 1 |  |  |  |
| Tertiary amines | 1 |  |  |  |
| Triradylglycerols | 1 |  |  |  |

**Supplementary Table S11a: Metabolite classifications – metabolites decreased in inflammatory bowel disease (IBD)**

| **↓ in IBD in humans** | | | |  | |  | | | **↓ in IBD in animals** | | | |  |
| --- | --- | --- | --- | --- | --- | --- | --- | --- | --- | --- | --- | --- | --- |
| **Subclass** | **No. of different metabolites in each subclass** | |  | |  | | **Subclass** | | | **No. of different metabolites in each subclass** | |  |  |
| **Common in humans and animals** | |  | |  | | | | **Common in humans and animals** | | |  | | |
| Amino acids, peptides, and analogues | 39 | |  | |  | | Glycerophosphocholines | | | 40 | |  |  |
| Carbohydrates and carbohydrate conjugates | 13 | |  | |  | | Amino acids, peptides, and analogues Fatty acids and conjugates | | | 36 22 | |  |  |
| Fatty acids and conjugates | 13 | |  | |  | | Fatty acid esters | | | 17 | |  |  |
| Carbonyl compounds  Carboxylic acids | 11 6 | |  | |  | | Carbohydrates and carbohydrate conjugates | | | 10 | |  |  |
| Fatty acid esters | 6 | |  | |  | | Beta hydroxy acids and derivatives | | | 7 | |  |  |
| Tricarboxylic acids and derivatives | 6 | |  | |  | | Eicosanoids | | | 5 | |  |  |
| Alcohols and polyols | 5 | |  | |  | | Indolyl carboxylic acids and derivatives | | | 5 | |  |  |
| Monoterpenoids | 5 | |  | |  | | Amines | | | 4 | |  |  |
| Alpha hydroxy acids and derivatives | 4 | |  | |  | | Dicarboxylic acids and derivatives | | | 4 | |  |  |
| Amines | 4 | |  | |  | | Purines and purine derivatives | | | 3 | |  |  |
| Dicarboxylic acids and derivatives | 4 | |  | |  | | Quaternary ammonium salts | | | 3 | |  |  |
| Glycerophosphocholines | 3 | |  | |  | | Tricarboxylic acids and derivatives | | | 3 | |  |  |
| Purines and purine derivatives | 3 | |  | |  | | Alcohols and polyols | | | 2 | |  |  |
| Beta hydroxy acids and derivatives | 2 | |  | |  | | Arylsulfates | | | 2 | |  |  |
| Quaternary ammonium salts | 2 | |  | |  | | Benzoic acids and derivatives | | | 2 | |  |  |
| Alkaloids and derivatives | 1 | |  | |  | | Carboxylic acids | | | 2 | |  |  |
| Arylsulfates | 1 | |  | |  | | Indoles | | | 2 | |  |  |
| Benzoic acids and derivatives | 1 | |  | |  | | Monoterpenoids | | | 2 | |  |  |
| Eicosanoids | 1 | |  | |  | | Alkaloids and derivatives | | | 1 | |  |  |
| Indoles | 1 | |  | |  | | Alpha hydroxy acids and derivatives | | | 1 | |  |  |
| Indolyl carboxylic acids and derivatives | 1 | |  | |  | | Carbonyl compounds | | | 1 | |  |  |
| Organosulfonic acids and derivatives | 1 | |  | |  | | Organosulfonic acids and derivatives | | | 1 | |  |  |
| Pyridinecarboxylic acids and derivatives | 1 | |  | |  | | Pyridinecarboxylic acids and derivatives | | | 1 | |  |  |
| Sesquiterpenoids | 1 | |  | |  | | Sesquiterpenoids | | | 1 | |  |  |
|  |  | |  | |  | |  | | |  | |  |  |
|  |  | |  | |  | |  | | |  | |  |  |
|  |  | |  | |  | |  | | |  | |  |  |
|  |  | |  | |  | |  | | |  | |  |  |

**Supplementary Table S11b: Metabolite classifications – metabolites decreased in inflammatory bowel disease (IBD)**

| **↓ in IBD in humans** | | | |  | |  | | | **↓ in IBD in animals** | | | |  |
| --- | --- | --- | --- | --- | --- | --- | --- | --- | --- | --- | --- | --- | --- |
| **Subclass** | **No. of different metabolites in each subclass** | |  | |  | | **Subclass** | | | **No. of different metabolites in each subclass** | |  |  |
| **Exclusively decreased in humans** | |  | |  | | | | **Exclusively decreased in animals** | | |  | | |
| Heteroaromatic compounds | 5 | |  | |  | | Glycerophosphoethanolamines | | | 5 | |  |  |
| Short-chain keto acids and derivatives | 3 | |  | |  | | Bile acids, alcohols and derivatives | | | 5 | |  |  |
| 1-hydroxy-2-unsubstituted benzenoids | 2 | |  | |  | | Lineolic acids and derivatives | | | 4 | |  |  |
| Carboxylic acid derivatives | 2 | |  | |  | | Guanidines | | | 3 | |  |  |
| Cholestane steroids | 2 | |  | |  | | Phenylpropanoic acids | | | 3 | |  |  |
| Cresols | 2 | |  | |  | | Phosphosphingolipids | | | 3 | |  |  |
| Dialkyldisulfides | 2 | |  | |  | | Gamma-keto acids and derivatives | | | 2 | |  |  |
| Other non-metal sulfides | 2 | |  | |  | | Glycerophosphates | | | 2 | |  |  |
| Phenylacetaldehydes | 2 | |  | |  | | Glycerophosphoserines | | | 2 | |  |  |
| Phenylpropenes | 2 | |  | |  | | Imidazoles | | | 2 | |  |  |
| Unsaturated aliphatic hydrocarbons Xylenes | 2 2 | |  | |  | | Medium-chain hydroxy acids and derivatives | | | 2 | |  |  |
| Alkanes | 1 | |  | |  | | Purine nucleosides | | | 2 | |  |  |
| Alkylthiols | 1 | |  | |  | | Pyrimidine nucleosides | | | 2 | |  |  |
| Alpha,beta-unsaturated carbonyl compounds | 1 | |  | |  | | Triradylcglycerols 5'-deoxy-5'-thionucleosides | | | 2 1 | |  |  |
| Androstane steroids | 1 | |  | |  | | Biphenyls and derivatives | | | 1 | |  |  |
| Bilirubins | 1 | |  | |  | | Diradylglycerols | | | 1 | |  |  |
| Cinnamaldehydes | 1 | |  | |  | | Diterpenoids | | | 1 | |  |  |
| Cumenes | 1 | |  | |  | | Estrane steroids | | | 1 | |  |  |
| Dialkylthioethers | 1 | |  | |  | | Fatty acyl thioesters | | | 1 | |  |  |
| Fatty alcohols | 1 | |  | |  | | Glycerophosphoglycerols | | | 1 | |  |  |
| Glycerophosphoinositols | 1 | |  | |  | | Hydroxycinnamic acids and derivatives | | | 1 | |  |  |
| Glycosphingolipids | 1 | |  | |  | | Indolines | | | 1 | |  |  |
| Homogeneous other non-metal compounds | 1 | |  | |  | | Isosorbides Phenanthrenes and derivatives | | | 1 1 | |  |  |
| Non-metal phosphates | 1 | |  | |  | | Phenylacetic acids | | | 1 | |  |  |
| Olefins | 1 | |  | |  | | Polyols | | | 1 | |  |  |
| Organic nitro compounds | 1 | |  | |  | | Porphyrins | | | 1 | |  |  |
| Organic trisulfides | 1 | |  | |  | | Purine 2'-deoxyribonucleosides | | | 1 | |  |  |
| Phenylpyruvic acid derivatives | 1 | |  | |  | | Purine ribonucleotides | | | 1 | |  |  |
| Phosphate esters | 1 | |  | |  | | Pyrimidine ribonucleotides | | | 1 | |  |  |
| Pyrimidine nucleotide sugars | 1 | |  | |  | | Pyrimidines and pyrimidine derivatives | | | 1 | |  |  |
| Sulfones | 1 | |  | |  | | Quinoline carboxylic acids | | | 1 | |  |  |
| Toluenes | 1 | |  | |  | | Retinoids | | | 1 | |  |  |
| Ureas | 1 | |  | |  | | Short-chain hydroxy acids and derivatives | | | 1 | |  |  |
|  |  | |  | |  | | Steroidal glycosides | | | 1 | |  |  |
|  |  | |  | |  | |  | | |  | |  |  |
|  |  | |  | |  | |  | | |  | |  |  |
|  |  | |  | |  | |  | | |  | |  |  |
|  |  | |  | |  | |  | | |  | |  |  |
|  |  | |  | |  | |  | | |  | |  |  |
|  |  | |  | |  | |  | | |  | |  |  |
|  |  | |  | |  | |  | | |  | |  |  |
