# Supplementary material for: Translational Potential of Metabolomics on Animal Models of Inflammatory Bowel Disease—A Systematic Critical Review
Source: Int J Mol Sci. 2020 May 29;21(11):3856. doi: 10.3390/ijms21113856 (PMC7312423; doi:10.3390/ijms21113856)
Supplement: Supplementary file 1 [file ijms-21-03856-s001.zip › Supplementary Table S12_resubmission_proofread.docx]

**Supplementary Table S12: Literature search strategy**

| **Group 1 - Embase** | **Group 1 - PubMed** |
| --- | --- |
| **Search terms:**  metabolomics  metabolome  metabolite  **Free text search words:**  metabolomic*  metabonomic*  metabolome*  metabolic profil*  metabolite* | **Search terms (MeSH):**  metabolomics  metabolome  **Free text search words:**  metabolomic*  metabonomic*  metabolome*  metabolic profil*  metabolite* |

| **Group 2 - Embase** | **Group 2 – PubMed** |
| --- | --- |
| **Search terms:**  mass spectrometry (exp)  *NT: matrix-assisted laser desorption-ionization mass spectrometry (exp)*  *NT: tandem mass spectrometry (exp)*  nuclear magnetic resonance spectroscopy  **Free text search words:**  mass spectrometry  mass spectrometric analysis  mass spectrum  electrospray MS  ESI MS  ICR MS  liquid chromatography-mass spectrometry  HPLC-MS  LC-MS  LC-MS MS  UPLC-MS  mass fragmentography  gas chromatography-mass spectrometry  MALDI TOF MS  MALDI TOFMS  magnetic resonance spectroscopy  biomolecular nuclear magnetic resonance  nuclear magnetic resonance spectrometry  nmr  nmr spectroscopy  MR spectroscopy  nuclear magnetic resonance spectral analysis  nuclear magnetic resonance spectrum  nuclear magnetic resonance | **Search terms (MeSH):**  mass spectrometry  biomolecular nuclear magnetic resonance  **Free text search words:**  "mass spectrometry"  "mass spectrometric analysis"  "mass spectrum"  "electrospray MS"  "ESI MS"  "ICR MS"  "liquid chromatography-mass spectrometry"  "HPLC-MS"  "LC-MS"  "LC-MS MS"  "UPLC-MS"  "mass fragmentography"  "gas chromatography-mass spectrometry"  "MALDI TOF MS"  "MALDI TOFMS"  "magnetic resonance spectroscopy"  "biomolecular nuclear magnetic resonance"  "nuclear magnetic resonance spectrometry"  nmr  "nmr spectroscopy"  "mr spectroscopy"  "nuclear magnetic resonance spectral analysis"  "nuclear magnetic resonance spectrum"  “nuclear magnetic resonance” |

| **Group 3 - Embase** | **Group 3 - PubMed** |
| --- | --- |
| **Search terms:**  inflammatory bowel diseases (exp)  *NT: crohn disease (exp)*  *NT: ulcerative colitis (exp)*  colitis  *NT: enterocolitis*  *NT: experimental colitis (exp)*  *NT: pancolitis*  *NT: proctitis*  *NT: proctocolitis*  **Free text search words:**  inflammatory bowel disease*  crohn disease  crohn’s disease  enteritis regionalis  morbus crohn  regional enteritis  regional enterocolitis  colitis  ulcerative colorectitis  ulcerative proctocolitis  colon inflammation  colon inflammatory disease  enterocolitis  experimental colonic inflammation  experimentally induced colonic inflammation  pancolitis  proctitis  rectitis  proctocolitis  colorectitis  rectocolitis  proctosigmoiditis  rectosigmoiditis | **Search terms:**  inflammatory bowel diseases  colitis (MeSH:NoExp)  enterocolitis (MeSH:NoExp])  proctitis  **Free text search words:**  inflammatory bowel disease*  "crohn disease"  "crohn's disease"  "enteritis regionalis"  "morbus crohn"  "regional enteritis"  "regional enterocolitis"  colitis  "ulcerative colorectitis"  "ulcerative proctocolitis"  "colon inflammation"  "colon inflammatory disease"  enterocolitis  "experimental colonic inflammation"  "experimentally induced colonic inflammation"  pancolitis  proctitis  rectitis  proctocolitis  colorectitis  rectocolitis  proctosigmoiditis  rectosigmoiditis |

NT = narrower term
