# Supplementary material for: Translational Potential of Metabolomics on Animal Models of Inflammatory Bowel Disease—A Systematic Critical Review
Source: Int J Mol Sci. 2020 May 29;21(11):3856. doi: 10.3390/ijms21113856 (PMC7312423; doi:10.3390/ijms21113856)
Supplement: Supplementary file 1 [file ijms-21-03856-s001.zip › Supplementary Table S3, S4_resubmission_proofread.docx]

**Supplementary Table S3: Quality assessment of included human studies**

| **Reference** | **1** | **2** | **3** | **4** | **5** | **6** | **7** | **8** | **9** | **10** | **11** | **12** | **13** | **14** | **15** | **16** | **17** | **18** | **19** | **20** | **21** | **Total score (%)*** |
| --- | --- | --- | --- | --- | --- | --- | --- | --- | --- | --- | --- | --- | --- | --- | --- | --- | --- | --- | --- | --- | --- | --- |
| [1] | N | Y | Y | Y | Y | Y | Y | Y | Y | Y | Y | N | Y | N/A | Y | Y | Y | Y | Unclear | N | Y | 80.0 |
| [2] | Y | Y | Y | Y | N | Y | Y | Y | Y | Y | N | N | Unclear | N/A | Y | N/A | Y | N | N/A | Y | Y | 72.2 |
| [3] | N | N | Y | N | Y | Y | N | Y | Y | Y | Y | Y | Unclear | Y | Y | Y | N | N | Y | Y | Y | 66.7 |
| [4] | N | Y | Y | Y | Y | Y | N | Unclear | Y | Y | Y | N | N | N/A | Y | N/A | Y | N | N/A | Y | Y | 66.7 |
| [5] | Y | N | Y | N | Y | Y | N | Unclear | N | Y | Y | Y | Unclear | Y | Y | N/A | Y | N | N/A | Y | Y | 63.2 |
| [6] | N | Y | Y | Y | Y | Y | N | Unclear | Unclear | Y | Y | N | Unclear | Y | Y | Y | Y | N | Y | Y | N | 61.9 |
| [7] | Y | N | Y | N | Y | Y | Y | Y | N | Y | N | Y | Unclear | Y | Y | Y | N | N | N/A | N | Y | 60.0 |
| [8] | N | N | Y | N | N | Y | N | Unclear | Y | Y | Y | N | Unclear | Y | Y | N/A | Y | Y | Y | Y | Y | 60.0 |
| [9] | N | Y | Y | N | N | Y | N | Unclear | N | Y | Y | Y | Unclear | Y | Y | Y | Y | N | N/A | Y | Y | 60.0 |
| [10] | N | N | Y | N | N | Y | N | Unclear | Y | Y | Y | N | Unclear | N/A | Y | N/A | Y | Y | N/A | Y | Y | 55.6 |
| [11] | N | N | Y | N | Y | Y | N | Unclear | Y | Y | Y | N | Unclear | N/A | Y | N/A | Y | N | N/A | Y | Y | 55.6 |
| [12] | N | N | Y | N | N | N | Y | Y | Y | Y | Y | N | Unclear | N/A | Y | N/A | N | Y | N/A | Y | N/A | 52.9 |
| [13] | N | N | Y | N | N | Y | N | Unclear | Y | Y | Y | N | Unclear | N/A | Y | N/A | Y | N | Y | Y | Y | 52.6 |
| [14] | N | N | Y | N | N | Y | N | Unclear | Y | Y | Y | N | Unclear | Y | Y | N/A | Y | N | N/A | Y | Y | 52.6 |
| [15] | N | N | Y | N | N | Y | N | Unclear | Y | Y | Y | N | Unclear | N/A | Y | Y | Y | N | N/A | Y | Y | 52.6 |
| [16] | N | N | Y | N | Y | Y | N | Unclear | Y | Y | Y | N | Unclear | N/A | Y | N/A | Y | N | N/A | Y | N | 50.0 |
| [17] | N | N | Y | N | Y | Y | N | Unclear | N | Y | Y | N | N | N/A | Y | N/A | Y | N | N/A | Y | Y | 50.0 |
| [18] | N | N | Y | N | N | Y | N | Unclear | N | Y | Y | Y | N | Y | Y | N/A | Y | N | N/A | Y | N/A | 50.0 |
| [19] | N | N | Y | N | N | Y | N | Unclear | Y | Y | Y | N | Unclear | Y | Y | Y | Y | N | N/A | Y | N | 50.0 |
| [20] | N | N | Y | N | N | Y | N | Unclear | N | Y | Y | N | Unclear | Y | Y | N/A | Y | N | N/A | Y | Y | 47.4 |
| [21] | Y | N | Y | N | Y | Y | N | Unclear | N | Y | Y | N | Unclear | N/A | Y | N/A | N | N | N/A | Y | N/A | 47.1 |
| [22] | N | N | N | N | N | Y | N | Unclear | N | Y | Y | N | Y | N/A | Y | N/A | Y | Y | N/A | Y | N/A | 47.1 |
| [23] | N | Y | N | Y | N | Y | N | Unclear | Y | Y | Y | N | Unclear | N/A | Y | N/A | N | N | N/A | Y | N | 44.4 |
| [24] | N | N | N | N | Y | Y | N | Unclear | N | Y | Y | N | Unclear | N/A | Y | N/A | Y | N | N/A | Y | Y | 44.4 |
| [25] | N | N | N | N | N | Y | N | Unclear | N | Y | Y | N | Unclear | N/A | Y | N/A | Y | Y | N/A | Y | Y | 44.4 |
| [26] | N | N | Y | N | Unclear | Y | N | Unclear | N | Y | Y | N | Unclear | N/A | Y | N/A | Y | N | Y | Y | N/A | 44.4 |
| [27] | N | N | Y | N | Unclear | Y | N | Unclear | N | Y | Y | N | Unclear | N/A | Y | N/A | Y | N | N/A | Y | N/A | 41.2 |
| [28] | N | N | Y | N | N | Y | N | Unclear | N | Y | Y | N | Unclear | Y | Y | N/A | Y | N | N | Y | N | 40.0 |
| [29] | N | N | Y | N | N | Y | N | N | N | Y | Y | N | Unclear | N | Y | N/A | Y | N | N | Y | N/A | 36.8 |
| [30] | N | N | Y | N | N | N | N | Unclear | N | N | Y | N | Unclear | N/A | Y | Y | Y | N | N/A | Y | Y | 36.8 |
| [31] | N | N | Y | N | N | Y | Y | Y | N | Y | N | N | Unclear | N/A | N | N/A | Y | N | N | Y | N | 36.8 |
| [32] | N | N | Y | N | N | Y | Y | Unclear | N | N | Y | N | Unclear | N/A | Y | N/A | N | N | N/A | Y | N/A | 35.3 |
| [33] | N | N | N | N | N | Y | N | Unclear | N | Y | Y | N | Unclear | N/A | Y | N/A | N | N | N/A | Y | N/A | 29.4 |
| No. of studies fulfilling parameters | 4 | 6 | 28 | 5 | 11 | 31 | 6 | 6 | 14 | 31 | 30 | 5 | 2 | 11 | 32 | 8 | 26 | 6 | 5 | 31 | 18 |  |

*Total score was calculated as number of ‘Y’ out of the total number of relevant parameters (N/A = irrelevant) in %. N = no; N/A = not applicable; Y = yes.

|  | **Description of quality assessment parameters – human studies** |
| --- | --- |
| **1** | Was there a power calculation or was the number of study subjects justified? |
| **2** | Was the study population clearly described? (age, gender, and ethnicity as minimum) |
| **3** | Was disease activity level clearly defined using a CD and/or UC activity score? |
| **4** | Was the control group population clearly described? (age, gender, and ethnicity as minimum) |
| **5** | Were case and control study subjects matched for at least age and sex? |
| **6** | Was the type of sample used fully described? |
| **7** | Was time of day for sampling stated? |
| **8** | Was time of day for sampling the same for cases and controls? |
| **9** | Were the procedures and timing of biological sample collection with respect to clinical factors described with enough detail? |
| **10** | Were handling of specimens and pre-analytical procedures reported in sufficient detail and similar for the whole sample? And if differences in procedures were reported, were their effect on the results assessed? |
| **11** | Were extraction and detection methods described in sufficient detail to permit replication of the test? (including machine settings) |
| **12** | Was the outcome measure validated? |
| **13** | Was the outcome measurement reproducible? |
| **14** | Was the spectrum database stated? |
| **15** | Was the metabolite identification method explained? |
| **16** | Was the procedure for unidentified compounds disclosed? |
| **17** | Was the data analysis tool/software disclosed? |
| **18** | Was it reported whether or not the outcome assessors were blinded to the health status of participants? |
| **19** | If attrition (overall or differential nonresponse, dropout, loss to follow-up, or exclusion of participants) was a concern, were missing data handled appropriately (e.g., intention-to-treat analysis and imputation)? |
| **20** | Were uninterpretable/indeterminate/intermediate results reported? |
| **21** | Is it likely that overfitting was avoided? |

**Supplementary Table S4: Quality assessment of included animal studies**

| **Reference** | **1** | **2** | **3** | **4** | **5** | **6** | **7** | **8** | **9** | **10** | **11** | **12** | **13** | **14** | **15** | **16** | **17** | **18** | **19** | **20** | **Total score (%)*** |
| --- | --- | --- | --- | --- | --- | --- | --- | --- | --- | --- | --- | --- | --- | --- | --- | --- | --- | --- | --- | --- | --- |
| [34] | N | Y | Y | Y | Y | Y | Y | Y | Y | Unclear | Y | Y | Y | Y | N/A | Unclear | N | Y | Y | N/A | 77.8 |
| [35] | N | N | Y | Y | Y | Y | Y | Y | Y | Y | N/A | Y | N/A | Y | N/A | Unclear | N | N/A | Y | N/A | 73.3 |
| [36] | N | Y | Y | Y | Y | Y | Y | Y | N | Unclear | Y | Y | N/A | Y | N/A | Unclear | N | N/A | Y | Y | 70.6 |
| [37] | N | Y | Y | Y | Y | Y | Y | Y | Y | Unclear | N | Y | Y | Y | N/A | Unclear | N | N/A | Y | N | 66.7 |
| [38] | N | Y | Y | Y | Y | Y | Y | Y | N | Unclear | Y | Y | N/A | Y | N/A | Unclear | N | N/A | Y | N | 64.7 |
| [39] | N | Y | Y | Y | N | Unclear | Y | Y | N | Unclear | N/A | Y | Y | Y | Y | Unclear | N | Y | Y | Y | 63.2 |
| [40] | N | Y | Y | Y | N | Unclear | Y | Y | N | Unclear | Y | Y | N/A | Y | N/A | Unclear | N | N/A | Y | Y | 58.8 |
| [41] | N | Y | Y | Y | N | Unclear | Y | Y | N | Unclear | N/A | Y | N/A | Y | N/A | Unclear | N | N/A | Y | Y | 56.3 |
| [42] | N | Y | Y | Y | N | Unclear | Y | Y | N | Unclear | N/A | Y | N/A | Y | N/A | Unclear | N | N/A | Y | N/A | 53.3 |
| [43] | N | N | Y | Y | N | Unclear | Y | Y | N | Unclear | Y | Y | N/A | Y | Y | Unclear | N | Y | Y | N | 52.6 |
| [44] | N | Y | Y | Y | N | Unclear | Y | Y | N | Unclear | N/A | Y | N/A | Y | N/A | Unclear | N | N/A | Y | N | 50.0 |
| [45] | N | Y | Y | Y | N | Unclear | Y | Y | N | Unclear | N | Y | N/A | Y | N/A | Unclear | N | N/A | Y | N/A | 50.0 |
| [46] | N | N | Y | Y | N | Unclear | Y | Y | N | Unclear | Y | Y | N/A | Y | Unclear | Unclear | N | N/A | Y | N | 44.4 |
| [47] | N | N | Unclear | Y | N | Unclear | Y | Y | N | Unclear | Y | Y | Y | Y | N/A | Unclear | N | N/A | Y | N | 44.4 |
| [48] | N | Y | Y | Y | N | Unclear | Y | Y | Y | Unclear | N | N | N | Y | N/A | Unclear | N | N/A | Y | N | 44.4 |
| [19] | N | N | Y | Y | N | Unclear | Y | Y | N | Unclear | Y | Y | N/A | Y | Unclear | Unclear | N | Unclear | Y | N | 42.1 |
| [49] | N | N | Y | N | N | Unclear | N | Y | Y | Unclear | Y | Y | Y | Y | Unclear | Unclear | N | Unclear | Y | N | 40.0 |
| [50] | N | N | Unclear | Y | N | Unclear | Y | Y | N | Unclear | N/A | Y | N/A | Y | N/A | Unclear | N | N/A | Y | N/A | 40.0 |
| [51] | N | N | Unclear | Y | N | Unclear | Y | Y | N | Unclear | N/A | Y | N/A | Y | N/A | Unclear | N | N/A | Y | N/A | 40.0 |
| [52] | N | Y | Y | Y | N | Unclear | Y | N | N | Unclear | N/A | N/A | N/A | Y | N | Unclear | N | N/A | Y | N/A | 40.0 |
| [53] | N | N | Y | Y | N | Unclear | Y | Y | N | Unclear | N/A | Y | N/A | N | N/A | Unclear | N | N/A | Y | N/A | 40.0 |
| [54] | N | N | Y | N | N | Unclear | Y | Y | N | Unclear | N/A | Y | N/A | Y | Unclear | Unclear | N | Unclear | Y | Y | 38.9 |
| [55] | N | N | Unclear | Y | N | Unclear | Y | Y | N | Unclear | N/A | Y | N/A | Y | N/A | Unclear | N | N/A | N | Y | 37.5 |
| [56] | N | N | Y | Unclear | N | Unclear | Unclear | Y | N | Unclear | Y | Y | N/A | Y | Unclear | Unclear | N | N/A | Y | N | 33.3 |
| [57] | N | N | Unclear | Y | N | Unclear | Y | Y | N | Unclear | Y | Y | N | Y | Unclear | Unclear | N | N | N | N/A | 31.6 |
| [58] | N | N | Unclear | N | N | Unclear | N | Y | N | Unclear | Y | Y | N/A | Y | N/A | Unclear | N | N/A | Y | N/A | 31.3 |
| No. of studies fulfilling parameters | 0 | 12 | 20 | 22 | 5 | 5 | 23 | 25 | 5 | 1 | 12 | 24 | 5 | 25 | 2 | 0 | 0 | 3 | 24 | 6 |  |

*Total score was calculated as number of ‘Y’ out of the total number of relevant parameters (N/A = irrelevant) in %. N = no; N/A = not applicable; Y = yes.

|  | **Description of quality assessment parameters – animal studies** |
| --- | --- |
| **1** | Was there a power calculation or was the number of study subjects justified |
| **2** | Was the study population clearly described |
| **3** | Were case and control study subjects matched for at least age and sex |
| **4** | Was the type of sample used fully described |
| **5** | Was time of day for sampling stated |
| **6** | Was time of day for sampling the same for both cases and controls |
| **7** | Were handling of specimens and pre-analytical procedures reported in sufficient detail and similar for the whole sample? And if differences in procedures were reported, was their effect on the results assessed? |
| **8** | Were extraction and detection methods described in sufficient detail to permit replication of the test? (including machine settings) |
| **9** | Was the outcome measure validated? |
| **10** | Was the outcome measurement reproducible? |
| **11** | Was the spectrum database stated |
| **12** | Was the metabolite identification method explained? |
| **13** | Was the procedure for unidentified compounds disclosed? |
| **14** | Was the data analysis tool/software disclosed? |
| **15** | *Were losses and exclusions reported* |
| **16** | Were the caregivers and/or investigators blinded from knowledge of which intervention each animal received during the experiment? |
| **17** | Was it reported whether or not the outcome assessors were blinded to the health status of animals? |
| **18** | Were incomplete outcome data adequately addressed? |
| **19** | Were uninterpretable/indeterminate/intermediate results reported (selective outcome reporting) |
| **20** | Is it likely that overfitting was avoided? |

**References**

1. Williams, H.R.T.; Cox, I.J.; Walker, D.G.; North, B.V.; Patel, V.M.; Marshall, S.E.; Jewell, D.P.; Ghosh, S.; Thomas, H.J.W.; Teare, J.P., et al. Characterization of inflammatory bowel disease with urinary metabolic profiling. *American Journal of Gastroenterology* **2009**, *104*, 1435-1444.

2. Patel, N.; Alkhouri, N.; Eng, K.; Cikach, F.; Mahajan, L.; Yan, C.; Grove, D.; Rome, E.S.; Lopez, R.; Dweik, R.A. Metabolomic analysis of breath volatile organic compounds reveals unique breathprints in children with inflammatory bowel disease: A pilot study. *Alimentary Pharmacology and Therapeutics* **2014**, *40*, 498-507.

3. Alonso, A.; Julia, A.; Vinaixa, M.; Domenech, E.; Fernandez-Nebro, A.; Canete, J.D.; Ferrandiz, C.; Tornero, J.; Gisbert, J.P.; Nos, P., et al. Urine metabolome profiling of immune-mediated inflammatory diseases. *BMC medicine* **2016**, *14*, 133, doi:10.1186/s12916-016-0681-8.

4. Hicks, L.C.; Huang, J.; Kumar, S.; Powles, S.T.; Orchard, T.R.; Hanna, G.B.; Williams, H.R. Analysis of Exhaled Breath Volatile Organic Compounds in Inflammatory Bowel Disease: A Pilot Study. *Journal of Crohn's & colitis* **2015**, *9*, 731-737.

5. Kohashi, M.; Nishiumi, S.; Ooi, M.; Yoshie, T.; Matsubara, A.; Suzuki, M.; Hoshi, N.; Kamikozuru, K.; Yokoyama, Y.; Fukunaga, K., et al. A novel gas chromatography mass spectrometry-based serum diagnostic and assessment approach to ulcerative colitis. *Journal of Crohn's and Colitis* **2014**, *8*, 1010-1021.

6. Ahmed, I.; Greenwood, R.; Costello, B.; Ratcliffe, N.; Probert, C.S. Investigation of faecal volatile organic metabolites as novel diagnostic biomarkers in inflammatory bowel disease. *Alimentary Pharmacology and Therapeutics* **2016**, *43*, 596-611.

7. Bodelier, A.G.L.; Smolinska, A.; Baranska, A.; Dallinga, J.W.; Mujagic, Z.; Vanhees, K.; Van Den Heuvel, T.; Masclee, A.A.M.; Jonkers, D.; Pierik, M.J., et al. Volatile organic compounds in exhaled air as novel marker for disease activity in Crohn's disease: A metabolomic approach. *Inflammatory bowel diseases* **2015**, *21*, 1776-1785.

8. De Preter, V.; Joossens, M.; Ballet, V.; Shkedy, Z.; Rutgeerts, P.; Vermeire, S.; Verbeke Phd, K. Metabolic profiling of the impact of oligofructose-enriched inulin in Crohn's disease patients: a double-blinded randomized controlled trial. *Clinical and translational gastroenterology* **2013**, *4*, e30, doi:10.1038/ctg.2012.24.

9. Jacobs, J.P.; Goudarzi, M.; Singh, N.; Tong, M.; McHardy, I.H.; Ruegger, P.; Asadourian, M.; Moon, B.H.; Ayson, A.; Borneman, J., et al. A Disease-Associated Microbial and Metabolomics State in Relatives of Pediatric Inflammatory Bowel Disease Patients. *Cellular and molecular gastroenterology and hepatology* **2016**, *2*, 750-766.

10. Schicho, R.; Shaykhutdinov, R.; Ngo, J.; Nazyrova, A.; Schneider, C.; Panaccione, R.; Kaplan, G.G.; Vogel, H.J.; Storr, M. Quantitative metabolomic profiling of serum, plasma, and urine by 1H NMR spectroscopy discriminates between patients with inflammatory bowel disease and healthy individuals. *Journal of proteome research* **2012**, *11*, 3344-3357.

11. Zhang, Y.; Lin, L.; Xu, Y.; Lin, Y.; Jin, Y.; Zheng, C. 1H NMR-based spectroscopy detects metabolic alterations in serum of patients with early-stage ulcerative colitis. *Biochemical and biophysical research communications* **2013**, *433*, 547-551.

12. Williams, H.R.; Cox, I.J.; Walker, D.G.; Cobbold, J.F.; Taylor-Robinson, S.D.; Marshall, S.E.; Orchard, T. Differences in gut microbial metabolism are responsible for reduced hippurate synthesis in Crohn's disease. *Gastroenterology* **2010**, *138*, S579.

13. Bjerrum, J.T.; Wang, Y.; Hao, F.; Coskun, M.; Ludwig, C.; Gunther, U.; Nielsen, O.H. Metabonomics of human fecal extracts characterize ulcerative colitis, Crohn's disease and healthy individuals. *Metabolomics : Official journal of the Metabolomic Society* **2015**, *11*, 122-133, doi:10.1007/s11306-014-0677-3.

14. De Preter, V.; Machiels, K.; Joossens, M.; Arijs, I.; Matthys, C.; Vermeire, S.; Rutgeerts, P.; Verbeke, K. Faecal metabolite profiling identifies medium-chain fatty acids as discriminating compounds in IBD. *Gut* **2015**, *64*, 447-458.

15. Le Gall, G.; Noor, S.O.; Ridgway, K.; Scovell, L.; Jamieson, C.; Johnson, I.T.; Colquhoun, I.J.; Kemsley, E.K.; Narbad, A. Metabolomics of fecal extracts detects altered metabolic activity of gut microbiota in ulcerative colitis and irritable bowel syndrome. *Journal of proteome research* **2011**, *10*, 4208-4218.

16. Machiels, K.; Joossens, M.; Sabino, J.; De Preter, V.; Arijs, I.; Eeckhaut, V.; Ballet, V.; Claes, K.; Van Immerseel, F.; Verbeke, K., et al. A decrease of the butyrate-producing species roseburia hominis and faecalibacterium prausnitzii defines dysbiosis in patients with ulcerative colitis. *Gut* **2014**, *63*, 1275-1283.

17. Stephens, N.S.; Siffledeen, J.; Su, X.; Murdoch, T.B.; Fedorak, R.N.; Slupsky, C.M. Urinary NMR metabolomic profiles discriminate inflammatory bowel disease from healthy. *Journal of Crohn's and Colitis* **2013**, *7*, e42-e48.

18. Yau, Y.Y.; Leong, R.W.L.; Shin, S.; Bustamante, S.; Pickford, R.; Hejazi, L.; Campbell, B.; Wasinger, V.C. Bimodal plasma metabolomics strategy identifies novel inflammatory metabolites in inflammatory bowel diseases. *Discovery medicine* **2014**, *18*, 113-124.

19. Shiomi, Y.; Nishiumi, S.; Ooi, M.; Hatano, N.; Shinohara, M.; Yoshie, T.; Kondo, Y.; Furumatsu, K.; Shiomi, H.; Kutsumi, H., et al. GCMS-based metabolomic study in mice with colitis induced by dextran sulfate sodium. *Inflammatory bowel diseases* **2011**, *17*, 2261-2274.

20. Jansson, J.; Willing, B.; Lucio, M.; Fekete, A.; Dicksved, J.; Halfvarson, J.; Tysk, C.; Schmitt-Kopplin, P. Metabolomics reveals metabolic biomarkers of Crohn's disease. *PloS one* **2009**, *4*, e6386, doi:10.1371/journal.pone.0006386.

21. Cracowski, J.L.; Bonaz, B.; Bessard, G.; Bessard, J.; Anglade, C.; Fournet, J. Increased urinary F2-isoprostanes in patients with Crohn's disease. *American Journal of Gastroenterology* **2002**, *97*, 99-103.

22. Johnson, J.C.; Schmidt, C.R.; Shrubsole, M.J.; Billheimer, D.D.; Joshi, P.R.; Morrow, J.D.; Heslin, M.J.; Washington, M.K.; Ness, R.M.; Zheng, W., et al. Urine PGE-M: A Metabolite of Prostaglandin E2 as a Potential Biomarker of Advanced Colorectal Neoplasia. *Clinical Gastroenterology and Hepatology* **2006**, *4*, 1358-1365.

23. Rieder, F.; Kurada, S.; Grove, D.; Cikach, F.; Lopez, R.; Patel, N.; Singh, A.; Alkhouri, N.; Shen, B.; Brzezinski, A., et al. A Distinct Colon-Derived Breath Metabolome is Associated with Inflammatory Bowel Disease, but not its Complications. *Clinical and translational gastroenterology* **2016**, *7*, e201, doi:10.1038/ctg.2016.57.

24. Fathi, F.; Majari-Kasmaee, L.; Mani-Varnosfaderani, A.; Kyani, A.; Rostami-Nejad, M.; Sohrabzadeh, K.; Naderi, N.; Zali, M.R.; Rezaei-Tavirani, M.; Tafazzoli, M., et al. 1H NMR based metabolic profiling in Crohn's disease by random forest methodology. *Magnetic resonance in chemistry : MRC* **2014**, *52*, 370-376.

25. Marchesi, J.R.; Holmes, E.; Khan, F.; Kochhar, S.; Scanlan, P.; Shanahan, F.; Wilson, I.D.; Wang, Y. Rapid and noninvasive metabonomic characterization of inflammatory bowel disease. *Journal of proteome research* **2007**, *6*, 546-551.

26. Sharma, U.; Singh, R.R.; Ahuja, V.; Makharia, G.K.; Jagannathan, N.R. Similarity in the metabolic profile in macroscopically involved and un-involved colonic mucosa in patients with inflammatory bowel disease: An in vitro proton (1H) MR spectroscopy study. *Magnetic Resonance Imaging* **2010**, *28*, 1022-1029.

27. Balasubramanian, K.; Kumar, S.; Singh, R.R.; Sharma, U.; Ahuja, V.; Makharia, G.K.; Jagannathan, N.R. Metabolism of the colonic mucosa in patients with inflammatory bowel diseases: an in vitro proton magnetic resonance spectroscopy study. *Magnetic Resonance Imaging* **2009**, *27*, 79-86.

28. Ooi, M.; Nishiumi, S.; Yoshie, T.; Shiomi, Y.; Kohashi, M.; Fukunaga, K.; Nakamura, S.; Matsumoto, T.; Hatano, N.; Shinohara, M., et al. GC/MS-based profiling of amino acids and TCA cycle-related molecules in ulcerative colitis. *Inflammation Research* **2011**, *60*, 831-840.

29. Sewell, G.W.; Hannun, Y.A.; Han, X.; Koster, G.; Bielawski, J.; Goss, V.; Smith, P.J.; Rahman, F.Z.; Vega, R.; Bloom, S.L., et al. Lipidomic profiling in Crohn's disease: abnormalities in phosphatidylinositols, with preservation of ceramide, phosphatidylcholine and phosphatidylserine composition. *The international journal of biochemistry & cell biology* **2012**, *44*, 1839-1846, doi:10.1016/j.biocel.2012.06.016.

30. Dawiskiba, T.; Deja, S.; Mulak, A.; Zabek, A.; Jawien, E.; Pawelka, D.; Banasik, M.; Mastalerz-Migas, A.; Balcerzak, W.; Kaliszewski, K., et al. Serum and urine metabolomic fingerprinting in diagnostics of inflammatory bowel diseases. *World journal of gastroenterology : WJG* **2014**, *20*, 163-174, doi:10.3748/wjg.v20.i1.163.

31. Martin, F.P.; Ezri, J.; Cominetti, O.; Da Silva, L.; Kussmann, M.; Godin, J.P.; Nydegger, A. Urinary metabolic phenotyping reveals differences in the metabolic status of healthy and inflammatory bowel disease (IBD) children in relation to growth and disease activity. *International journal of molecular sciences* **2016**, *17*, no pagination.

32. Iwamoto, J.; Saito, Y.; Honda, A.; Miyazaki, T.; Ikegami, T.; Matsuzaki, Y. Bile acid malabsorption deactivates pregnane x receptor in patients with Crohn's Disease. *Inflammatory bowel diseases* **2013**, *19*, 1278-1284.

33. Thyssen, E.; Turk, J.; Bohrer, A.; Stenson, W.F. Quantification of distinct molecular species of platelet activating factor in ulcerative colitis. *Lipids* **1996**, *31*, S255-S259.

34. Lin, H.M.; Edmunds, S.J.; Helsby, N.A.; Ferguson, L.R.; Rowan, D.D. Nontargeted urinary metabolite profiling of a mouse model of crohn's disease. *Journal of proteome research* **2009**, *8*, 2045-2057.

35. Hou, W.; Zhong, D.; Zhang, P.; Li, Y.; Lin, M.; Liu, G.; Yao, M.; Liao, Q.; Xie, Z. A strategy for the targeted metabolomics analysis of 11 gut microbiota-host co-metabolites in rat serum, urine and feces by ultra high performance liquid chromatography-tandem mass spectrometry. *Journal of Chromatography A* **2016**, *1429*, 207-217.

36. Dong, F.; Zhang, L.; Hao, F.; Tang, H.; Wang, Y. Systemic responses of mice to dextran sulfate sodium-induced acute ulcerative colitis using 1H NMR spectroscopy. *Journal of proteome research* **2013**, *12*, 2958-2966.

37. Lin, H.M.; Barnett, M.P.G.; Roy, N.C.; Joyce, N.I.; Zhu, S.; Armstrong, K.; Helsby, N.A.; Ferguson, L.R.; Rowan, D.D. Metabolomic analysis identifies inflammatory and noninflammatory metabolic effects of genetic modification in a mouse model of Crohn?s disease. *Journal of proteome research* **2010**, *9*, 1965-1975.

38. Murdoch, T.B.; Fu, H.; MacFarlane, S.; Sydora, B.C.; Fedorak, R.N.; Slupsky, C.M. Urinary metabolic profiles of inflammatory bowel disease in interleukin-10 gene-deficient mice. *Analytical Chemistry* **2008**, *80*, 5524-5531.

39. Hong, Y.S.; Ahn, Y.T.; Park, J.C.; Lee, J.H.; Lee, H.; Huh, C.S.; Kim, D.H.; Ryu, D.H.; Hwang, G.S. 1H NMR-based metabonomic assessment of probiotic effects in a colitis mouse model. *Archives of pharmacal research* **2010**, *33*, 1091-1101.

40. Baur, P.; Martin, F.P.; Gruber, L.; Bosco, N.; Brahmbhatt, V.; Collino, S.; Guy, P.; Montoliu, I.; Rozman, J.; Klingenspor, M., et al. Metabolic phenotyping of the Crohn's disease-like IBD etiopathology in the TNFDELTAARE/WT mouse model. *Journal of proteome research* **2011**, *10*, 5523-5535.

41. Wang, R.; Gu, X.; Dai, W.; Ye, J.; Lu, F.; Chai, Y.; Fan, G.; Gonzalez, F.J.; Duan, G.; Qi, Y. A lipidomics investigation into the intervention of celastrol in experimental colitis. *Mol Biosyst* **2016**, *12*, 1436-1444, doi:10.1039/c5mb00864f.

42. Willenberg, I.; Ostermann, A.I.; Giovannini, S.; Kershaw, O.; Von Keutz, A.; Steinberg, P.; Schebb, N.H. Effect of acute and chronic DSS induced colitis on plasma eicosanoid and oxylipin levels in the rat. *Prostaglandins and Other Lipid Mediators* **2015**, *120*, 155-160.

43. Zhang, X.; Choi, F.F.; Zhou, Y.; Leung, F.P.; Tan, S.; Lin, S.; Xu, H.; Jia, W.; Sung, J.J.; Cai, Z., et al. Metabolite profiling of plasma and urine from rats with TNBS-induced acute colitis using UPLC-ESI-QTOF-MS-based metabonomics--a pilot study. *The FEBS journal* **2012**, *279*, 2322-2338, doi:10.1111/j.1742-4658.2012.08612.x.

44. Martin, F.P.J.; Lichti, P.; Bosco, N.; Brahmbhatt, V.; Oliveira, M.; Haller, D.; Benyacoub, J. Metabolic phenotyping of an adoptive transfer mouse model of experimental colitis and impact of dietary fish oil intake. *Journal of proteome research* **2015**, *14*, 1911-1919.

45. Liu, J.; Xiao, H.T.; Wang, H.S.; Mu, H.X.; Zhao, L.; Du, J.; Yang, D.; Wang, D.; Bian, Z.X.; Lin, S.H. Halofuginone reduces the inflammatory responses of DSS-induced colitis through metabolic reprogramming. *Mol Biosyst* **2016**, *12*, 2296-2303, doi:10.1039/c6mb00154h.

46. Gu, X.; Song, Y.; Chai, Y.; Lu, F.; Gonzalez, F.J.; Fan, G.; Qi, Y. GC-MS metabolomics on PPARalpha-dependent exacerbation of colitis. *Molecular bioSystems* **2015**, *11*, 1329-1337.

47. Robinson, A.M.; Gondalia, S.V.; Karpe, A.V.; Eri, R.; Beale, D.J.; Morrison, P.D.; Palombo, E.A.; Nurgali, K. Fecal microbiota and metabolome in a mouse model of spontaneous chronic colitis: Relevance to human inflammatory bowel disease. *Inflammatory bowel diseases* **2016**, *22*, 2767-2787.

48. Qu, C.; Yuan, Z.W.; Yu, X.T.; Huang, Y.F.; Yang, G.H.; Chen, J.N.; Lai, X.P.; Su, Z.R.; Zeng, H.F.; Xie, Y., et al. Patchouli alcohol ameliorates dextran sodium sulfate-induced experimental colitis and suppresses tryptophan catabolism. *Pharmacological research* **2017**, *121*, 70-82.

49. Otter, D.; Cao, M.; Lin, H.M.; Fraser, K.; Edmunds, S.; Lane, G.; Rowan, D. Identification of urinary biomarkers of colon inflammation in IL10-/- mice using Short-Column LCMS metabolomics. *Journal of biomedicine & biotechnology* **2011**, *2011*, 974701, doi:10.1155/2011/974701.

50. Kominsky, D.J.; Keely, S.; MacManus, C.F.; Glover, L.E.; Scully, M.; Collins, C.B.; Bowers, B.E.; Campbell, E.L.; Colgan, S.P. An endogenously anti-inflammatory role for methylation in mucosal inflammation identified through metabolite profiling. *J Immunol* **2011**, *186*, 6505-6514, doi:10.4049/jimmunol.1002805.

51. Vassilyadi, P.; Harding, S.V.; Nitschmann, E.; Wykes, L.J. Experimental colitis and malnutrition differentially affect the metabolism of glutathione and related sulfhydryl metabolites in different tissues. *European Journal of Nutrition* **2016**, *55*, 1769-1776.

52. Kohnke, T.; Gomolka, B.; Bilal, S.; Zhou, X.; Sun, Y.; Rothe, M.; Baumgart, D.C.; Weylandt, K.H. Acetylsalicylic Acid reduces the severity of dextran sodium sulfate-induced colitis and increases the formation of anti-inflammatory lipid mediators. *BioMed research international* **2013**, *2013*, 748160, doi:10.1155/2013/748160.

53. Zhang, W.; Liao, J.; Li, H.; Dong, H.; Bai, H.; Yang, A.; Hammock, B.D.; Yang, G.Y. Reduction of inflammatory bowel disease-induced tumor development in IL-10 knockout mice with soluble epoxide hydrolase gene deficiency. *Molecular Carcinogenesis* **2013**, *52*, 726-738.

54. Schicho, R.; Nazyrova, A.; Shaykhutdinov, R.; Duggan, G.; Vogel, H.J.; Storr, M. Quantitative metabolomic profiling of serum and urine in DSS-induced ulcerative colitis of mice by 1H NMR spectroscopy. *Journal of proteome research* **2010**, *9*, 6265-6273.

55. Martin, F.P.J.; Rezzi, S.; Montoliu, I.; Philippe, D.; Tornier, L.; Messlik, A.; Holzlwimmer, G.; Baur, P.; Quintanilla-Fend, L.; Loh, G., et al. Metabolic assessment of gradual development of moderate experimental colitis in IL-10 deficient mice. *Journal of proteome research* **2009**, *8*, 2376-2387.

56. Qi, Y.; Jiang, C.; Tanaka, N.; Krausz, K.W.; Brocker, C.N.; Fang, Z.Z.; Bredell, B.X.; Shah, Y.M.; Gonzalez, F.J. PPARalpha-dependent exacerbation of experimental colitis by the hypolipidemic drug fenofibrate. *American Journal of Physiology - Gastrointestinal and Liver Physiology* **2014**, *307*, G564-G573.

57. Jacobs, J.P.; Lin, L.; Goudarzi, M.; Ruegger, P.; McGovern, D.P.B.; Fornace, A.J.; Borneman, J.; Xia, L.; Braun, J. Microbial, metabolomic, and immunologic dynamics in a relapsing genetic mouse model of colitis induced by T-synthase deficiency. *Gut microbes* **2017**, *8*, 1-16.

58. Lu, K.; Knutson, C.G.; Wishnok, J.S.; Fox, J.G.; Tannenbaum, S.R. Serum metabolomics in a helicobacter hepaticus mouse model of inflammatory bowel disease reveal important changes in the microbiome, serum peptides, and intermediary metabolism. *Journal of proteome research* **2012**, *11*, 4916-4926.
