# Supplementary material for: Translational Potential of Metabolomics on Animal Models of Inflammatory Bowel Disease—A Systematic Critical Review
Source: Int J Mol Sci. 2020 May 29;21(11):3856. doi: 10.3390/ijms21113856 (PMC7312423; doi:10.3390/ijms21113856)
Supplement: Supplementary file 1 [file ijms-21-03856-s001.zip › Supplementary Table S5_resubmission_proofread.docx]

**Supplementary Table S5: Metabolites significantly increased in IBD vs healthy controls in humans**

| **Metabolites ↑ in IBD** | **Disease** | **Activity** | **Sample type** | **Age group** | **Platform** | **Reference** |
| --- | --- | --- | --- | --- | --- | --- |
| - | CD | AC | Feces | A, O | ^1^H-NMR | [1] |
| - | CD | IA | Feces | A, O | ^1^H-NMR | [1] |
| - | CD | IA | Urine | A, O | ^1^H-NMR | [2] |
| - | UC | All | Urine | Y, A, O | ^1^H-NMR | [3] |
| - | CD | IA | PBMC Macrophages | A, O | ESI-MS | [4] |
| - | CD | All | Ileum | A, O | ESI-MS | [4] |
| - | CD | IA | Feces | A, O | GC-MS | [5] |
| - | CD | IA | Urine | A, O | GC-MS | [6] |
| - | CD | IA | Feces | A | GC-MS | [7] |
| - | CD | IA | Plasma | A | GC-MS | [8] |
| - | UC | AC | Feces | A, O | GC-MS | [5] |
| - | UC | AC | Serum | A, O | GC-MS | [9] |
| - | UC | IA | Plasma | A | GC-MS | [8] |
| - | UC | All | Feces | A | GC-MS | [10] |
| - | UC | All | Rectum tissue | Y, A, O | GC-MS | [11] |
| - | UC | All | Plasma | A | GC-MS | [8] |
| - | IBD | IA | Feces | A, O | GC-tof-MS | [12] |
| - | CD | IA | PBMC Macrophages | A, O | HPLC-MS | [4] |
| - | IBD | All | Urine | A, O | NMR | [13] |
| - | CD | IA | Colonic mucosa | Unknown | Proton MRS | [14] |
| - | UC | All | Breath | A, O | SIFT-MS | [15] |
| - | UC | IA | Feces | Unknown | UPLC/tof-MS | [16] |
| (Z)/4/Hydroxyphenyl-acetaldehyde-oxime | CD (CCD) | IA | Feces | Y, A, O | FT-ICR-MS | [17] |
| 1,6-Anhydroglucose | UC | AC | Serum | Y, A, O | GC-MS | [18] |
| 1,6-Anhydroglucose | UC | IA | Serum | Y, A, O | GC-MS | [18] |
| 1,6-Anhydroglucose | UC | All | Serum | Y, A, O | GC-MS | [18] |
| 13-Sophorosyloxydocosanoic acid | CD | All | Plasma | A | LC-MS/MS | [8] |
| 1-Butanol, 3-methyl propanoic acid | CD (sb) | AC | Feces | A, O | GC-MS | [5] |
| 1-Butanol-3-methyl | UC | IA | Feces | A, O | GC-MS | [5] |
| 1-Butoxy-2-propanol | CD | AC | Breath | A | GC-tof-MS | [19] |
| 1-Decene | IBD | AC | Breath | Y, A | SIFT-MS | [20] |
| 1-Decene | IBD | AC | Breath | Y, A | SIFT-MS | [20] |
| 1-Ethyl-3-methylbenzene | CD | IA | Feces | A, O | GC-tof-MS | [12] |
| 1-Ethyl-3-methylbenzene | CD | All | Feces | A, O | GC-tof-MS | [12] |
| 1-Ethyl-3-methyl-benzene | CD | All | Feces | A | GC-MS | [7] |
| 1-Heptene | IBD | AC | Breath | Y, A | SIFT-MS | [20] |
| 1-Hexadecanyl-2-((2/'alpha-glucosyl)-beta-glucosyl)-3-beta-xylosyl-sn-glycerol | CD | All | Plasma | A | LC-MS/MS | [8] |
| 1-Nitroheptane | CD (sb) | AC | Feces | A, O | GC-MS | [5] |
| 1-Octen -3-ol | CD (sb) | AC | Feces | A, O | GC-MS | [5] |
| 1-Octen-3-ol | CD | AC | Feces | A, O | GC-MS | [5] |
| 1-Octene | IBD | AC | Breath | Y, A | SIFT-MS | [20] |
| 1-Octene | IBD | AC | Breath | Y, A | SIFT-MS | [20] |
| 1-Pentanol | CD (sb) | AC | Feces | A, O | GC-MS | [5] |
| 2-(3-Carboxy-3-aminopropyl)-L-histidine (Diphthamide) | CD | All | Plasma | A | LC-MS/MS | [8] |
| 2,2,4-Trimethylpentane | CD | AC | Breath | A | GC-tof-MS | [19] |
| 2,4-Dithiapentane | UC | All | Feces | A, O | GC-tof-MS | [12] |
| 2,8-Dihydroxyquinoline-beta-D-glucuronide | UC | All | Plasma | A | LC-MS/MS | [8] |
| 24S-Hydroxycholesterol | CD | All | Serum | A | LC-ESI-MS/MS | [21] |
| 25-Hydroxycholesterol | CD | All | Serum | A | LC-ESI-MS/MS | [21] |
| 27-Hydroxycholesterol | CD | All | Serum | A | LC-ESI-MS/MS | [21] |
| 2-Carboxy-2,3-dihydro-5,6-dihydroxyindole/dopaquinone | CD (CCD) | IA | Feces | Y, A, O | FT-ICR-MS | [17] |
| 2-Dehydro-D-gluconate_1 | UC | AC | Serum | Y, A, O | GC-MS | [18] |
| 2-Dehydro-D-gluconate_1 | UC | All | Serum | Y, A, O | GC-MS | [18] |
| 2-Hydroxybutyrate | CD | AC | Plasma | A, O | ^1^H-NMR | [22] |
| 2-Hydroxybutyrate | UC | AC | Serum | A, O | ^1^H-NMR | [22] |
| 2-Hydroxybutyrate | UC | AC | Plasma | A, O | ^1^H-NMR | [22] |
| 2-Methyl propanal | CD | All | Feces | A, O | GC-tof-MS | [12] |
| 2-Methyl-2-propenyl benzene | UC | All | Feces | A, O | GC-tof-MS | [12] |
| 2-Methyl-2-propenyl-benzene | CD | All | Feces | A | GC-MS | [7] |
| 2-Methylpropanal | CD | All | Feces | A | GC-MS | [7] |
| 2-Oxoisocaproate | UC | AC | Urine | A, O | ^1^H-NMR | [22] |
| 2-Oxoisocaproate | UC | All | Urine | Y | ^1^H-NMR | [23] |
| 2-Oxoisocaproate | UC | All | Urine | Y | ^1^H-NMR | [23] |
| 2-Piperidinone | CD | AC | Feces | A, O | GC-MS | [5] |
| 2-Propanol | CD | Unknown | Breath | A | SIFT-MS | [24] |
| 2-Undecanone | CD (sb) | AC | Feces | A, O | GC-MS | [5] |
| 3-(2,4-Cyclopentadien-1-ylidene)-5alpha-androstan-17beta-ol | CD | All | Plasma | A | LC-MS/MS | [8] |
| 3-(4-Hydroxy-phenyl)propionic acid/3-(4-hydroxyphenyl)lactate | CD (ICD) | IA | Feces | Y, A, O | FT-ICR-MS | [17] |
| 3-Hydroxybutyrate | UC | AC | Serum | A, O | ^1^H-NMR | [25] |
| 3-Hydroxybutyrate | IBD | AC | Serum | A, O | ^1^H-NMR | [26] |
| 3-Hydroxyisobutyrate | UC | All | Urine | Y | ^1^H-NMR | [23] |
| 3-Hydroxy-OPC8-CoA | UC | All | Plasma | A | LC-MS/MS | [8] |
| 3-Methyl-2-oxovalerate | CD | AC | Plasma | A, O | ^1^H-NMR | [22] |
| 3-Methyl-2-oxovalerate | UC | AC | Serum | A, O | ^1^H-NMR | [22] |
| 3-Methyl-2-oxovalerate | UC | AC | Plasma | A, O | ^1^H-NMR | [22] |
| 3-Methyl-2-oxovalerate | UC | All | Urine | Y | ^1^H-NMR | [23] |
| 3-Methyl-2-oxovalerate | UC | All | Urine | Y | ^1^H-NMR | [23] |
| 3-Methylhexane | IBD | AC | Breath | Y, A | SIFT-MS | [20] |
| 3-Methylhexane | IBD | AC | Breath | Y, A | SIFT-MS | [20] |
| 3-O-L-Rhamnosyl-3-hydroxydecanoyl-3-hydroxydecanoic acid | CD | All | Plasma | A | LC-MS/MS | [8] |
| 3-Sulfodeoxycholic acid | CD | IA | Feces | Unknown | UPLC/tof-MS | [16] |
| 4-Aminobutyric acid | UC | AC | Serum | Y, A, O | GC-MS | [18] |
| 4-Aminobutyric acid | UC | All | Serum | Y, A, O | GC-MS | [18] |
| 4-Hydroxyphenylacetate | CD | AC | Urine | A, O | ^1^H-NMR | [22] |
| 4-Hydroxyphenylacetate | CD | All | Urine | Y | ^1^H-NMR | [23] |
| 4-Hydroxyphenylacetate | UC | All | Urine | Y | ^1^H-NMR | [23] |
| 4-Hydroxyphenylacetate | UC | All | Urine | Y | ^1^H-NMR | [23] |
| 4-Hydroxyphenylacetate | UC | All | Urine | Y | ^1^H-NMR | [23] |
| 4-Hydroxyphenylacetate | UC | All | Urine | Y | ^1^H-NMR | [23] |
| 4-Hydroxyphenylacetylglycine | CD (CCD) | IA | Feces | Y, A, O | FT-ICR-MS | [17] |
| 4-Hydroxyphenylpyruvate | UC | All | Urine | Y | ^1^H-NMR | [23] |
| 4-Hydroxyphenylpyruvate | UC | All | Urine | Y | ^1^H-NMR | [23] |
| 4-Methylbenzoic acid | UC | AC | Serum | Y, A, O | GC-MS | [18] |
| 4-Methylbenzoic acid | UC | IA | Serum | Y, A, O | GC-MS | [18] |
| 4-Methylbenzoic acid | UC | All | Serum | Y, A, O | GC-MS | [18] |
| 4β-Hydroxycholesterol | CD | All | Serum | A | LC-ESI-MS/MS | [21] |
| 5-Methyl-2-(1-methylethyl)-cyclohexanol | CD | AC | Feces | A | GC-MS | [7] |
| 5-Methyl-2-(1-methylethyl)-cyclohexanol | CD | All | Feces | A | GC-MS | [7] |
| 5-Methyl-2-furancarboxaldehyde | CD | All | Feces | A | GC-MS | [7] |
| 6-Methyl, 2-heptanone | CD | AC | Feces | A, O | GC-MS | [5] |
| 7-Ketodeoxycholic acid | CD | IA | Feces | Unknown | UPLC/tof-MS | [16] |
| 7α-Hydroxy-4-cholesten-3-one | CD | All | Serum | A | LC-ESI-MS/MS | [21] |
| 7α-Hydroxy-4-cholesten-3-one | CD | All | Serum | A | LC-ESI-MS/MS | [21] |
| Acetate | CD | AC | Plasma | A, O | ^1^H-NMR | [22] |
| Acetate | UC | AC | Urine | A, O | ^1^H-NMR | [22] |
| Acetoacetate | IBD | AC | Serum | A, O | ^1^H-NMR | [26] |
| Acetoacetate | IBD | IA | Urine | A, O | ^1^H-NMR | [26] |
| Acetone | CD | All | Feces | A | GC-MS | [7] |
| Acetylated compounds, N- | IBD | AC | Serum | A, O | ^1^H-NMR | [26] |
| Acetylcadaverine, N- | CD | IA | Feces | Unknown | UPLC/tof-MS | [16] |
| Acetylcarnitine, O- | CD | AC | Urine | A, O | ^1^H-NMR | [22] |
| Acetylcarnitine, O- | UC | AC | Urine | A, O | ^1^H-NMR | [22] |
| Acetyl-L-aspartic acid_2, N- | UC | AC | Serum | Y, A, O | GC-MS | [18] |
| Acetyl-L-aspartic acid_2, N- | UC | IA | Serum | Y, A, O | GC-MS | [18] |
| Acetyl-L-aspartic acid_2, N- | UC | All | Serum | Y, A, O | GC-MS | [18] |
| Acetyl-L-glutamate_1, N- | UC | AC | Serum | Y, A, O | GC-MS | [18] |
| Acetyl-L-glutamate_1, N- | UC | All | Serum | Y, A, O | GC-MS | [18] |
| Acetyl-L-ornithine_2, N-α- | UC | AC | Serum | Y, A, O | GC-MS | [18] |
| Acetyl-L-ornithine_2, N-α- | UC | IA | Serum | Y, A, O | GC-MS | [18] |
| Acetyl-L-ornithine_2, N-α- | UC | All | Serum | Y, A, O | GC-MS | [18] |
| Acrylonitrile | CD | Unknown | Breath | A | SIFT-MS | [24] |
| Acrylonitrile | UC | Unknown | Breath | A | SIFT-MS | [24] |
| Acyl-carnitine | CD | All | Urine | Y | ^1^H-NMR | [23] |
| Adipic acid | UC | AC | Serum | Y, A, O | GC-MS | [18] |
| Adipic acid | UC | All | Serum | Y, A, O | GC-MS | [18] |
| Alanine | CD | AC | Feces | A, O | ^1^H-NMR | [1] |
| Alanine | CD | Unknown | Feces | Y, A, O | ^1^H-NMR | [27] |
| Alanine | UC | AC | Feces | A, O | ^1^H-NMR | [1] |
| Alanine | UC | AC | Feces | A, O | ^1^H-NMR | [1] |
| Alanine | CD | All | Serum | Y, A, O | GC-MS | [11] |
| Allantoin | UC | AC | Urine | A, O | ^1^H-NMR | [22] |
| Angiotensin IV | CD | All | Plasma | A | LC-MS/MS | [8] |
| Arachidonic acid | CD (ICD) | IA | Feces | Y, A, O | FT-ICR-MS | [17] |
| Arginine/Leucine/Lysine | UC | AC | Colonic mucosa | Unknown | Proton MRS | [14] |
| Arginine | CD | AC | Serum | A, O | ^1^H-NMR | [22] |
| Arginine | CD | AC | Plasma | A, O | ^1^H-NMR | [22] |
| Arginine | UC | AC | Urine | A, O | ^1^H-NMR | [22] |
| Aspartic acid | CD | All | Serum | Y, A, O | GC-MS | [11] |
| Aspartic acid | UC | All | Serum | Y, A, O | GC-MS | [11] |
| Benzaldehyde | UC | IA | Feces | A, O | GC-MS | [5] |
| Benzene acetaldehyde | CD | All | Feces | A, O | GC-tof-MS | [12] |
| Benzene acetaldehyde | UC | IA | Feces | A, O | GC-tof-MS | [12] |
| Benzene acetaldehyde | UC | All | Feces | A, O | GC-tof-MS | [12] |
| Benzene acetaldehyde | IBD | All | Feces | A, O | GC-tof-MS | [12] |
| Benzeneacetaldehyde | CD | All | Feces | A | GC-MS | [7] |
| Benzeneacetaldehyde | CD | IA | Feces | A, O | GC-tof-MS | [12] |
| Branched saturated C12 compound (RT = 16.6 min) | CD | AC | Breath | A | GC-tof-MS | [19] |
| Butanal | CD | All | Breath | A, O | SIFT-MS | [15] |
| Butanoic acid, 2-methyl-methyl ester | CD | AC | Feces | A, O | GC-MS | [5] |
| C16:0-Platelet activating factor | UC | AC | Colonic mucsa | A, O | GC-MS | [28] |
| Cadaverine | UC | All | Feces | A, O | ^1^H-NMR | [29] |
| Carbon disulfide | CD | All | Feces | A | GC-MS | [7] |
| Carbon disulfide | CD | All | Feces | A, O | GC-tof-MS | [12] |
| Carbon disulfide | CD | Unknown | Breath | A | SIFT-MS | [24] |
| Carbon disulfide | UC | Unknown | Breath | A | SIFT-MS | [24] |
| Carnitine | CD | AC | Urine | A, O | ^1^H-NMR | [22] |
| Carnitine | UC | AC | Urine | A, O | ^1^H-NMR | [22] |
| Catechol | UC | AC | Serum | Y, A, O | GC-MS | [18] |
| Catechol | UC | IA | Serum | Y, A, O | GC-MS | [18] |
| Catechol | UC | All | Serum | Y, A, O | GC-MS | [18] |
| Chenodeoxycholic acid sulfate | CD | IA | Feces | Unknown | UPLC/tof-MS | [16] |
| Chenodeoxyglycocholate/Glycochenodeoxycholate | CD (ICD) | IA | Feces | Y, A, O | FT-ICR-MS | [17] |
| Cholic acid | CD | IA | Feces | Unknown | UPLC/tof-MS | [16] |
| Choline | UC | All | Feces | A, O | ^1^H-NMR | [29] |
| Compound RT311 | UC | IA | Feces | A, O | GC-MS | [5] |
| Creatine | CD | AC | Plasma | A, O | ^1^H-NMR | [22] |
| Creatine | UC | AC | Serum | A, O | ^1^H-NMR | [22] |
| Creatine | UC | AC | Plasma | A, O | ^1^H-NMR | [22] |
| Cystamine | UC | AC | Serum | Y, A, O | GC-MS | [18] |
| Cystamine | UC | IA | Serum | Y, A, O | GC-MS | [18] |
| Cystamine | UC | All | Serum | Y, A, O | GC-MS | [18] |
| Decane | CD | AC | Feces | A, O | GC-MS | [5] |
| Dihydroorotate | UC | AC | Serum | Y, A, O | GC-MS | [18] |
| Dihydroorotate | UC | IA | Serum | Y, A, O | GC-MS | [18] |
| Dihydroorotate | UC | All | Serum | Y, A, O | GC-MS | [18] |
| Dimethyl sulfide | CD | All | Breath | A, O | SIFT-MS | [15] |
| Dimethyl sulfide | CD | Unknown | Breath | A | SIFT-MS | [24] |
| Dimethylamine | IBD | IA | Serum | A, O | ^1^H-NMR | [26] |
| Dimethylsulfide | CD (sb) | AC | Feces | A, O | GC-MS | [5] |
| Dopa | UC | AC | Serum | Y, A, O | GC-MS | [18] |
| Dopa | UC | IA | Serum | Y, A, O | GC-MS | [18] |
| Dopa | UC | All | Serum | Y, A, O | GC-MS | [18] |
| Ethanol | CD | Unknown | Breath | A | SIFT-MS | [24] |
| Ethylmalonic acid | UC | AC | Serum | Y, A, O | GC-MS | [18] |
| Ethylmalonic acid | UC | IA | Serum | Y, A, O | GC-MS | [18] |
| Ethylmalonic acid | UC | All | Serum | Y, A, O | GC-MS | [18] |
| Formate | CD | All | Urine | Y, A, O | ^1^H-NMR | [3] |
| Formate | CD (CCD) | All | Urine | Y, A, O | ^1^H-NMR | [3] |
| Formate | UC | AC | Serum | A, O | ^1^H-NMR | [22] |
| Formate | UC | AC | Plasma | A, O | ^1^H-NMR | [22] |
| Formate | UC | All | Urine | Y | ^1^H-NMR | [23] |
| Fructose_1 | UC | IA | Serum | Y, A, O | GC-MS | [18] |
| Fucα1-2Galα1-3Galβ1-4Glcβ-Cer(d18:1/16:0) | CD | All | Plasma | A | LC-MS/MS | [8] |
| Fucα1-2Galα1-3Galβ1-4Glcβ-Cer(d18:1/24:1(15Z)) | CD | All | Plasma | A | LC-MS/MS | [8] |
| Fumaric acid | CD | All | Serum | Y, A, O | GC-MS | [11] |
| Fumaric acid | UC | All | Serum | Y, A, O | GC-MS | [11] |
| Furan | CD | All | Feces | A | GC-MS | [7] |
| Galactose | CD | AC | Urine | A, O | ^1^H-NMR | [22] |
| Galactose_2 | UC | AC | Serum | Y, A, O | GC-MS | [18] |
| Galactose_2 | UC | All | Serum | Y, A, O | GC-MS | [18] |
| Galacturonic acid_1 | UC | AC | Serum | Y, A, O | GC-MS | [18] |
| Galacturonic acid_1 | UC | IA | Serum | Y, A, O | GC-MS | [18] |
| Galacturonic acid_1 | UC | All | Serum | Y, A, O | GC-MS | [18] |
| Galα1-3(Fucα1-2)Galβ1-4Glcβ-Cer(D18:1/16:0) | CD | All | Plasma | A | LC-MS/MS | [8] |
| Galα1-3(Fucα1-2)Galβ1-4Glcβ-Cer(d18:1/18:0) | CD | All | Plasma | A | LC-MS/MS | [8] |
| Galα1-3(Fucα1-2)Galβ1-4Glcβ-Cer(d18:1/20:0) | CD | All | Plasma | A | LC-MS/MS | [8] |
| Galα1-3(Fucα1-2)Galβ1-4Glcβ-Cer(d18:1/24:1(15Z)) | CD | All | Plasma | A | LC-MS/MS | [8] |
| Galα1-3(Fucα1-2)Galβ1-4Glcβ-Cer(d18:1/26:1(17Z)) | CD | All | Plasma | A | LC-MS/MS | [8] |
| Ganglioside GM3 (d18:0/23:0) | CD | All | Plasma | A | LC-MS/MS | [8] |
| Ganglioside GM3 (d18:0/24:0) | CD | All | Plasma | A | LC-MS/MS | [8] |
| Ganglioside GM3 (d18:0/25:0) | CD | All | Plasma | A | LC-MS/MS | [8] |
| Glucose | UC | AC | Serum | A, O | ^1^H-NMR | [22] |
| Glucose | UC | All | Feces | A, O | ^1^H-NMR | [29] |
| Glucose, α- | UC | AC | Serum | A, O | ^1^H-NMR | [25] |
| Glucose, α- | IBD | AC | Colonic mucosa | A | ^1^H-NMR | [30] |
| Glucose, α- | CD | AC | Colonic mucosa | Unknown | Proton MRS | [14] |
| Glucose, α- | UC | AC | Colonic mucosa | Unknown | Proton MRS | [14] |
| Glucose, α- | UC | IA | Colonic mucosa | Unknown | Proton MRS | [14] |
| Glucose, β- | UC | AC | Serum | A, O | ^1^H-NMR | [25] |
| Glucose_1 | UC | AC | Serum | Y, A, O | GC-MS | [18] |
| Glucose_1 | UC | IA | Serum | Y, A, O | GC-MS | [18] |
| Glucose_1 | UC | All | Serum | Y, A, O | GC-MS | [18] |
| Glutamate | UC | Unknown | Feces | Y, A, O | ^1^H-NMR | [27] |
| Glutamic acid, β- | UC | AC | Serum | Y, A, O | GC-MS | [18] |
| Glutamic acid, β- | UC | IA | Serum | Y, A, O | GC-MS | [18] |
| Glutamic acid, β- | UC | All | Serum | Y, A, O | GC-MS | [18] |
| Glycerol | CD | AC | Plasma | A, O | ^1^H-NMR | [22] |
| Glycerol | UC | AC | Serum | Y, A, O | GC-MS | [18] |
| Glycine | CD | AC | Feces | A, O | ^1^H-NMR | [1] |
| Glycine | CD | AC | Serum | A, O | ^1^H-NMR | [22] |
| Glycine | CD | IA | Feces | A, O | ^1^H-NMR | [1] |
| Glycine | CD | All | Urine | Y | ^1^H-NMR | [23] |
| Glycine | UC | All | Urine | Y | ^1^H-NMR | [23] |
| Glycine | IBD | AC | Serum | A, O | ^1^H-NMR | [26] |
| Glycine | CD | All | Serum | Y, A, O | GC-MS | [11] |
| Glycine | UC | All | Serum | Y, A, O | GC-MS | [11] |
| Glycylproline | UC | AC | Urine | A, O | ^1^H-NMR | [22] |
| Heptadecane | CD | AC | Breath | A | GC-tof-MS | [19] |
| Heptadecane | CD | IA | Breath | A | GC-tof-MS | [19] |
| Heptanal | CD | AC | Feces | A, O | GC-MS | [5] |
| Heptanoic acid | CD (sb) | AC | Feces | A, O | GC-MS | [5] |
| Hexanal | CD | All | Feces | A | GC-MS | [7] |
| Homocysteine | UC | AC | Serum | Y, A, O | GC-MS | [18] |
| Homocysteine | UC | IA | Serum | Y, A, O | GC-MS | [18] |
| Homocysteine | UC | All | Serum | Y, A, O | GC-MS | [18] |
| Homocysteine_1 | UC | AC | Serum | Y, A, O | GC-MS | [18] |
| Homocysteine_1 | UC | IA | Serum | Y, A, O | GC-MS | [18] |
| Homocysteine_1 | UC | All | Serum | Y, A, O | GC-MS | [18] |
| Hydrogen sulfide | CD | All | Feces | A | GC-MS | [7] |
| Hydroxyaspartic acid, threo-β- | UC | AC | Serum | Y, A, O | GC-MS | [18] |
| Hydroxyaspartic acid, threo-β- | UC | All | Serum | Y, A, O | GC-MS | [18] |
| Hydroxybenzoic acid, p- | UC | AC | Serum | Y, A, O | GC-MS | [18] |
| Hydroxybenzoic acid, p- | UC | All | Serum | Y, A, O | GC-MS | [18] |
| Hydroxybutyrate | UC | AC | Serum | Y, A, O | GC-MS | [18] |
| Inositol, myo- | CD | AC | Urine | A, O | ^1^H-NMR | [22] |
| Isoleucine | CD | AC | Feces | A, O | ^1^H-NMR | [1] |
| Isoleucine | CD | AC | Serum | A | ^1^H-NMR | [31] |
| Isoleucine | CD | AC | Serum | A, O | ^1^H-NMR | [22] |
| Isoleucine | CD | AC | Plasma | A, O | ^1^H-NMR | [22] |
| Isoleucine | CD | Unknown | Feces | Y, A, O | ^1^H-NMR | [27] |
| Isoleucine | UC | AC | Feces | A, O | ^1^H-NMR | [1] |
| Isoleucine | UC | AC | Feces | A, O | ^1^H-NMR | [1] |
| Isoleucine | UC | AC | Serum | A, O | ^1^H-NMR | [22] |
| Isoleucine | UC | AC | Plasma | A, O | ^1^H-NMR | [22] |
| Isoleucine | IBD | AC | Serum | A, O | ^1^H-NMR | [26] |
| Isoprene | CD | Unknown | Breath | A | SIFT-MS | [24] |
| Isoprene | IBD | AC | Breath | Y, A | SIFT-MS | [20] |
| Isoprostaglandin F2α type III | CD | AC | Urine | A, O | GC-MS | [6] |
| KCNα2-3Galβ1-4Glcβ-Cer(d18:1/20:0) | CD | All | Plasma | A | LC-MS/MS | [8] |
| KDNα2-3Galβ1-4Glcβ-Cer(18:1/16:0) | CD | All | Plasma | A | LC-MS/MS | [8] |
| KDNα2-3Galβ1-4Glcβ-Cer(d18:1/20:0) | CD | All | Plasma | A | LC-MS/MS | [8] |
| KDNα2-3Galβ1-4Glcβ-Cer(d18:1/24:1(15Z)) | CD | All | Plasma | A | LC-MS/MS | [8] |
| Kynurenine | UC | AC | Serum | Y, A, O | GC-MS | [18] |
| Kynurenine | UC | IA | Serum | Y, A, O | GC-MS | [18] |
| Kynurenine | UC | All | Serum | Y, A, O | GC-MS | [18] |
| Lactate | CD | AC | Plasma | A, O | ^1^H-NMR | [22] |
| Lactate | CD | AC | Urine | A, O | ^1^H-NMR | [22] |
| Lactate | UC | AC | Feces | A, O | ^1^H-NMR | [1] |
| Lactate | UC | AC | Feces | A, O | ^1^H-NMR | [1] |
| Lactate | UC | AC | Urine | A, O | ^1^H-NMR | [22] |
| Lactate | UC | All | Urine | Y | ^1^H-NMR | [23] |
| Lactate | UC | All | Urine | Y | ^1^H-NMR | [23] |
| Lactate | IBD | AC | Serum | A, O | ^1^H-NMR | [26] |
| Lactic acid | UC | AC | Serum | Y, A, O | GC-MS | [18] |
| Lactic acid | UC | IA | Serum | Y, A, O | GC-MS | [18] |
| Lactic acid | UC | All | Serum | Y, A, O | GC-MS | [18] |
| Lactose | CD | AC | Urine | A, O | ^1^H-NMR | [22] |
| Lathosterol | CD | All | Serum | A | LC-ESI-MS/MS | [21] |
| Lathosterol | CD | All | Serum | A | LC-ESI-MS/MS | [21] |
| Leucine | CD | AC | Feces | A, O | ^1^H-NMR | [1] |
| Leucine | CD | IA | Feces | A, O | ^1^H-NMR | [1] |
| Leucine | CD | Unknown | Feces | Y, A, O | ^1^H-NMR | [27] |
| Leucine | UC | AC | Feces | A, O | ^1^H-NMR | [1] |
| Leucine | UC | AC | Feces | A, O | ^1^H-NMR | [1] |
| Leucine | IBD | AC | Serum | A, O | ^1^H-NMR | [26] |
| Linoleic acid | CD (ICD) | IA | Feces | Y, A, O | FT-ICR-MS | [17] |
| Lysine | CD | AC | Feces | A, O | ^1^H-NMR | [1] |
| Lysine | CD | AC | Plasma | A, O | ^1^H-NMR | [22] |
| Lysine | CD | Unknown | Feces | Y, A, O | ^1^H-NMR | [27] |
| Lysine | UC | AC | Feces | A, O | ^1^H-NMR | [1] |
| Lysine | UC | AC | Feces | A, O | ^1^H-NMR | [1] |
| Lysine | UC | AC | Serum | A, O | ^1^H-NMR | [22] |
| Lysine | UC | AC | Plasma | A, O | ^1^H-NMR | [22] |
| Lysine | UC | Unknown | Feces | Y, A, O | ^1^H-NMR | [27] |
| Maleamic acid | UC | AC | Serum | Y, A, O | GC-MS | [18] |
| Maleic acid | UC | AC | Serum | Y, A, O | GC-MS | [18] |
| Maleic acid | UC | IA | Serum | Y, A, O | GC-MS | [18] |
| Maleic acid | UC | All | Serum | Y, A, O | GC-MS | [18] |
| Malic acid | CD | All | Serum | Y, A, O | GC-MS | [11] |
| Malonic acid | UC | AC | Serum | Y, A, O | GC-MS | [18] |
| Malonic acid | UC | IA | Serum | Y, A, O | GC-MS | [18] |
| Malonic acid | UC | All | Serum | Y, A, O | GC-MS | [18] |
| Maltose | CD | AC | Urine | A, O | ^1^H-NMR | [22] |
| Mannitol | CD | All | Urine | Y | ^1^H-NMR | [23] |
| Mannitol | UC | AC | Urine | A, O | ^1^H-NMR | [22] |
| Mannose | CD | AC | Serum | A, O | ^1^H-NMR | [22] |
| Mannose | CD | AC | Plasma | A, O | ^1^H-NMR | [22] |
| Mannose | UC | AC | Serum | A, O | ^1^H-NMR | [22] |
| Mannose | UC | AC | Plasma | A, O | ^1^H-NMR | [22] |
| Mercaptoacetone | UC | IA | Feces | A, O | GC-MS | [5] |
| Methanol | CD | AC | Serum | A, O | ^1^H-NMR | [22] |
| Methanol | UC | AC | Serum | A, O | ^1^H-NMR | [22] |
| Methionine | UC | AC | Serum | A, O | ^1^H-NMR | [22] |
| Methionine | CD | All | Serum | Y, A, O | GC-MS | [11] |
| Methyl 13-sophorosyloxydocosanoate | CD | All | Plasma | A | LC-MS/MS | [8] |
| MGDG (20:5(5Z,8Z,11Z,14Z,17Z)/18:4(6Z,9Z,12Z,15Z)) | CD | All | Plasma | A | LC-MS/MS | [8] |
| NeuAcα2-3Galβ-Cer(d18:1/20:0) | CD | All | Plasma | A | LC-MS/MS | [8] |
| Nonanal | UC | IA | Feces | A, O | GC-MS | [5] |
| Nonanal | CD | All | Breath | A, O | SIFT-MS | [15] |
| O-(N-acetyl-a-neuraminosyl)…* | CD | All | Plasma | A | LC-MS/MS | [8] |
| Octadecatrienoic acid | CD (ICD) | IA | Feces | Y, A, O | FT-ICR-MS | [17] |
| Oleic acid | CD (ICD) | IA | Feces | Y, A, O | FT-ICR-MS | [17] |
| Palmitic acid | CD (ICD) | IA | Feces | Y, A, O | FT-ICR-MS | [17] |
| Pantothenate | UC | AC | Serum | Y, A, O | GC-MS | [18] |
| Pantothenate | UC | IA | Serum | Y, A, O | GC-MS | [18] |
| Pantothenate | UC | All | Serum | Y, A, O | GC-MS | [18] |
| PC (18:2(9Z,12Z)/2:0)[U] | CD | All | Plasma | A | LC-MS/MS | [8] |
| Phenethylamine | CD | IA | Feces | Unknown | UPLC/tof-MS | [16] |
| Phenol | CD | All | Feces | A | GC-MS | [7] |
| Phenol | CD | IA | Feces | A, O | GC-tof-MS | [12] |
| Phenol | CD | All | Feces | A, O | GC-tof-MS | [12] |
| Phenylacetylglutamine | CD | All | Urine | Y | ^1^H-NMR | [23] |
| Phenylacetylglutamine | CD | All | Urine | Y | ^1^H-NMR | [23] |
| Phenylacetylglutamine | CD | All | Urine | Y | ^1^H-NMR | [23] |
| Phenylacetylglutamine | CD | All | Urine | Y | ^1^H-NMR | [23] |
| Phenylacetylglutamine | UC | All | Urine | Y | ^1^H-NMR | [23] |
| Phenylacetylglutamine | UC | All | Urine | Y | ^1^H-NMR | [23] |
| Phenylacetylglutamine | UC | All | Urine | Y | ^1^H-NMR | [23] |
| Phenylacetylglutamine | UC | All | Urine | Y | ^1^H-NMR | [23] |
| Phenylacetylglycine | UC | All | Urine | A | ^1^H-NMR | [32] |
| Phenylalanine | CD | AC | Feces | A, O | ^1^H-NMR | [1] |
| Phenylalanine | UC | AC | Serum | A, O | ^1^H-NMR | [25] |
| Phenylalanine | IBD | AC | Serum | A, O | ^1^H-NMR | [26] |
| Prolinamide | UC | AC | Serum | Y, A, O | GC-MS | [18] |
| Prolinamide | UC | IA | Serum | Y, A, O | GC-MS | [18] |
| Prolinamide | UC | All | Serum | Y, A, O | GC-MS | [18] |
| Proline | CD | AC | Serum | A, O | ^1^H-NMR | [22] |
| Proline | CD | All | Serum | Y, A, O | GC-MS | [11] |
| Propanoic acid, propyl ester | CD | AC | Feces | A, O | GC-MS | [5] |
| Prostaglandin E2 | CD | Unknown | Urine | A | LC-MS | [33] |
| PS (18:0/22.5(7Z,10Z,13Z,16Z,19Z)) | CD | All | Plasma | A | LC-MS/MS | [8] |
| Pyruvate | UC | AC | Serum | A, O | ^1^H-NMR | [22] |
| Pyruvate | UC | AC | Urine | A, O | ^1^H-NMR | [22] |
| Quinolinic acid | CD | All | Plasma | A | GC-MS | [8] |
| Sphingomyelin (d18:0/18:0) | CD | All | Plasma | A | LC-MS/MS | [8] |
| Sporidesmin | UC | All | Plasma | A | LC-MS/MS | [8] |
| Stearic acid | CD (ICD) | IA | Feces | Y, A, O | FT-ICR-MS | [17] |
| Styrene | UC | IA | Feces | A, O | GC-MS | [5] |
| Succinic acid | CD | All | Serum | Y, A, O | GC-MS | [11] |
| Taurine | UC | AC | Feces | A, O | ^1^H-NMR | [1] |
| Taurine | UC | All | Feces | A, O | ^1^H-NMR | [29] |
| Taurocholate | CD (ICD) | IA | Feces | Y, A, O | FT-ICR-MS | [17] |
| TG (20:5(5Z,8Z,11Z14Z,17Z)/22:6(4Z,7Z,10Z,13Z,16Z,19Z)/20:5(5Z,8Z,11Z,14Z,17Z))(d5) | CD | All | Plasma | A | LC-MS/MS | [8] |
| Thiomorpholine 3-carboxylate | UC | All | Plasma | A | LC-MS/MS | [8] |
| Threonine | CD | AC | Urine | A, O | ^1^H-NMR | [22] |
| Threonine | UC | AC | Urine | A, O | ^1^H-NMR | [22] |
| Toluene | UC | IA | Feces | A, O | GC-MS | [5] |
| Trehalose 6-palmitate, α,α- | CD | All | Plasma | A | LC-MS/MS | [8] |
| Triethylamine | CD | Unknown | Breath | A | SIFT-MS | [24] |
| Trihydroxy-6b-cholanate | CD (ICD) | IA | Feces | Y, A, O | FT-ICR-MS | [17] |
| Tryptophan | UC | AC | Urine | A, O | ^1^H-NMR | [22] |
| Tryptophan | UC | All | Urine | Y | ^1^H-NMR | [23] |
| Tryptophan | UC | All | Urine | Y | ^1^H-NMR | [23] |
| Tryptophan | UC | All | Urine | Y | ^1^H-NMR | [23] |
| Tryptophan | UC | All | Urine | Y | ^1^H-NMR | [23] |
| Tyrosine | CD | AC | Feces | A, O | ^1^H-NMR | [1] |
| Tyrosine | CD (ICD) | IA | Feces | Y, A, O | FT-ICR-MS | [17] |
| Ubiquinone 8 | CD | All | Plasma | A | LC-MS/MS | [8] |
| Unassigned metabolite 1 | UC | All | Urine | Y | ^1^H-NMR | [23] |
| Unassigned metabolite 2 | UC | All | Urine | Y | ^1^H-NMR | [23] |
| Unassigned metabolite 2 | UC | All | Urine | Y | ^1^H-NMR | [23] |
| Unassigned metabolite 3 | CD | All | Urine | Y | ^1^H-NMR | [23] |
| Unassigned metabolite 3 | CD | All | Urine | Y | ^1^H-NMR | [23] |
| Unassigned metabolite 3 | CD | All | Urine | Y | ^1^H-NMR | [23] |
| Unassigned metabolite 3 | CD | All | Urine | Y | ^1^H-NMR | [23] |
| Unassigned metabolite 3 | UC | All | Urine | Y | ^1^H-NMR | [23] |
| Unassigned metabolite 3 | UC | All | Urine | Y | ^1^H-NMR | [23] |
| Unassigned metabolite 3 | UC | All | Urine | Y | ^1^H-NMR | [23] |
| Unassigned metabolite 3 | UC | All | Urine | Y | ^1^H-NMR | [23] |
| Undecanal | CD | IA | Breath | A | GC-tof-MS | [19] |
| Unknown1 δ 1.39 ppm | IBD | AC | Serum | A, O | ^1^H-NMR | [26] |
| Unknown2 with 4-hydroxyphenyl group δ 6.85 ppm | IBD | AC | Urine | A, O | ^1^H-NMR | [26] |
| Unknown 7 | CD | All | Urine | A | ^1^H-NMR | [32] |
| Unknown 7 | UC | All | Urine | A | ^1^H-NMR | [32] |
| Uracil | UC | AC | Serum | Y, A, O | GC-MS | [18] |
| Uracil | UC | IA | Serum | Y, A, O | GC-MS | [18] |
| Uracil | UC | All | Serum | Y, A, O | GC-MS | [18] |
| Urea | UC | AC | Serum | Y, A, O | GC-MS | [18] |
| Urea | UC | IA | Serum | Y, A, O | GC-MS | [18] |
| Urea | UC | All | Serum | Y, A, O | GC-MS | [18] |
| Valine | CD | AC | Feces | A, O | ^1^H-NMR | [1] |
| Valine | CD | IA | Feces | A, O | ^1^H-NMR | [1] |
| Valine | CD | Unknown | Feces | Y, A, O | ^1^H-NMR | [27] |
| Valine | UC | AC | Feces | A, O | ^1^H-NMR | [1] |
| Valine | UC | AC | Feces | A, O | ^1^H-NMR | [1] |
| VOC2 (RT = 10.7 min) | CD | IA | Breath | A | GC-tof-MS | [19] |
| Xylose | CD | AC | Urine | A, O | ^1^H-NMR | [22] |
| Xylose_2 | UC | AC | Serum | Y, A, O | GC-MS | [18] |

‘-‘ indicates that no metabolites were found to be significantly increased in the respective sample.

Disease: CD: Crohn’s disease; CCD: colonic CD; IBD: inflammatory bowel disease; ICD: ileal CD; sb: small bowel; UC: ulcerative colitis. Activity: AC: active; IA: inactive; All: active + inactive. Age groups: Y: very early onset and young; A: adult; O: old. Platform: ESI-MS: electrospray ionization mass spectrometry; FT-ICR-MS: Fourier-transform ion cyclotron resonance mass spectrometry; GC-MS: gas chromatography-mass spectrometry; GC-tof-MS: gas chromatography time-of-flight mass spectrometry; LC-ESI-MS/MS: liquid chromatography electrospray ionization tandem mass spectrometry;(HP)LC-MS: (high performance) liquid chromatography-mass spectrometry; LC-MS/MS: liquid chromatography tandem mass spectrometry; MRS: magnetic resonance spectroscopy; NMR: nuclear magnetic resonance; SIFT-MS: selected-ion flow-tube mass spectrometry; UPLC/ToFMS: ultra performance liquid chromatography time-of-flight mass spectrometry. MGDG: monogalactosyldiacylglycerol; PBMC: peripheral blood mononuclear cells; PC: phosphatidylcholine; PS: phosphatidylserine; TG: triglyceride.

*O-(N-acetyl-a-neuraminosyl)-(2->3)-O-b-D-galactopyranosyl-(1->4)-O-2-(acetylamino)-2-deoxy-b-D-glucopyranosyl-(1->3)-O-b-D-galactopyranosyl-(1->4)-O-2-(acetylamino)-2-deoxy-b-D-glucopyranosyl-(1->3)-O-b-D-galactopyranosyl-(1->4)-D-Gluc.

**References**

1. Bjerrum, J.T.; Wang, Y.; Hao, F.; Coskun, M.; Ludwig, C.; Gunther, U.; Nielsen, O.H. Metabonomics of human fecal extracts characterize ulcerative colitis, Crohn's disease and healthy individuals. *Metabolomics : Official journal of the Metabolomic Society* **2015**, *11*, 122-133, doi:10.1007/s11306-014-0677-3.

2. Williams, H.R.; Cox, I.J.; Walker, D.G.; Cobbold, J.F.; Taylor-Robinson, S.D.; Marshall, S.E.; Orchard, T. Differences in gut microbial metabolism are responsible for reduced hippurate synthesis in Crohn's disease. *Gastroenterology* **2010**, *138*, S579.

3. Williams, H.R.T.; Cox, I.J.; Walker, D.G.; North, B.V.; Patel, V.M.; Marshall, S.E.; Jewell, D.P.; Ghosh, S.; Thomas, H.J.W.; Teare, J.P., et al. Characterization of inflammatory bowel disease with urinary metabolic profiling. *American Journal of Gastroenterology* **2009**, *104*, 1435-1444.

4. Sewell, G.W.; Hannun, Y.A.; Han, X.; Koster, G.; Bielawski, J.; Goss, V.; Smith, P.J.; Rahman, F.Z.; Vega, R.; Bloom, S.L., et al. Lipidomic profiling in Crohn's disease: abnormalities in phosphatidylinositols, with preservation of ceramide, phosphatidylcholine and phosphatidylserine composition. *The international journal of biochemistry & cell biology* **2012**, *44*, 1839-1846, doi:10.1016/j.biocel.2012.06.016.

5. Ahmed, I.; Greenwood, R.; Costello, B.; Ratcliffe, N.; Probert, C.S. Investigation of faecal volatile organic metabolites as novel diagnostic biomarkers in inflammatory bowel disease. *Alimentary Pharmacology and Therapeutics* **2016**, *43*, 596-611.

6. Cracowski, J.L.; Bonaz, B.; Bessard, G.; Bessard, J.; Anglade, C.; Fournet, J. Increased urinary F2-isoprostanes in patients with Crohn's disease. *American Journal of Gastroenterology* **2002**, *97*, 99-103.

7. De Preter, V.; Joossens, M.; Ballet, V.; Shkedy, Z.; Rutgeerts, P.; Vermeire, S.; Verbeke Phd, K. Metabolic profiling of the impact of oligofructose-enriched inulin in Crohn's disease patients: a double-blinded randomized controlled trial. *Clinical and translational gastroenterology* **2013**, *4*, e30, doi:10.1038/ctg.2012.24.

8. Yau, Y.Y.; Leong, R.W.L.; Shin, S.; Bustamante, S.; Pickford, R.; Hejazi, L.; Campbell, B.; Wasinger, V.C. Bimodal plasma metabolomics strategy identifies novel inflammatory metabolites in inflammatory bowel diseases. *Discovery medicine* **2014**, *18*, 113-124.

9. Shiomi, Y.; Nishiumi, S.; Ooi, M.; Hatano, N.; Shinohara, M.; Yoshie, T.; Kondo, Y.; Furumatsu, K.; Shiomi, H.; Kutsumi, H., et al. GCMS-based metabolomic study in mice with colitis induced by dextran sulfate sodium. *Inflammatory bowel diseases* **2011**, *17*, 2261-2274.

10. Machiels, K.; Joossens, M.; Sabino, J.; De Preter, V.; Arijs, I.; Eeckhaut, V.; Ballet, V.; Claes, K.; Van Immerseel, F.; Verbeke, K., et al. A decrease of the butyrate-producing species roseburia hominis and faecalibacterium prausnitzii defines dysbiosis in patients with ulcerative colitis. *Gut* **2014**, *63*, 1275-1283.

11. Ooi, M.; Nishiumi, S.; Yoshie, T.; Shiomi, Y.; Kohashi, M.; Fukunaga, K.; Nakamura, S.; Matsumoto, T.; Hatano, N.; Shinohara, M., et al. GC/MS-based profiling of amino acids and TCA cycle-related molecules in ulcerative colitis. *Inflammation Research* **2011**, *60*, 831-840.

12. De Preter, V.; Machiels, K.; Joossens, M.; Arijs, I.; Matthys, C.; Vermeire, S.; Rutgeerts, P.; Verbeke, K. Faecal metabolite profiling identifies medium-chain fatty acids as discriminating compounds in IBD. *Gut* **2015**, *64*, 447-458.

13. Stephens, N.S.; Siffledeen, J.; Su, X.; Murdoch, T.B.; Fedorak, R.N.; Slupsky, C.M. Urinary NMR metabolomic profiles discriminate inflammatory bowel disease from healthy. *Journal of Crohn's and Colitis* **2013**, *7*, e42-e48.

14. Balasubramanian, K.; Kumar, S.; Singh, R.R.; Sharma, U.; Ahuja, V.; Makharia, G.K.; Jagannathan, N.R. Metabolism of the colonic mucosa in patients with inflammatory bowel diseases: an in vitro proton magnetic resonance spectroscopy study. *Magnetic Resonance Imaging* **2009**, *27*, 79-86.

15. Hicks, L.C.; Huang, J.; Kumar, S.; Powles, S.T.; Orchard, T.R.; Hanna, G.B.; Williams, H.R. Analysis of Exhaled Breath Volatile Organic Compounds in Inflammatory Bowel Disease: A Pilot Study. *Journal of Crohn's & colitis* **2015**, *9*, 731-737.

16. Jacobs, J.P.; Goudarzi, M.; Singh, N.; Tong, M.; McHardy, I.H.; Ruegger, P.; Asadourian, M.; Moon, B.H.; Ayson, A.; Borneman, J., et al. A Disease-Associated Microbial and Metabolomics State in Relatives of Pediatric Inflammatory Bowel Disease Patients. *Cellular and molecular gastroenterology and hepatology* **2016**, *2*, 750-766.

17. Jansson, J.; Willing, B.; Lucio, M.; Fekete, A.; Dicksved, J.; Halfvarson, J.; Tysk, C.; Schmitt-Kopplin, P. Metabolomics reveals metabolic biomarkers of Crohn's disease. *PloS one* **2009**, *4*, e6386, doi:10.1371/journal.pone.0006386.

18. Kohashi, M.; Nishiumi, S.; Ooi, M.; Yoshie, T.; Matsubara, A.; Suzuki, M.; Hoshi, N.; Kamikozuru, K.; Yokoyama, Y.; Fukunaga, K., et al. A novel gas chromatography mass spectrometry-based serum diagnostic and assessment approach to ulcerative colitis. *Journal of Crohn's and Colitis* **2014**, *8*, 1010-1021.

19. Bodelier, A.G.L.; Smolinska, A.; Baranska, A.; Dallinga, J.W.; Mujagic, Z.; Vanhees, K.; Van Den Heuvel, T.; Masclee, A.A.M.; Jonkers, D.; Pierik, M.J., et al. Volatile organic compounds in exhaled air as novel marker for disease activity in Crohn's disease: A metabolomic approach. *Inflammatory bowel diseases* **2015**, *21*, 1776-1785.

20. Patel, N.; Alkhouri, N.; Eng, K.; Cikach, F.; Mahajan, L.; Yan, C.; Grove, D.; Rome, E.S.; Lopez, R.; Dweik, R.A. Metabolomic analysis of breath volatile organic compounds reveals unique breathprints in children with inflammatory bowel disease: A pilot study. *Alimentary Pharmacology and Therapeutics* **2014**, *40*, 498-507.

21. Iwamoto, J.; Saito, Y.; Honda, A.; Miyazaki, T.; Ikegami, T.; Matsuzaki, Y. Bile acid malabsorption deactivates pregnane x receptor in patients with Crohn's Disease. *Inflammatory bowel diseases* **2013**, *19*, 1278-1284.

22. Schicho, R.; Shaykhutdinov, R.; Ngo, J.; Nazyrova, A.; Schneider, C.; Panaccione, R.; Kaplan, G.G.; Vogel, H.J.; Storr, M. Quantitative metabolomic profiling of serum, plasma, and urine by 1H NMR spectroscopy discriminates between patients with inflammatory bowel disease and healthy individuals. *Journal of proteome research* **2012**, *11*, 3344-3357.

23. Martin, F.P.; Ezri, J.; Cominetti, O.; Da Silva, L.; Kussmann, M.; Godin, J.P.; Nydegger, A. Urinary metabolic phenotyping reveals differences in the metabolic status of healthy and inflammatory bowel disease (IBD) children in relation to growth and disease activity. *International journal of molecular sciences* **2016**, *17*, no pagination.

24. Rieder, F.; Kurada, S.; Grove, D.; Cikach, F.; Lopez, R.; Patel, N.; Singh, A.; Alkhouri, N.; Shen, B.; Brzezinski, A., et al. A Distinct Colon-Derived Breath Metabolome is Associated with Inflammatory Bowel Disease, but not its Complications. *Clinical and translational gastroenterology* **2016**, *7*, e201, doi:10.1038/ctg.2016.57.

25. Zhang, Y.; Lin, L.; Xu, Y.; Lin, Y.; Jin, Y.; Zheng, C. 1H NMR-based spectroscopy detects metabolic alterations in serum of patients with early-stage ulcerative colitis. *Biochemical and biophysical research communications* **2013**, *433*, 547-551.

26. Dawiskiba, T.; Deja, S.; Mulak, A.; Zabek, A.; Jawien, E.; Pawelka, D.; Banasik, M.; Mastalerz-Migas, A.; Balcerzak, W.; Kaliszewski, K., et al. Serum and urine metabolomic fingerprinting in diagnostics of inflammatory bowel diseases. *World journal of gastroenterology : WJG* **2014**, *20*, 163-174, doi:10.3748/wjg.v20.i1.163.

27. Marchesi, J.R.; Holmes, E.; Khan, F.; Kochhar, S.; Scanlan, P.; Shanahan, F.; Wilson, I.D.; Wang, Y. Rapid and noninvasive metabonomic characterization of inflammatory bowel disease. *Journal of proteome research* **2007**, *6*, 546-551.

28. Thyssen, E.; Turk, J.; Bohrer, A.; Stenson, W.F. Quantification of distinct molecular species of platelet activating factor in ulcerative colitis. *Lipids* **1996**, *31*, S255-S259.

29. Le Gall, G.; Noor, S.O.; Ridgway, K.; Scovell, L.; Jamieson, C.; Johnson, I.T.; Colquhoun, I.J.; Kemsley, E.K.; Narbad, A. Metabolomics of fecal extracts detects altered metabolic activity of gut microbiota in ulcerative colitis and irritable bowel syndrome. *Journal of proteome research* **2011**, *10*, 4208-4218.

30. Sharma, U.; Singh, R.R.; Ahuja, V.; Makharia, G.K.; Jagannathan, N.R. Similarity in the metabolic profile in macroscopically involved and un-involved colonic mucosa in patients with inflammatory bowel disease: An in vitro proton (1H) MR spectroscopy study. *Magnetic Resonance Imaging* **2010**, *28*, 1022-1029.

31. Fathi, F.; Majari-Kasmaee, L.; Mani-Varnosfaderani, A.; Kyani, A.; Rostami-Nejad, M.; Sohrabzadeh, K.; Naderi, N.; Zali, M.R.; Rezaei-Tavirani, M.; Tafazzoli, M., et al. 1H NMR based metabolic profiling in Crohn's disease by random forest methodology. *Magnetic resonance in chemistry : MRC* **2014**, *52*, 370-376.

32. Alonso, A.; Julia, A.; Vinaixa, M.; Domenech, E.; Fernandez-Nebro, A.; Canete, J.D.; Ferrandiz, C.; Tornero, J.; Gisbert, J.P.; Nos, P., et al. Urine metabolome profiling of immune-mediated inflammatory diseases. *BMC medicine* **2016**, *14*, 133, doi:10.1186/s12916-016-0681-8.

33. Johnson, J.C.; Schmidt, C.R.; Shrubsole, M.J.; Billheimer, D.D.; Joshi, P.R.; Morrow, J.D.; Heslin, M.J.; Washington, M.K.; Ness, R.M.; Zheng, W., et al. Urine PGE-M: A Metabolite of Prostaglandin E2 as a Potential Biomarker of Advanced Colorectal Neoplasia. *Clinical Gastroenterology and Hepatology* **2006**, *4*, 1358-1365.
