# Supplementary material for: Translational Potential of Metabolomics on Animal Models of Inflammatory Bowel Disease—A Systematic Critical Review
Source: Int J Mol Sci. 2020 May 29;21(11):3856. doi: 10.3390/ijms21113856 (PMC7312423; doi:10.3390/ijms21113856)
Supplement: Supplementary file 1 [file ijms-21-03856-s001.zip › Supplementary Table S6_resubmission_proofread.docx]

**Supplementary Table S6: Metabolites significantly decreased in IBD vs healthy controls in humans**

| **Metabolites ↓ in IBD** | **Disease** | **Activity** | **Sample type** | **Age group** | **Platform** | **Reference** |
| --- | --- | --- | --- | --- | --- | --- |
| - | CD | AC | Feces | A, O | ^1^H-NMR | [1] |
| - | CD | IA | Feces | A, O | ^1^H-NMR | [1] |
| - | UC | AC | Feces | A, O | ^1^H-NMR | [1] |
| - | UC | AC | Feces | A, O | ^1^H-NMR | [1] |
| - | CD | IA | PBMC Macrophages | A, O | ESI-MS | [2] |
| - | CD | AC | Urine | A, O | GC-MS | [3] |
| - | CD | IA | Feces | A, O | GC-MS | [4] |
| - | CD | IA | Urine | A, O | GC-MS | [3] |
| - | CD | IA | Feces | A | GC-MS | [5] |
| - | CD | IA | Plasma | A | GC-MS | [6] |
| - | UC | IA | Feces | A, O | GC-MS | [4] |
| - | UC | IA | Plasma | A | GC-MS | [6] |
| - | UC | All | Plasma | A | GC-MS | [6] |
| - | UC | AC | Colonic mucsa | A, O | GC-MS | [7] |
| - | CD | IA | PBMC Macrophages | A, O | HPLC-MS | [2] |
| - | CD | All | Serum | A | LC-ESI-MS/MS | [8] |
| - | CD | Unknown | Urine | A | LC-MS | [9] |
| - | CD | IA | Colonic mucosa | Unknown | Proton MRS | [10] |
| - | CD | Unknown | Breath | A | SIFT-MS | [11] |
| - | UC | Unknown | Breath | A | SIFT-MS | [11] |
| - | UC | IA | Feces | Unknown | UPLC/tof-MS | [12] |
| (Total SCFA) | UC | All | Feces | A | GC-MS | [13] |
| 1,4-Cyclohexadiene | UC | AC | Feces | A, O | GC-MS | [4] |
| 1,5-Anhydro-D-glucitol | UC | AC | Serum | Y, A, O | GC-MS | [14] |
| 1,5-Anhydro-D-glucitol | UC | IA | Serum | Y, A, O | GC-MS | [14] |
| 1,5-Anhydro-D-glucitol | UC | All | Serum | Y, A, O | GC-MS | [14] |
| 13-Sophorosyloxydocosanoic acid | UC | All | Plasma | A | LC-MS/MS | [6] |
| 17-Hydroxyandrostane-3-glucuronide | UC | All | Plasma | A | LC-MS/MS | [6] |
| 1-Ethyl-2,4-dimethylbenzene | CD | All | Feces | A | GC-MS | [5] |
| 1-Ethyl-3-methylbenzene | UC | IA | Feces | A, O | GC-tof-MS | [15] |
| 1-Ethyl-3-methylbenzene | UC | All | Feces | A, O | GC-tof-MS | [15] |
| 1-Hexadecanyl-2-((2/'-α-glucosyl)-β-glucosyl)-3-β-xylosyl-sn-glycerol | UC | All | Plasma | A | LC-MS/MS | [6] |
| 1-Hexanol | CD | All | Feces | A | GC-MS | [5] |
| 1-Hexanol | CD | IA | Feces | A, O | GC-tof-MS | [15] |
| 1-Hexanol | CD | All | Feces | A, O | GC-tof-MS | [15] |
| 1-Hexanol | UC | All | Feces | A, O | GC-tof-MS | [15] |
| 1-Hexanol | IBD | All | Feces | A, O | GC-tof-MS | [15] |
| 1-Methyl-2-(1-methylethyl)-benzene | UC | IA | Feces | A, O | GC-tof-MS | [15] |
| 1-Methyl-2-(1-methylethyl)-benzene | UC | All | Feces | A, O | GC-tof-MS | [15] |
| 1-Methyl-4-(1-methylethenyl)-benzene | CD | All | Feces | A | GC-MS | [5] |
| 1-Methyl-4-(1-methylethenyl)-benzene | CD | IA | Feces | A, O | GC-tof-MS | [15] |
| 1-Methyl-4-(1-methylethenyl)-benzene | CD | All | Feces | A, O | GC-tof-MS | [15] |
| 1-Methyl-4-(1-methylethenyl)-benzene | UC | IA | Feces | A, O | GC-tof-MS | [15] |
| 1-Methyl-4-(1-methylethenyl)-benzene | UC | All | Feces | A, O | GC-tof-MS | [15] |
| 1-Methyl-4-(1-methylethenyl)-benzene | IBD | All | Feces | A, O | GC-tof-MS | [15] |
| 1-Methyl-4-(1-methylethenyl)-benzene | IBD | IA | Feces | A, O | GC-tof-MS | [15] |
| 1-Methyl-4-[1-methylethyldiene]-cyclohexene | CD (sb) | AC | Feces | A, O | GC-MS | [4] |
| 1-Methylhistidine | IBD | All | Urine | A, O | NMR | [16] |
| 1-Nitroheptane | CD | AC | Feces | A, O | GC-MS | [4] |
| 1-Nonene | IBD | AC | Breath | Y, A | SIFT-MS | [17] |
| 1-Nonene | IBD | AC | Breath | Y, A | SIFT-MS | [17] |
| 1-O-(2-Acetamido-2-deoxy-α-D-glucopyranosyl)-1D-myo-inositol 3-phosphate | UC | All | Plasma | A | LC-MS/MS | [6] |
| 2,2,4-Trimethyl pentane | IBD | All | Feces | A, O | GC-tof-MS | [15] |
| 2,2,4-Trimethyl pentane | IBD | IA | Feces | A, O | GC-tof-MS | [15] |
| 2,3-Pentanedione | UC | AC | Feces | A, O | GC-MS | [4] |
| 2,5-Dimethyl furan | CD | All | Feces | A | GC-MS | [5] |
| 2,5-Dimethyl furan | CD | IA | Feces | A, O | GC-tof-MS | [15] |
| 2,5-Dimethyl furan | CD | All | Feces | A, O | GC-tof-MS | [15] |
| 2,5-Dimethyl furan | UC | IA | Feces | A, O | GC-tof-MS | [15] |
| 2,5-Dimethyl furan | UC | All | Feces | A, O | GC-tof-MS | [15] |
| 2,5-Dimethyl furan | IBD | All | Feces | A, O | GC-tof-MS | [15] |
| 2,5-Dimethyl furan | IBD | IA | Feces | A, O | GC-tof-MS | [15] |
| 25-Hydroxycholesterol | CD | All | Serum | A | LC-ESI-MS/MS | [8] |
| 2-Aminobutyric acid | UC | IA | Serum | Y, A, O | GC-MS | [14] |
| 2-Aminobutyric acid | UC | All | Serum | Y, A, O | GC-MS | [14] |
| 2-Aminoethanol | UC | AC | Serum | Y, A, O | GC-MS | [14] |
| 2-Aminoethanol | UC | IA | Serum | Y, A, O | GC-MS | [14] |
| 2-Aminoethanol | UC | All | Serum | Y, A, O | GC-MS | [14] |
| 2-Aminoisobutyrate | UC | IA | Serum | Y, A, O | GC-MS | [14] |
| 2-Butanone | CD | All | Feces | A | GC-MS | [5] |
| 2-Butanone | CD | IA | Feces | A, O | GC-tof-MS | [15] |
| 2-Butanone | CD | All | Feces | A, O | GC-tof-MS | [15] |
| 2-Butanone | UC | IA | Feces | A, O | GC-tof-MS | [15] |
| 2-Butanone | UC | All | Feces | A, O | GC-tof-MS | [15] |
| 2-Butanone | IBD | All | Feces | A, O | GC-tof-MS | [15] |
| 2-Butanone | IBD | IA | Feces | A, O | GC-tof-MS | [15] |
| 2-Dehydro-D-gluconate_1 | UC | IA | Serum | Y, A, O | GC-MS | [14] |
| 2-Dodecanone | CD | AC | Feces | A, O | GC-MS | [4] |
| 2-Ethyl-5-methyl-furan | CD | IA | Feces | A, O | GC-tof-MS | [15] |
| 2-Ethyl-5-methyl-furan | CD | All | Feces | A, O | GC-tof-MS | [15] |
| 2-Ethyl-5-methyl-furan | UC | All | Feces | A, O | GC-tof-MS | [15] |
| 2-Ethyl-5-methyl-furan | IBD | All | Feces | A, O | GC-tof-MS | [15] |
| 2-Ethyl-5-methyl-furan | IBD | IA | Feces | A, O | GC-tof-MS | [15] |
| 2-Hexanone | UC | AC | Feces | A, O | GC-MS | [4] |
| 2-Hexanone | CD | IA | Feces | A, O | GC-tof-MS | [15] |
| 2-Hexanone | CD | All | Feces | A, O | GC-tof-MS | [15] |
| 2-Hexanone | UC | IA | Feces | A, O | GC-tof-MS | [15] |
| 2-Hexanone | UC | All | Feces | A, O | GC-tof-MS | [15] |
| 2-Hexanone | IBD | All | Feces | A, O | GC-tof-MS | [15] |
| 2-Hexanone | IBD | IA | Feces | A, O | GC-tof-MS | [15] |
| 2-Hydroxyisobutyrate | IBD | AC | Urine | A, O | ^1^H-NMR | [18] |
| 2-Methylbutanoate | CD | All | Feces | A, O | GC-tof-MS | [15] |
| 2-Methylbutanoate | UC | All | Feces | A, O | GC-tof-MS | [15] |
| 2-Methylbutanoate | IBD | All | Feces | A, O | GC-tof-MS | [15] |
| 2-Methylbutanoic acid | CD | IA | Feces | A, O | GC-tof-MS | [15] |
| 2-Methylbutanoic acid | UC | IA | Feces | A, O | GC-tof-MS | [15] |
| 2-Methylbutanoic acid | IBD | IA | Feces | A, O | GC-tof-MS | [15] |
| 2-Methylbutyrate | UC | All | Feces | A, O | ^1^H-NMR | [19] |
| 2-Methylbutyrate | CD | All | Feces | A | GC-MS | [5] |
| 2-Methylpropanoic acid | CD | IA | Feces | A, O | GC-tof-MS | [15] |
| 2-Methylpropanoic acid | CD | All | Feces | A, O | GC-tof-MS | [15] |
| 2-Nonene, (E)- | IBD | AC | Breath | Y, A | SIFT-MS | [17] |
| 2-Nonene, (E)- | IBD | AC | Breath | Y, A | SIFT-MS | [17] |
| 2-Oxoisocaproate | CD | AC | Serum | A, O | ^1^H-NMR | [20] |
| 2-Oxoisocaproate | CD | AC | Urine | A, O | ^1^H-NMR | [20] |
| 2-Pentyl furan | CD | All | Feces | A | GC-MS | [5] |
| 2-Pentyl furan | CD | IA | Feces | A, O | GC-tof-MS | [15] |
| 2-Pentyl furan | CD | All | Feces | A, O | GC-tof-MS | [15] |
| 2-Undecanone | UC | AC | Feces | A, O | GC-MS | [4] |
| 3,4-Dimethylthiophene | UC | IA | Feces | A, O | GC-tof-MS | [15] |
| 3,4-Dimethylthiophene | UC | All | Feces | A, O | GC-tof-MS | [15] |
| 3-Carene | CD | All | Feces | A, O | GC-tof-MS | [15] |
| 3-Hexanone | UC | AC | Feces | A, O | GC-MS | [4] |
| 3-Hydroxyisobutyrate | CD | All | Urine | Y | ^1^H-NMR | [21] |
| 3-Hydroxyisobutyrate | UC | All | Urine | Y | ^1^H-NMR | [21] |
| 3-Hydroxyisovalerate | CD | All | Urine | A | ^1^H-NMR | [22] |
| 3-Methyl, 1-butanol | CD | AC | Feces | A, O | GC-MS | [4] |
| 3-Methyl-1H-indole | CD | All | Feces | A | GC-MS | [5] |
| 3-Methyl-1H-Indole | CD | IA | Feces | A, O | GC-tof-MS | [15] |
| 3-Methyl-1H-indole | CD | All | Feces | A, O | GC-tof-MS | [15] |
| 3-Methyl-1H-Indole | UC | IA | Feces | A, O | GC-tof-MS | [15] |
| 3-Methyl-1H-indole | UC | All | Feces | A, O | GC-tof-MS | [15] |
| 3-Methyl-1H-indole | IBD | All | Feces | A, O | GC-tof-MS | [15] |
| 3-Methyl-1H-Indole | IBD | IA | Feces | A, O | GC-tof-MS | [15] |
| 3-Methyl-phenol | CD | AC | Feces | A, O | GC-MS | [4] |
| 3-Phenyl-2-propenal | CD | All | Feces | A | GC-MS | [5] |
| 4-[[5-(acetylamino)-1H-indol-3-y]-3-methoxy-N-[(2-methylphenyl)sulfonyl]-Benzamide | CD | All | Plasma | A | LC-MS/MS | [6] |
| 4-Cresol sulfate | CD | All | Urine | Y, A, O | ^1^H-NMR | [23] |
| 4-Cresol sulfate | CD (CCD) | All | Urine | Y, A, O | ^1^H-NMR | [23] |
| 4-Hydroxy-L-proline, trans- | UC | AC | Serum | Y, A, O | GC-MS | [14] |
| 4-Hydroxy-L-proline, trans- | UC | IA | Serum | Y, A, O | GC-MS | [14] |
| 4-Hydroxy-L-proline, trans- | UC | All | Serum | Y, A, O | GC-MS | [14] |
| 4-Hydroxymandelate | UC | AC | Serum | Y, A, O | GC-MS | [14] |
| 4-Hydroxymandelate | UC | IA | Serum | Y, A, O | GC-MS | [14] |
| 4-Hydroxymandelate | UC | All | Serum | Y, A, O | GC-MS | [14] |
| 4-Hydroxyphenylpyruvate | CD | All | Urine | Y | ^1^H-NMR | [21] |
| 4β-Hydroxycholesterol | CD | All | Serum | A | LC-ESI-MS/MS | [8] |
| 5-Aminovaleric acid | UC | IA | Serum | Y, A, O | GC-MS | [14] |
| 6-Methyl-3,5-heptadiene-2-one | CD | All | Feces | A | GC-MS | [5] |
| 6-Methyl-5-hepten-2-one | CD | All | Feces | A, O | GC-tof-MS | [15] |
| 6-Methyl-5-hepten-2-one | UC | All | Feces | A, O | GC-tof-MS | [15] |
| 6-Methyl-5-hepten-2-one | IBD | All | Feces | A, O | GC-tof-MS | [15] |
| Acetaldehyde | UC | IA | Feces | A, O | GC-tof-MS | [15] |
| Acetaldehyde | UC | All | Feces | A, O | GC-tof-MS | [15] |
| Acetate | CD | AC | Serum | A, O | ^1^H-NMR | [20] |
| Acetate | CD | Unknown | Feces | Y, A, O | ^1^H-NMR | [24] |
| Acetate | UC | AC | Serum | A, O | ^1^H-NMR | [20] |
| Acetate | UC | All | Feces | A | GC-MS | [13] |
| Acetate | IBD | All | Urine | A, O | NMR | [16] |
| Acetic acid | UC | AC | Feces | A, O | GC-MS | [4] |
| Acetic acid pentyl ester | CD (sb) | AC | Feces | A, O | GC-MS | [4] |
| Acetoacetic acid | UC | AC | Serum | Y, A, O | GC-MS | [14] |
| Acetoacetic acid | UC | IA | Serum | Y, A, O | GC-MS | [14] |
| Acetoacetic acid | UC | All | Serum | Y, A, O | GC-MS | [14] |
| Acetone | CD | AC | Breath | A | GC-tof-MS | [25] |
| Acetone | CD | IA | Breath | A | GC-tof-MS | [25] |
| Acetone | UC | IA | Feces | A, O | GC-tof-MS | [15] |
| Acetone | UC | All | Feces | A, O | GC-tof-MS | [15] |
| Acetophenone | CD | AC | Feces | A | GC-MS | [5] |
| Acetophenone | CD | All | Feces | A | GC-MS | [5] |
| Acetophenone | CD | IA | Feces | A, O | GC-tof-MS | [15] |
| Acetophenone | CD | All | Feces | A, O | GC-tof-MS | [15] |
| Acetophenone | UC | IA | Feces | A, O | GC-tof-MS | [15] |
| Acetophenone | UC | All | Feces | A, O | GC-tof-MS | [15] |
| Acetophenone | IBD | All | Feces | A, O | GC-tof-MS | [15] |
| Acetophenone | IBD | IA | Feces | A, O | GC-tof-MS | [15] |
| Acetyl Aas, N- | CD | All | Urine | A | ^1^H-NMR | [22] |
| Acetyl Aas, N- | UC | All | Urine | A | ^1^H-NMR | [22] |
| Acetylaspartic acid, N- | UC | All | Rectum tissue | Y, A, O | GC-MS | [26] |
| Acetylcarnitine, O- | UC | AC | Serum | A, O | ^1^H-NMR | [20] |
| Acetylglutamic acid | CD | IA | Feces | Unknown | UPLC/tof-MS | [12] |
| Aconitate | UC | AC | Serum | Y, A, O | GC-MS | [14] |
| Aconitate, cis- | CD | All | Urine | Y | ^1^H-NMR | [21] |
| Aconitate, cis- | CD | All | Urine | Y | ^1^H-NMR | [21] |
| Aconitate, cis- | CD | All | Urine | Y | ^1^H-NMR | [21] |
| Aconitate, cis- | CD | All | Urine | Y | ^1^H-NMR | [21] |
| Aconitate, cis- | UC | All | Urine | Y | ^1^H-NMR | [21] |
| Aconitate, cis- | UC | All | Urine | Y | ^1^H-NMR | [21] |
| Aconitate, cis- | UC | All | Urine | Y | ^1^H-NMR | [21] |
| Aconitate, cis- | UC | All | Urine | Y | ^1^H-NMR | [21] |
| Aconitate, Trans- | IBD | All | Urine | A, O | NMR | [16] |
| Acylcarnitine | UC | All | Urine | Y | ^1^H-NMR | [21] |
| Acylcarnitine | UC | All | Urine | Y | ^1^H-NMR | [21] |
| Alanine | CD | All | Urine | A | ^1^H-NMR | [22] |
| Alanine | IBD | AC | Colonic mucosa | A | ^1^H-NMR | [27] |
| Alanine | IBD | IA | Urine | A, O | ^1^H-NMR | [18] |
| Alanine | UC | All | Rectum tissue | Y, A, O | GC-MS | [26] |
| Alanine | CD | AC | Colonic mucosa | Unknown | Proton MRS | [10] |
| Alanine | UC | AC | Colonic mucosa | Unknown | Proton MRS | [10] |
| Ammonia | UC | All | Breath | A, O | SIFT-MS | [28] |
| Ammonia | IBD | AC | Breath | Y, A | SIFT-MS | [17] |
| Arabinose | UC | IA | Serum | Y, A, O | GC-MS | [14] |
| Arabitol | UC | AC | Serum | Y, A, O | GC-MS | [14] |
| Asparagine | UC | AC | Serum | Y, A, O | GC-MS | [14] |
| Asparagine | UC | IA | Serum | Y, A, O | GC-MS | [14] |
| Asparagine | UC | All | Serum | Y, A, O | GC-MS | [14] |
| Asparagine | UC | All | Rectum tissue | Y, A, O | GC-MS | [26] |
| Asparagine | UC | All | Serum | Y, A, O | GC-MS | [26] |
| Asparagine | IBD | All | Urine | A, O | NMR | [16] |
| Aspartic acid | CD | IA | Feces | A, O | ^1^H-NMR | [1] |
| Aspartic acid | UC | AC | Serum | Y, A, O | GC-MS | [14] |
| Aspartic acid | UC | IA | Serum | Y, A, O | GC-MS | [14] |
| Aspartic acid | UC | All | Serum | Y, A, O | GC-MS | [14] |
| Benzaldehyde | UC | IA | Feces | A, O | GC-tof-MS | [15] |
| Benzaldehyde | UC | All | Feces | A, O | GC-tof-MS | [15] |
| Benzene acetaldehyde | UC | AC | Feces | A, O | GC-MS | [4] |
| Benzyl-L-cysteine_1, S- | UC | AC | Serum | Y, A, O | GC-MS | [14] |
| Benzyl-L-cysteine_1, S- | UC | IA | Serum | Y, A, O | GC-MS | [14] |
| Benzyl-L-cysteine_1, S- | UC | All | Serum | Y, A, O | GC-MS | [14] |
| Betaine | CD | AC | Plasma | A, O | ^1^H-NMR | [20] |
| Betaine | CD | AC | Urine | A, O | ^1^H-NMR | [20] |
| Betaine | UC | AC | Plasma | A, O | ^1^H-NMR | [20] |
| Betaine | UC | AC | Urine | A, O | ^1^H-NMR | [20] |
| Bicycle[3.1.0]hexane, 4-methylene-1-methylethyl | CD | AC | Feces | A, O | GC-MS | [4] |
| Boldione | CD | IA | Feces | Unknown | UPLC/tof-MS | [12] |
| Butanoic acid | CD | AC | Feces | A, O | GC-MS | [4] |
| Butanoic acid | UC | AC | Feces | A, O | GC-MS | [4] |
| Butanoic acid ethyl ester | CD (sb) | AC | Feces | A, O | GC-MS | [4] |
| Butanoic acid, 2-methyl ester | CD (sb) | AC | Feces | A, O | GC-MS | [4] |
| Butanoic acid, 2-methyl-butyle ester | CD (sb) | AC | Feces | A, O | GC-MS | [4] |
| Butyrate | CD | AC | Feces | A, O | ^1^H-NMR | [1] |
| Butyrate | CD | Unknown | Feces | Y, A, O | ^1^H-NMR | [24] |
| Butyrate | CD | AC | Feces | A | GC-MS | [5] |
| C20-OH sulfatide | CD | All | Plasma | A | LC-MS/MS | [6] |
| Caprylic acid, n- | UC | AC | Serum | Y, A, O | GC-MS | [14] |
| Caprylic acid, n- | UC | All | Serum | Y, A, O | GC-MS | [14] |
| Carbon disulfide | UC | AC | Feces | A, O | GC-MS | [4] |
| Carnitine | CD | All | Urine | Y | ^1^H-NMR | [21] |
| Carnitine | CD | All | Urine | Y | ^1^H-NMR | [21] |
| Carnitine | UC | All | Urine | Y | ^1^H-NMR | [21] |
| Choline | CD | AC | Plasma | A, O | ^1^H-NMR | [20] |
| Choline | UC | AC | Serum | A, O | ^1^H-NMR | [20] |
| Choline | UC | AC | Plasma | A, O | ^1^H-NMR | [20] |
| Choline | IBD | AC | Colonic mucosa | A | ^1^H-NMR | [27] |
| Choline | CD | AC | Colonic mucosa | Unknown | Proton MRS | [10] |
| Choline | UC | AC | Colonic mucosa | Unknown | Proton MRS | [10] |
| Choline + PC + GPC | IBD | AC | Serum | A, O | ^1^H-NMR | [18] |
| Citrate | UC | All | Urine | A | ^1^H-NMR | [22] |
| Citrate | CD | AC | Serum | A, O | ^1^H-NMR | [20] |
| Citrate | CD | All | Urine | A | ^1^H-NMR | [22] |
| Citrate | UC | AC | Serum | A, O | ^1^H-NMR | [20] |
| Citrate | UC | AC | Urine | A, O | ^1^H-NMR | [20] |
| Citrate | IBD | AC | Urine | A, O | ^1^H-NMR | [18] |
| Citrate | IBD | IA | Urine | A, O | ^1^H-NMR | [18] |
| Citrate | IBD | All | Urine | A, O | NMR | [16] |
| Citrate, 1-Methylnicotinamide | CD | AC | Urine | A, O | ^1^H-NMR | [20] |
| Citric acid | UC | All | Rectum tissue | Y, A, O | GC-MS | [26] |
| Citric acid + isocitric acid | UC | AC | Serum | Y, A, O | GC-MS | [14] |
| Citric acid + isocitric acid | UC | IA | Serum | Y, A, O | GC-MS | [14] |
| Citric acid + isocitric acid | UC | All | Serum | Y, A, O | GC-MS | [14] |
| Citrulline | UC | AC | Serum | Y, A, O | GC-MS | [14] |
| Citrulline | UC | IA | Serum | Y, A, O | GC-MS | [14] |
| Citrulline | UC | All | Serum | Y, A, O | GC-MS | [14] |
| Copaene | UC | AC | Feces | A, O | GC-MS | [4] |
| Creatine | IBD | AC | Serum | A, O | ^1^H-NMR | [18] |
| Creatine | IBD | All | Urine | A, O | NMR | [16] |
| Creatinine | CD | AC | Plasma | A, O | ^1^H-NMR | [20] |
| Creatinine | UC | AC | Serum | A, O | ^1^H-NMR | [20] |
| Creatinine | UC | AC | Plasma | A, O | ^1^H-NMR | [20] |
| Creatinine | UC | AC | Urine | A, O | ^1^H-NMR | [20] |
| Creatinine | UC | AC | Serum | Y, A, O | GC-MS | [14] |
| Creatinine | UC | All | Serum | Y, A, O | GC-MS | [14] |
| Cresol, p- | CD | AC | Feces | A | GC-MS | [5] |
| Cresol, p- | CD | IA | Feces | A, O | GC-tof-MS | [15] |
| Cresol, p- | CD | All | Feces | A, O | GC-tof-MS | [15] |
| Cresol, p- | UC | IA | Feces | A, O | GC-tof-MS | [15] |
| Cresol, p- | UC | All | Feces | A, O | GC-tof-MS | [15] |
| Cresol, p- | IBD | All | Feces | A, O | GC-tof-MS | [15] |
| Cresol, p- | IBD | IA | Feces | A, O | GC-tof-MS | [15] |
| Cyclohexanone | CD | All | Feces | A | GC-MS | [5] |
| Cyclohexanone | UC | All | Feces | A, O | GC-tof-MS | [15] |
| Cytidine diphosphate diacylglycerol (18:0/18:0) | UC | All | Plasma | A | LC-MS/MS | [6] |
| Dimethyl disulfide | UC | IA | Feces | A, O | GC-tof-MS | [15] |
| Dimethyl disulfide | UC | All | Feces | A, O | GC-tof-MS | [15] |
| Dimethyl trisulfide | UC | IA | Feces | A, O | GC-tof-MS | [15] |
| Dimethyl trisulfide | UC | All | Feces | A, O | GC-tof-MS | [15] |
| Dimethylglycine, N,N- | CD | All | Urine | A | ^1^H-NMR | [22] |
| Dimethylsulfide | IBD | All | Feces | A, O | GC-tof-MS | [15] |
| Dimethylsulfide | CD | All | Feces | A | GC-MS | [5] |
| Dimethylsulfide | CD | IA | Feces | A, O | GC-tof-MS | [15] |
| Dimethylsulfide | CD | All | Feces | A, O | GC-tof-MS | [15] |
| Dimethylsulfide | UC | IA | Feces | A, O | GC-tof-MS | [15] |
| Dimethylsulfide | UC | All | Feces | A, O | GC-tof-MS | [15] |
| Dimethylsulfide | IBD | IA | Feces | A, O | GC-tof-MS | [15] |
| Dimethylsulfone | UC | AC | Plasma | A, O | ^1^H-NMR | [20] |
| Dimethylsulfone | IBD | AC | Serum | A, O | ^1^H-NMR | [18] |
| Dimethylsulfone | IBD | IA | Serum | A, O | ^1^H-NMR | [18] |
| dTDP-D-forosamine | CD | All | Plasma | A | LC-MS/MS | [6] |
| Ethanolamine | CD | AC | Urine | A, O | ^1^H-NMR | [20] |
| Formate | CD | AC | Urine | A, O | ^1^H-NMR | [20] |
| Formate | CD | All | Urine | Y | ^1^H-NMR | [21] |
| Formate | IBD | AC | Colonic mucosa | A | ^1^H-NMR | [27] |
| Formate | IBD | IA | Urine | A, O | ^1^H-NMR | [18] |
| Formate | IBD | All | Urine | A, O | NMR | [16] |
| Formate | CD | AC | Colonic mucosa | Unknown | Proton MRS | [10] |
| Formate | UC | AC | Colonic mucosa | Unknown | Proton MRS | [10] |
| Free acetate | CD | All | Urine | A | ^1^H-NMR | [22] |
| Fructose_1 | UC | AC | Serum | Y, A, O | GC-MS | [14] |
| Fucα1-2Galα1-3Galβ1-4Glcβ-Cer(d18:1/24:1(15Z))† | UC | All | Plasma | A | LC-MS/MS | [6] |
| Fumaric acid | UC | AC | Serum | Y, A, O | GC-MS | [14] |
| Fumaric acid | UC | IA | Serum | Y, A, O | GC-MS | [14] |
| Fumaric acid | UC | All | Serum | Y, A, O | GC-MS | [14] |
| Fumaric acid | UC | All | Rectum tissue | Y, A, O | GC-MS | [26] |
| Furan | UC | All | Feces | A, O | GC-tof-MS | [15] |
| Galactose | UC | AC | Urine | A, O | ^1^H-NMR | [20] |
| Galα1-3(Fucα1-2)Galβ1-4Glcβ-Cer(d18:1/20:0)† | UC | All | Plasma | A | LC-MS/MS | [6] |
| Galα1-3(Fucα1-2)Galβ1-4Glcβ-Cer(d18:1/24:1(15Z)) | UC | All | Plasma | A | LC-MS/MS | [6] |
| Ganglioside GM3 (d18:0/23:0)† | UC | All | Plasma | A | LC-MS/MS | [6] |
| Ganglioside GM3 (d18:0/24:0)† | UC | All | Plasma | A | LC-MS/MS | [6] |
| Ganglioside GM3 (d18:0/25:0)† | UC | All | Plasma | A | LC-MS/MS | [6] |
| Glucarate | UC | AC | Serum | Y, A, O | GC-MS | [14] |
| Glucarate | UC | IA | Serum | Y, A, O | GC-MS | [14] |
| Glucarate | UC | All | Serum | Y, A, O | GC-MS | [14] |
| Glucopyranosiduronic acid, (3a,5b)-24-[(carboxymethyl)amino]-24-oxocholan-3-yl, β-D- | UC | All | Plasma | A | LC-MS/MS | [6] |
| Glucose | CD | AC | Plasma | A, O | ^1^H-NMR | [20] |
| Glucuronate_1 | UC | AC | Serum | Y, A, O | GC-MS | [14] |
| Glucuronate_1 | UC | IA | Serum | Y, A, O | GC-MS | [14] |
| Glucuronate_1 | UC | All | Serum | Y, A, O | GC-MS | [14] |
| Glutamate | CD | IA | Feces | A, O | ^1^H-NMR | [1] |
| Glutamate | CD | AC | Colonic mucosa | Unknown | Proton MRS | [10] |
| Glutamate | UC | AC | Colonic mucosa | Unknown | Proton MRS | [10] |
| Glutamic acid | UC | IA | Serum | Y, A, O | GC-MS | [14] |
| Glutamic acid | UC | All | Serum | Y, A, O | GC-MS | [14] |
| Glutamic acid | UC | All | Rectum tissue | Y, A, O | GC-MS | [26] |
| Glutamine | CD | AC | Plasma | A, O | ^1^H-NMR | [20] |
| Glutamine | CD | AC | Urine | A, O | ^1^H-NMR | [20] |
| Glutamine | CD | All | Serum | Y, A, O | GC-MS | [26] |
| Glutamine | UC | AC | Serum | Y, A, O | GC-MS | [14] |
| Glutamine | UC | AC | Serum | A, O | GC-MS | [29] |
| Glutamine | UC | IA | Serum | Y, A, O | GC-MS | [14] |
| Glutamine | UC | All | Serum | Y, A, O | GC-MS | [14] |
| Glutamine | UC | All | Rectum tissue | Y, A, O | GC-MS | [26] |
| Glutamine | UC | All | Serum | Y, A, O | GC-MS | [26] |
| Glutamine | CD | AC | Colonic mucosa | Unknown | Proton MRS | [10] |
| Glutamine | UC | AC | Colonic mucosa | Unknown | Proton MRS | [10] |
| Glutamine/Glutamate | IBD | AC | Colonic mucosa | A | ^1^H-NMR | [27] |
| Glyceric acid | UC | AC | Serum | Y, A, O | GC-MS | [14] |
| Glyceric acid | UC | All | Serum | Y, A, O | GC-MS | [14] |
| Glycerol | UC | IA | Serum | Y, A, O | GC-MS | [14] |
| Glycerophosphocholine/phosphocholine | IBD | AC | Colonic mucosa | A | ^1^H-NMR | [27] |
| Glycerophosphorylcholine | CD | AC | Colonic mucosa | Unknown | Proton MRS | [10] |
| Glycerophosphorylcholine | UC | AC | Colonic mucosa | Unknown | Proton MRS | [10] |
| Glycerophosphorylcholine | UC | IA | Colonic mucosa | Unknown | Proton MRS | [10] |
| Glycine | IBD | IA | Urine | A, O | ^1^H-NMR | [18] |
| Glycine | UC | All | Rectum tissue | Y, A, O | GC-MS | [26] |
| Glycolate | CD | AC | Urine | A, O | ^1^H-NMR | [20] |
| Glycolic acid | UC | AC | Serum | Y, A, O | GC-MS | [14] |
| Glycyl-glycine_1 | UC | AC | Serum | Y, A, O | GC-MS | [14] |
| Glycyl-glycine_1 | UC | All | Serum | Y, A, O | GC-MS | [14] |
| Heptanoate | CD | All | Feces | A | GC-MS | [5] |
| Heptanoate | CD | All | Feces | A, O | GC-tof-MS | [15] |
| Heptanoate | UC | All | Feces | A, O | GC-tof-MS | [15] |
| Heptanoate | IBD | All | Feces | A, O | GC-tof-MS | [15] |
| Heptanoic acid | CD | IA | Feces | A, O | GC-tof-MS | [15] |
| Heptanoic acid | UC | IA | Feces | A, O | GC-tof-MS | [15] |
| Heptanoic acid | IBD | IA | Feces | A, O | GC-tof-MS | [15] |
| Heptaprenyl diphosphate, All-trans- | UC | All | Plasma | A | LC-MS/MS | [6] |
| Hexane | CD | All | Feces | A | GC-MS | [5] |
| Hexane | CD | IA | Feces | A, O | GC-tof-MS | [15] |
| Hexane | CD | All | Feces | A, O | GC-tof-MS | [15] |
| Hexane | IBD | All | Feces | A, O | GC-tof-MS | [15] |
| Hexane | IBD | IA | Feces | A, O | GC-tof-MS | [15] |
| Hexanoate | CD | All | Feces | A | GC-MS | [5] |
| Hexanoate | CD | All | Feces | A, O | GC-tof-MS | [15] |
| Hexanoate | UC | All | Feces | A, O | GC-tof-MS | [15] |
| Hexanoate | IBD | All | Feces | A, O | GC-tof-MS | [15] |
| Hexanoic acid | CD | IA | Feces | A, O | GC-tof-MS | [15] |
| Hexanoic acid | UC | IA | Feces | A, O | GC-tof-MS | [15] |
| Hexanoic acid | IBD | IA | Feces | A, O | GC-tof-MS | [15] |
| Hippurate | CD | AC | Urine | A, O | ^1^H-NMR | [20] |
| Hippurate | CD | IA | Urine | A, O | ^1^H-NMR | [30] |
| Hippurate | CD | All | Urine | A | ^1^H-NMR | [22] |
| Hippurate | CD | All | Urine | Y | ^1^H-NMR | [21] |
| Hippurate | CD | All | Urine | Y | ^1^H-NMR | [21] |
| Hippurate | CD | All | Urine | Y | ^1^H-NMR | [21] |
| Hippurate | CD | All | Urine | Y | ^1^H-NMR | [21] |
| Hippurate | CD | All | Urine | Y, A, O | ^1^H-NMR | [23] |
| Hippurate | CD (CCD) | All | Urine | Y, A, O | ^1^H-NMR | [23] |
| Hippurate | UC | AC | Urine | A, O | ^1^H-NMR | [20] |
| Hippurate | UC | All | Urine | Y | ^1^H-NMR | [21] |
| Hippurate | UC | All | Urine | Y | ^1^H-NMR | [21] |
| Hippurate | UC | All | Urine | Y | ^1^H-NMR | [21] |
| Hippurate | UC | All | Urine | Y | ^1^H-NMR | [21] |
| Hippurate | UC | All | Urine | Y, A, O | ^1^H-NMR | [23] |
| Hippurate | IBD | AC | Urine | A, O | ^1^H-NMR | [18] |
| Hippurate | IBD | IA | Urine | A, O | ^1^H-NMR | [18] |
| Hippurate | IBD | All | Urine | A, O | NMR | [16] |
| Hippurate | UC | All | Urine | A | ^1^H-NMR | [22] |
| Histidine | IBD | AC | Serum | A, O | ^1^H-NMR | [18] |
| Histidine | CD | All | Serum | Y, A, O | GC-MS | [26] |
| Histidine | UC | AC | Serum | Y, A, O | GC-MS | [14] |
| Histidine | UC | IA | Serum | Y, A, O | GC-MS | [14] |
| Histidine | UC | All | Serum | Y, A, O | GC-MS | [14] |
| Histidine | UC | All | Serum | Y, A, O | GC-MS | [26] |
| Histidine | IBD | All | Urine | A, O | NMR | [16] |
| Homoserine | UC | IA | Serum | Y, A, O | GC-MS | [14] |
| Homoserine | UC | All | Serum | Y, A, O | GC-MS | [14] |
| Hydrogen sulphide | CD | All | Breath | A, O | SIFT-MS | [28] |
| Hydrogen Sulphide | IBD | AC | Breath | Y, A | SIFT-MS | [17] |
| Hydrogen sulphide | IBD | AC | Breath | Y, A | SIFT-MS | [17] |
| Hypoxanthine | CD | AC | Urine | A, O | ^1^H-NMR | [20] |
| Inositol | UC | AC | Serum | Y, A, O | GC-MS | [14] |
| Inositol | UC | IA | Serum | Y, A, O | GC-MS | [14] |
| Inositol | UC | All | Serum | Y, A, O | GC-MS | [14] |
| Inositol, Myo- | UC | IA | Colonic mucosa | Unknown | Proton MRS | [10] |
| Inositol, Myo- | IBD | AC | Colonic mucosa | A | ^1^H-NMR | [27] |
| Inositol, Myo- | CD | AC | Colonic mucosa | Unknown | Proton MRS | [10] |
| Inositol, Myo- | UC | AC | Colonic mucosa | Unknown | Proton MRS | [10] |
| Isocitric acid | UC | All | Rectum tissue | Y, A, O | GC-MS | [26] |
| Isoleucine | UC | AC | Serum | Y, A, O | GC-MS | [14] |
| Isoleucine | UC | IA | Serum | Y, A, O | GC-MS | [14] |
| Isoleucine | UC | All | Serum | Y, A, O | GC-MS | [14] |
| Isoleucine | UC | All | Rectum tissue | Y, A, O | GC-MS | [26] |
| Isoleucine | CD | AC | Colonic mucosa | Unknown | Proton MRS | [10] |
| Isoleucine | UC | AC | Colonic mucosa | Unknown | Proton MRS | [10] |
| Isoprene | CD | AC | Breath | A | GC-tof-MS | [25] |
| Isoprene | CD | IA | Breath | A | GC-tof-MS | [25] |
| KDNα2-3Galβ1-4Glcβ-Cer(d18:1/24:1(15Z))† | UC | All | Plasma | A | LC-MS/MS | [6] |
| KDNα2-3Galβ1-4Glcβ-Cer(d18:1/26:1(17Z))† | UC | All | Plasma | A | LC-MS/MS | [6] |
| Ketoisoleucine_1 | UC | IA | Serum | Y, A, O | GC-MS | [14] |
| Lactate | IBD | AC | Colonic mucosa | A | ^1^H-NMR | [27] |
| Lactate | CD | AC | Colonic mucosa | Unknown | Proton MRS | [10] |
| Lactate | UC | AC | Colonic mucosa | Unknown | Proton MRS | [10] |
| Lactate | UC | IA | Colonic mucosa | Unknown | Proton MRS | [10] |
| Lauric acid | UC | AC | Serum | Y, A, O | GC-MS | [14] |
| Lauric acid | UC | All | Serum | Y, A, O | GC-MS | [14] |
| Leucine | UC | AC | Plasma | A, O | ^1^H-NMR | [20] |
| Leucine | UC | All | Rectum tissue | Y, A, O | GC-MS | [26] |
| Leucine | CD | AC | Colonic mucosa | Unknown | Proton MRS | [10] |
| Leucine | UC | AC | Colonic mucosa | Unknown | Proton MRS | [10] |
| Lipid | UC | AC | Serum | A, O | ^1^H-NMR | [31] |
| Lithocholic acid glycine conjugate | UC | All | Plasma | A | LC-MS/MS | [6] |
| Lysine | UC | All | Rectum tissue | Y, A, O | GC-MS | [26] |
| Lysine | IBD | All | Urine | A, O | NMR | [16] |
| Lysine (4TMS) | UC | IA | Serum | Y, A, O | GC-MS | [14] |
| Lysine (4TMS) | UC | All | Serum | Y, A, O | GC-MS | [14] |
| Malic acid | UC | AC | Serum | Y, A, O | GC-MS | [14] |
| Malic acid | UC | IA | Serum | Y, A, O | GC-MS | [14] |
| Malic acid | UC | All | Serum | Y, A, O | GC-MS | [14] |
| Malic acid | UC | All | Rectum tissue | Y, A, O | GC-MS | [26] |
| Methanethiol | CD | AC | Feces | A, O | GC-MS | [4] |
| Methanethiol | CD | All | Feces | A | GC-MS | [5] |
| Methanethiol | CD | IA | Feces | A, O | GC-tof-MS | [15] |
| Methanethiol | CD | All | Feces | A, O | GC-tof-MS | [15] |
| Methanol | CD | AC | Urine | A, O | ^1^H-NMR | [20] |
| Methanol | CD | All | Urine | Y | ^1^H-NMR | [21] |
| Methanol | UC | AC | Urine | A, O | ^1^H-NMR | [20] |
| Methanol | IBD | All | Urine | A, O | NMR | [16] |
| Methionine | UC | AC | Serum | Y, A, O | GC-MS | [14] |
| Methionine | UC | IA | Serum | Y, A, O | GC-MS | [14] |
| Methionine | UC | All | Serum | Y, A, O | GC-MS | [14] |
| Methionine | UC | All | Rectum tissue | Y, A, O | GC-MS | [26] |
| Methyl 13-sophorosyloxydocosanoate | UC | All | Plasma | A | LC-MS/MS | [6] |
| Methyl acetate | UC | AC | Feces | A, O | GC-MS | [4] |
| Methyl alcohol | CD (sb) | AC | Feces | A, O | GC-MS | [4] |
| Methyl alcohol | CD | All | Feces | A | GC-MS | [5] |
| Methyl alcohol | UC | AC | Feces | A, O | GC-MS | [4] |
| Methyl propyl disulfide | CD | All | Feces | A | GC-MS | [5] |
| Methyl propyl disulfide | CD | IA | Feces | A, O | GC-tof-MS | [15] |
| Methyl propyl disulfide | CD | All | Feces | A, O | GC-tof-MS | [15] |
| Methyl propyl disulfide | UC | IA | Feces | A, O | GC-tof-MS | [15] |
| Methyl propyl disulfide | UC | All | Feces | A, O | GC-tof-MS | [15] |
| Methyl propyl disulfide | IBD | All | Feces | A, O | GC-tof-MS | [15] |
| Methyl propyl disulfide | IBD | IA | Feces | A, O | GC-tof-MS | [15] |
| Methyl-2-propenyl disulfide | IBD | All | Feces | A, O | GC-tof-MS | [15] |
| Methyl-2-propenyl disulfide | IBD | IA | Feces | A, O | GC-tof-MS | [15] |
| Methylamine | CD | Unknown | Feces | Y, A, O | ^1^H-NMR | [24] |
| Methylamine | UC | Unknown | Feces | Y, A, O | ^1^H-NMR | [24] |
| Methylamine | IBD | All | Urine | A, O | NMR | [16] |
| Methylhistidine, pi- | CD | AC | Urine | A, O | ^1^H-NMR | [20] |
| Methylhistidine, tau- | CD | AC | Plasma | A, O | ^1^H-NMR | [20] |
| Methylhistidine, tau- | UC | AC | Serum | A, O | ^1^H-NMR | [20] |
| Methylhistidine, tau- | UC | AC | Plasma | A, O | ^1^H-NMR | [20] |
| Methylstyrene, α- | CD | All | Feces | A | GC-MS | [5] |
| Methylstyrene, α- | CD | IA | Feces | A, O | GC-tof-MS | [15] |
| Methylstyrene, α- | CD | All | Feces | A, O | GC-tof-MS | [15] |
| Methylsuccinate | CD | All | Urine | A | ^1^H-NMR | [22] |
| Methylsuccinate | UC | All | Urine | A | ^1^H-NMR | [22] |
| MGDG (20:5(5Z,8Z,11Z,14Z,17Z)/18:4(6Z,9Z,12Z,15Z)) | UC | All | Plasma | A | LC-MS/MS | [6] |
| Nonanoate | CD | All | Feces | A, O | GC-tof-MS | [15] |
| Nonanoate | UC | All | Feces | A, O | GC-tof-MS | [15] |
| Nonanoate | IBD | All | Feces | A, O | GC-tof-MS | [15] |
| Nonanoic acid | CD | IA | Feces | A, O | GC-tof-MS | [15] |
| Nonanoic acid | UC | IA | Feces | A, O | GC-tof-MS | [15] |
| Nonanoic acid | IBD | IA | Feces | A, O | GC-tof-MS | [15] |
| Nonanoic acid (C9) | UC | AC | Serum | Y, A, O | GC-MS | [14] |
| Nonanoic acid (C9) | UC | IA | Serum | Y, A, O | GC-MS | [14] |
| Nonanoic acid (C9) | UC | All | Serum | Y, A, O | GC-MS | [14] |
| Nonanoyl-CoA | UC | All | Plasma | A | LC-MS/MS | [6] |
| Nonyl cyclopropane | IBD | All | Feces | A, O | GC-tof-MS | [15] |
| Nonyl cyclopropane | IBD | IA | Feces | A, O | GC-tof-MS | [15] |
| Octanoate | CD | AC | Feces | A | GC-MS | [5] |
| Octanoate | CD | All | Feces | A | GC-MS | [5] |
| Octanoate | CD | All | Feces | A, O | GC-tof-MS | [15] |
| Octanoate | UC | All | Feces | A, O | GC-tof-MS | [15] |
| Octanoate | IBD | All | Feces | A, O | GC-tof-MS | [15] |
| Octanoic acid | CD | IA | Feces | A, O | GC-tof-MS | [15] |
| Octanoic acid | UC | IA | Feces | A, O | GC-tof-MS | [15] |
| Octanoic acid | IBD | IA | Feces | A, O | GC-tof-MS | [15] |
| Ornithine | CD | AC | Plasma | A, O | ^1^H-NMR | [20] |
| Ornithine | UC | AC | Urine | A, O | ^1^H-NMR | [20] |
| Ornithine | UC | IA | Serum | Y, A, O | GC-MS | [14] |
| Ornithine | UC | All | Serum | Y, A, O | GC-MS | [14] |
| Oxalate | UC | AC | Serum | Y, A, O | GC-MS | [14] |
| Oxalate | UC | All | Serum | Y, A, O | GC-MS | [14] |
| Palmitoleate | UC | AC | Serum | Y, A, O | GC-MS | [14] |
| Palmitoleate | UC | IA | Serum | Y, A, O | GC-MS | [14] |
| Palmitoleate | UC | All | Serum | Y, A, O | GC-MS | [14] |
| PC (16:1(9Z)/2:0) | UC | All | Plasma | A | LC-MS/MS | [6] |
| PC (18:2(9Z,12Z)/2:0)[U] | UC | All | Plasma | A | LC-MS/MS | [6] |
| PC (8:2(2E,4E)/8:2(2E,4E)) | CD | All | Plasma | A | LC-MS/MS | [6] |
| Pentanoate | CD | AC | Feces | A | GC-MS | [5] |
| Pentanoate | CD | All | Feces | A | GC-MS | [5] |
| Pentanoate | CD | All | Feces | A, O | GC-tof-MS | [15] |
| Pentanoate | UC | All | Feces | A, O | GC-tof-MS | [15] |
| Pentanoate | IBD | All | Feces | A, O | GC-tof-MS | [15] |
| Pentanoic acid | CD | IA | Feces | A, O | GC-tof-MS | [15] |
| Pentanoic acid | UC | IA | Feces | A, O | GC-tof-MS | [15] |
| Pentanoic acid | IBD | IA | Feces | A, O | GC-tof-MS | [15] |
| PGPC | UC | All | Plasma | A | LC-MS/MS | [6] |
| Phellandrene, α- | UC | AC | Feces | A, O | GC-MS | [4] |
| Phellandrene, α- | CD | IA | Feces | A, O | GC-tof-MS | [15] |
| Phellandrene, α- | CD | All | Feces | A, O | GC-tof-MS | [15] |
| Phellandrene, α- | UC | IA | Feces | A, O | GC-tof-MS | [15] |
| Phellandrene, α- | UC | All | Feces | A, O | GC-tof-MS | [15] |
| Phenol, 4-ethyl | CD (sb) | AC | Feces | A, O | GC-MS | [4] |
| Phenylalanine | UC | AC | Urine | A, O | ^1^H-NMR | [20] |
| Phenylalanine | UC | IA | Serum | Y, A, O | GC-MS | [14] |
| Phenylalanine | UC | All | Serum | Y, A, O | GC-MS | [14] |
| Phenylalanine | UC | All | Rectum tissue | Y, A, O | GC-MS | [26] |
| Phosphate | UC | AC | Serum | Y, A, O | GC-MS | [14] |
| Phosphate | UC | All | Serum | Y, A, O | GC-MS | [14] |
| Phosphate | UC | IA | Serum | Y, A, O | GC-MS | [14] |
| Phosphoethanolamine, O- | UC | AC | Serum | Y, A, O | GC-MS | [14] |
| Phosphoethanolamine, O- | UC | IA | Serum | Y, A, O | GC-MS | [14] |
| Phosphoethanolamine, O- | UC | All | Serum | Y, A, O | GC-MS | [14] |
| Phosphatidylinositol 16:0/18:1 | CD | All | Ileum | A, O | ESI-MS | [2] |
| Pinene, α- | CD | AC | Feces | A, O | GC-MS | [4] |
| Pinene, α- | CD (sb) | AC | Feces | A, O | GC-MS | [4] |
| Pinene, α- | UC | AC | Feces | A, O | GC-MS | [4] |
| Proline | UC | AC | Serum | Y, A, O | GC-MS | [14] |
| Proline | UC | All | Serum | Y, A, O | GC-MS | [14] |
| Proline | UC | All | Rectum tissue | Y, A, O | GC-MS | [26] |
| Propanal | UC | IA | Feces | A, O | GC-tof-MS | [15] |
| Propanal | UC | All | Feces | A, O | GC-tof-MS | [15] |
| Propionate | CD | AC | Feces | A, O | ^1^H-NMR | [1] |
| Propionate | UC | All | Feces | A | GC-MS | [13] |
| Prostaglandin F1a | CD (CCD) | IA | Feces | Y, A, O | FT-ICR-MS | [32] |
| Prostaglandin F1a | CD (ICD) | IA | Feces | Y, A, O | FT-ICR-MS | [32] |
| Proteacin | UC | All | Plasma | A | LC-MS/MS | [6] |
| PS (18:0/22:5(7Z,10Z,13Z,16Z,19Z)) | UC | All | Plasma | A | LC-MS/MS | [6] |
| Sarcosine | UC | AC | Serum | Y, A, O | GC-MS | [14] |
| Sarcosine | UC | All | Serum | Y, A, O | GC-MS | [14] |
| Sebacic acid | UC | IA | Serum | Y, A, O | GC-MS | [14] |
| Serine | UC | AC | Serum | A, O | ^1^H-NMR | [20] |
| Serine | UC | AC | Urine | A, O | ^1^H-NMR | [20] |
| Serine | UC | All | Rectum tissue | Y, A, O | GC-MS | [26] |
| Serine (3TMS) | UC | AC | Serum | Y, A, O | GC-MS | [14] |
| Serine (3TMS) | UC | IA | Serum | Y, A, O | GC-MS | [14] |
| Serine (3TMS) | UC | All | Serum | Y, A, O | GC-MS | [14] |
| Stercobilin | CD | IA | Feces | Unknown | UPLC/tof-MS | [12] |
| Succinate | CD | AC | Plasma | A, O | ^1^H-NMR | [20] |
| Succinate | CD | AC | Urine | A, O | ^1^H-NMR | [20] |
| Succinate | UC | AC | Urine | A, O | ^1^H-NMR | [20] |
| Succinate | UC | All | Urine | Y | ^1^H-NMR | [21] |
| Succinate | IBD | AC | Urine | A, O | ^1^H-NMR | [18] |
| Succinate | IBD | IA | Urine | A, O | ^1^H-NMR | [18] |
| Succinate | IBD | All | Urine | A, O | NMR | [16] |
| Succinate | CD | AC | Colonic mucosa | Unknown | Proton MRS | [10] |
| Succinate | UC | AC | Colonic mucosa | Unknown | Proton MRS | [10] |
| Succinic acid | UC | All | Rectum tissue | Y, A, O | GC-MS | [26] |
| Succinic acid (or aldehyde) | UC | AC | Serum | Y, A, O | GC-MS | [14] |
| Succinic acid (or aldehyde) | UC | IA | Serum | Y, A, O | GC-MS | [14] |
| Succinic acid (or aldehyde) | UC | All | Serum | Y, A, O | GC-MS | [14] |
| Tagatose_2 (or psicose_2) | UC | AC | Serum | Y, A, O | GC-MS | [14] |
| Tagatose_2 (or psicose_2) | UC | IA | Serum | Y, A, O | GC-MS | [14] |
| Tagatose_2 (or psicose_2) | UC | All | Serum | Y, A, O | GC-MS | [14] |
| Taurine | CD | AC | Urine | A, O | ^1^H-NMR | [20] |
| Taurine | CD | All | Urine | Y | ^1^H-NMR | [21] |
| Taurine | UC | All | Urine | Y | ^1^H-NMR | [21] |
| Taurine | IBD | AC | Urine | A, O | ^1^H-NMR | [18] |
| Taurine | IBD | IA | Urine | A, O | ^1^H-NMR | [18] |
| Taurine | UC | AC | Serum | Y, A, O | GC-MS | [14] |
| Taurine | UC | IA | Serum | Y, A, O | GC-MS | [14] |
| Taurine | UC | All | Serum | Y, A, O | GC-MS | [14] |
| Taurine | IBD | All | Urine | A, O | NMR | [16] |
| TG (20:5(5Z,8Z,11Z,14Z,17Z)/22:6(4Z,7Z,10Z,13Z,16Z,19Z)/20:5(5Z,8Z,11Z,14Z,17Z))(d5) | UC | All | Plasma | A | LC-MS/MS | [6] |
| Theanine_2 | UC | IA | Serum | Y, A, O | GC-MS | [14] |
| Theanine_2 | UC | All | Serum | Y, A, O | GC-MS | [14] |
| Threonine | UC | All | Rectum tissue | Y, A, O | GC-MS | [26] |
| Threonine (2TMS) | UC | IA | Serum | Y, A, O | GC-MS | [14] |
| Threonine (2TMS) | UC | All | Serum | Y, A, O | GC-MS | [14] |
| Trignoelline | IBD | All | Urine | A, O | NMR | [16] |
| Trigonelline | CD | AC | Urine | A, O | ^1^H-NMR | [20] |
| Trigonelline | CD | All | Urine | A | ^1^H-NMR | [22] |
| Trigonelline | UC | All | Urine | A | ^1^H-NMR | [22] |
| Trigonelline | IBD | AC | Urine | A, O | ^1^H-NMR | [18] |
| Trimethylamine | CD | Unknown | Feces | Y, A, O | ^1^H-NMR | [24] |
| Trimethylamine | UC | Unknown | Feces | Y, A, O | ^1^H-NMR | [24] |
| Tryptophan | UC | All | Serum | Y, A, O | GC-MS | [26] |
| Tryptophan | CD | All | Serum | Y, A, O | GC-MS | [26] |
| Tryptophan | UC | AC | Serum | Y, A, O | GC-MS | [14] |
| Tryptophan | UC | IA | Serum | Y, A, O | GC-MS | [14] |
| Tryptophan | UC | All | Serum | Y, A, O | GC-MS | [14] |
| Tyrosine | CD | AC | Plasma | A, O | ^1^H-NMR | [20] |
| Tyrosine | UC | AC | Serum | A, O | ^1^H-NMR | [20] |
| Tyrosine | UC | AC | Plasma | A, O | ^1^H-NMR | [20] |
| Tyrosine | UC | AC | Serum | Y, A, O | GC-MS | [14] |
| Tyrosine | UC | IA | Serum | Y, A, O | GC-MS | [14] |
| Tyrosine | UC | All | Serum | Y, A, O | GC-MS | [14] |
| Tyrosine | UC | All | Rectum tissue | Y, A, O | GC-MS | [26] |
| Ubiquinone 8 | UC | All | Plasma | A | LC-MS/MS | [6] |
| Unassigned metabolite 1 | CD | All | Urine | Y | ^1^H-NMR | [21] |
| Unknown (1.33) | CD | Unknown | Feces | Y, A, O | ^1^H-NMR | [24] |
| Unknown (1.33) | UC | Unknown | Feces | Y, A, O | ^1^H-NMR | [24] |
| Unknown1 | UC | All | Feces | A, O | ^1^H-NMR | [19] |
| Unknown2 | UC | All | Feces | A, O | ^1^H-NMR | [19] |
| Urea | CD | AC | Serum | A, O | ^1^H-NMR | [20] |
| Urea | CD | AC | Plasma | A, O | ^1^H-NMR | [20] |
| Urea | CD | AC | Urine | A, O | ^1^H-NMR | [20] |
| Urea | CD | All | Urine | Y | ^1^H-NMR | [21] |
| Urea | CD | All | Urine | Y | ^1^H-NMR | [21] |
| Urea | CD | All | Urine | Y | ^1^H-NMR | [21] |
| Urea | CD | All | Urine | Y | ^1^H-NMR | [21] |
| Urea | UC | AC | Serum | A, O | ^1^H-NMR | [20] |
| Urea | UC | AC | Plasma | A, O | ^1^H-NMR | [20] |
| Urea | UC | AC | Urine | A, O | ^1^H-NMR | [20] |
| Urea | UC | All | Urine | Y | ^1^H-NMR | [21] |
| Urea | IBD | All | Urine | A, O | NMR | [16] |
| Uric acid | UC | AC | Serum | Y, A, O | GC-MS | [14] |
| Uric acid | UC | IA | Serum | Y, A, O | GC-MS | [14] |
| Uric acid | UC | All | Serum | Y, A, O | GC-MS | [14] |
| Valine | CD | AC | Serum | A | ^1^H-NMR | [33] |
| Valine | CD | AC | Serum | A, O | ^1^H-NMR | [20] |
| Valine | CD | AC | Plasma | A, O | ^1^H-NMR | [20] |
| Valine | UC | AC | Serum | A, O | ^1^H-NMR | [20] |
| Valine | UC | AC | Plasma | A, O | ^1^H-NMR | [20] |
| Valine | UC | All | Rectum tissue | Y, A, O | GC-MS | [26] |
| Valine | CD | AC | Colonic mucosa | Unknown | Proton MRS | [10] |
| Valine | UC | AC | Colonic mucosa | Unknown | Proton MRS | [10] |
| Valine (2TMS) | UC | AC | Serum | Y, A, O | GC-MS | [14] |
| Valine (2TMS) | UC | IA | Serum | Y, A, O | GC-MS | [14] |
| Valine (2TMS) | UC | All | Serum | Y, A, O | GC-MS | [14] |
| VOC1 (RT = 12.2 min) | CD | IA | Breath | A | GC-tof-MS | [25] |
| VOC1 (RT = 12.2 min) | CD | AC | Breath | A | GC-tof-MS | [25] |
| Xanthine | UC | AC | Serum | Y, A, O | GC-MS | [14] |
| Xanthine | UC | IA | Serum | Y, A, O | GC-MS | [14] |
| Xanthine | UC | All | Serum | Y, A, O | GC-MS | [14] |
| Xylene, o- | UC | AC | Feces | A, O | GC-MS | [4] |
| Xylitol | UC | AC | Serum | Y, A, O | GC-MS | [14] |

‘-‘ indicates that no metabolites were found to be significantly increased in the respective sample.

Disease: CD: Crohn’s disease; CCD: colonic CD; IBD: inflammatory bowel disease; ICD: ileal CD; sb: small bowel; UC: ulcerative colitis. Activity: AC: active; IA: inactive; All: active + inactive. Age groups: Y: very early onset and young; A: adult; O: old. Platform: ESI-MS: electrospray ionization mass spectrometry; FT-ICR-MS: Fourier-transform ion cyclotron resonance mass spectrometry; GC-MS: gas chromatography-mass spectrometry; GC-tof-MS: gas chromatography time-of-flight mass spectrometry; (HP)LC-MS: high performance liquid chromatography-mass spectrometry; LC-ESI-MS/MS : liquid chromatography electrospray ionization tandem mass spectrometry; LC-MS/MS : liquid chromatography tandem mass spectrometry; MRS: magnetic resonance spectroscopy; NMR: nuclear magnetic resonance; SIFT-MS: selected-ion flow-tube mass spectrometry; UPLC/ToFMS: ultra performance liquid chromatography time-of-flight mass spectrometry. MGDG: monogalactosyldiacylglycerol; PBMC: peripheral blood mononuclear cells; PC: phosphatidylcholine; PGPC: 1-palmitoyl-2-glutaryl phosphatidylcholine; PS: phosphatidylserine; TG: triglyceride.

*O-(N-acetyl-a-neuraminosyl)-(2->3)-O-b-D-galactopyranosyl-(1->4)-O-2-(acetylamino)-2-deoxy-b-D-glucopyranosyl-(1->3)-O-b-D-galactopyranosyl-(1->4)-O-2-(acetylamino)-2-deoxy-b-D-glucopyranosyl-(1->3)-O-b-D-galactopyranosyl-(1->4)-D-Gluc.

**References**

1. Bjerrum, J.T.; Wang, Y.; Hao, F.; Coskun, M.; Ludwig, C.; Gunther, U.; Nielsen, O.H. Metabonomics of human fecal extracts characterize ulcerative colitis, Crohn's disease and healthy individuals. *Metabolomics : Official journal of the Metabolomic Society* **2015**, *11*, 122-133, doi:10.1007/s11306-014-0677-3.

2. Sewell, G.W.; Hannun, Y.A.; Han, X.; Koster, G.; Bielawski, J.; Goss, V.; Smith, P.J.; Rahman, F.Z.; Vega, R.; Bloom, S.L., et al. Lipidomic profiling in Crohn's disease: abnormalities in phosphatidylinositols, with preservation of ceramide, phosphatidylcholine and phosphatidylserine composition. *The international journal of biochemistry & cell biology* **2012**, *44*, 1839-1846, doi:10.1016/j.biocel.2012.06.016.

3. Cracowski, J.L.; Bonaz, B.; Bessard, G.; Bessard, J.; Anglade, C.; Fournet, J. Increased urinary F2-isoprostanes in patients with Crohn's disease. *American Journal of Gastroenterology* **2002**, *97*, 99-103.

4. Ahmed, I.; Greenwood, R.; Costello, B.; Ratcliffe, N.; Probert, C.S. Investigation of faecal volatile organic metabolites as novel diagnostic biomarkers in inflammatory bowel disease. *Alimentary Pharmacology and Therapeutics* **2016**, *43*, 596-611.

5. De Preter, V.; Joossens, M.; Ballet, V.; Shkedy, Z.; Rutgeerts, P.; Vermeire, S.; Verbeke Phd, K. Metabolic profiling of the impact of oligofructose-enriched inulin in Crohn's disease patients: a double-blinded randomized controlled trial. *Clinical and translational gastroenterology* **2013**, *4*, e30, doi:10.1038/ctg.2012.24.

6. Yau, Y.Y.; Leong, R.W.L.; Shin, S.; Bustamante, S.; Pickford, R.; Hejazi, L.; Campbell, B.; Wasinger, V.C. Bimodal plasma metabolomics strategy identifies novel inflammatory metabolites in inflammatory bowel diseases. *Discovery medicine* **2014**, *18*, 113-124.

7. Thyssen, E.; Turk, J.; Bohrer, A.; Stenson, W.F. Quantification of distinct molecular species of platelet activating factor in ulcerative colitis. *Lipids* **1996**, *31*, S255-S259.

8. Iwamoto, J.; Saito, Y.; Honda, A.; Miyazaki, T.; Ikegami, T.; Matsuzaki, Y. Bile acid malabsorption deactivates pregnane x receptor in patients with Crohn's Disease. *Inflammatory bowel diseases* **2013**, *19*, 1278-1284.

9. Johnson, J.C.; Schmidt, C.R.; Shrubsole, M.J.; Billheimer, D.D.; Joshi, P.R.; Morrow, J.D.; Heslin, M.J.; Washington, M.K.; Ness, R.M.; Zheng, W., et al. Urine PGE-M: A Metabolite of Prostaglandin E2 as a Potential Biomarker of Advanced Colorectal Neoplasia. *Clinical Gastroenterology and Hepatology* **2006**, *4*, 1358-1365.

10. Balasubramanian, K.; Kumar, S.; Singh, R.R.; Sharma, U.; Ahuja, V.; Makharia, G.K.; Jagannathan, N.R. Metabolism of the colonic mucosa in patients with inflammatory bowel diseases: an in vitro proton magnetic resonance spectroscopy study. *Magnetic Resonance Imaging* **2009**, *27*, 79-86.

11. Rieder, F.; Kurada, S.; Grove, D.; Cikach, F.; Lopez, R.; Patel, N.; Singh, A.; Alkhouri, N.; Shen, B.; Brzezinski, A., et al. A Distinct Colon-Derived Breath Metabolome is Associated with Inflammatory Bowel Disease, but not its Complications. *Clinical and translational gastroenterology* **2016**, *7*, e201, doi:10.1038/ctg.2016.57.

12. Jacobs, J.P.; Goudarzi, M.; Singh, N.; Tong, M.; McHardy, I.H.; Ruegger, P.; Asadourian, M.; Moon, B.H.; Ayson, A.; Borneman, J., et al. A Disease-Associated Microbial and Metabolomics State in Relatives of Pediatric Inflammatory Bowel Disease Patients. *Cellular and molecular gastroenterology and hepatology* **2016**, *2*, 750-766.

13. Machiels, K.; Joossens, M.; Sabino, J.; De Preter, V.; Arijs, I.; Eeckhaut, V.; Ballet, V.; Claes, K.; Van Immerseel, F.; Verbeke, K., et al. A decrease of the butyrate-producing species roseburia hominis and faecalibacterium prausnitzii defines dysbiosis in patients with ulcerative colitis. *Gut* **2014**, *63*, 1275-1283.

14. Kohashi, M.; Nishiumi, S.; Ooi, M.; Yoshie, T.; Matsubara, A.; Suzuki, M.; Hoshi, N.; Kamikozuru, K.; Yokoyama, Y.; Fukunaga, K., et al. A novel gas chromatography mass spectrometry-based serum diagnostic and assessment approach to ulcerative colitis. *Journal of Crohn's and Colitis* **2014**, *8*, 1010-1021.

15. De Preter, V.; Machiels, K.; Joossens, M.; Arijs, I.; Matthys, C.; Vermeire, S.; Rutgeerts, P.; Verbeke, K. Faecal metabolite profiling identifies medium-chain fatty acids as discriminating compounds in IBD. *Gut* **2015**, *64*, 447-458.

16. Stephens, N.S.; Siffledeen, J.; Su, X.; Murdoch, T.B.; Fedorak, R.N.; Slupsky, C.M. Urinary NMR metabolomic profiles discriminate inflammatory bowel disease from healthy. *Journal of Crohn's and Colitis* **2013**, *7*, e42-e48.

17. Patel, N.; Alkhouri, N.; Eng, K.; Cikach, F.; Mahajan, L.; Yan, C.; Grove, D.; Rome, E.S.; Lopez, R.; Dweik, R.A. Metabolomic analysis of breath volatile organic compounds reveals unique breathprints in children with inflammatory bowel disease: A pilot study. *Alimentary Pharmacology and Therapeutics* **2014**, *40*, 498-507.

18. Dawiskiba, T.; Deja, S.; Mulak, A.; Zabek, A.; Jawien, E.; Pawelka, D.; Banasik, M.; Mastalerz-Migas, A.; Balcerzak, W.; Kaliszewski, K., et al. Serum and urine metabolomic fingerprinting in diagnostics of inflammatory bowel diseases. *World journal of gastroenterology : WJG* **2014**, *20*, 163-174, doi:10.3748/wjg.v20.i1.163.

19. Le Gall, G.; Noor, S.O.; Ridgway, K.; Scovell, L.; Jamieson, C.; Johnson, I.T.; Colquhoun, I.J.; Kemsley, E.K.; Narbad, A. Metabolomics of fecal extracts detects altered metabolic activity of gut microbiota in ulcerative colitis and irritable bowel syndrome. *Journal of proteome research* **2011**, *10*, 4208-4218.

20. Schicho, R.; Shaykhutdinov, R.; Ngo, J.; Nazyrova, A.; Schneider, C.; Panaccione, R.; Kaplan, G.G.; Vogel, H.J.; Storr, M. Quantitative metabolomic profiling of serum, plasma, and urine by 1H NMR spectroscopy discriminates between patients with inflammatory bowel disease and healthy individuals. *Journal of proteome research* **2012**, *11*, 3344-3357.

21. Martin, F.P.; Ezri, J.; Cominetti, O.; Da Silva, L.; Kussmann, M.; Godin, J.P.; Nydegger, A. Urinary metabolic phenotyping reveals differences in the metabolic status of healthy and inflammatory bowel disease (IBD) children in relation to growth and disease activity. *International journal of molecular sciences* **2016**, *17*, no pagination.

22. Alonso, A.; Julia, A.; Vinaixa, M.; Domenech, E.; Fernandez-Nebro, A.; Canete, J.D.; Ferrandiz, C.; Tornero, J.; Gisbert, J.P.; Nos, P., et al. Urine metabolome profiling of immune-mediated inflammatory diseases. *BMC medicine* **2016**, *14*, 133, doi:10.1186/s12916-016-0681-8.

23. Williams, H.R.T.; Cox, I.J.; Walker, D.G.; North, B.V.; Patel, V.M.; Marshall, S.E.; Jewell, D.P.; Ghosh, S.; Thomas, H.J.W.; Teare, J.P., et al. Characterization of inflammatory bowel disease with urinary metabolic profiling. *American Journal of Gastroenterology* **2009**, *104*, 1435-1444.

24. Marchesi, J.R.; Holmes, E.; Khan, F.; Kochhar, S.; Scanlan, P.; Shanahan, F.; Wilson, I.D.; Wang, Y. Rapid and noninvasive metabonomic characterization of inflammatory bowel disease. *Journal of proteome research* **2007**, *6*, 546-551.

25. Bodelier, A.G.L.; Smolinska, A.; Baranska, A.; Dallinga, J.W.; Mujagic, Z.; Vanhees, K.; Van Den Heuvel, T.; Masclee, A.A.M.; Jonkers, D.; Pierik, M.J., et al. Volatile organic compounds in exhaled air as novel marker for disease activity in Crohn's disease: A metabolomic approach. *Inflammatory bowel diseases* **2015**, *21*, 1776-1785.

26. Ooi, M.; Nishiumi, S.; Yoshie, T.; Shiomi, Y.; Kohashi, M.; Fukunaga, K.; Nakamura, S.; Matsumoto, T.; Hatano, N.; Shinohara, M., et al. GC/MS-based profiling of amino acids and TCA cycle-related molecules in ulcerative colitis. *Inflammation Research* **2011**, *60*, 831-840.

27. Sharma, U.; Singh, R.R.; Ahuja, V.; Makharia, G.K.; Jagannathan, N.R. Similarity in the metabolic profile in macroscopically involved and un-involved colonic mucosa in patients with inflammatory bowel disease: An in vitro proton (1H) MR spectroscopy study. *Magnetic Resonance Imaging* **2010**, *28*, 1022-1029.

28. Hicks, L.C.; Huang, J.; Kumar, S.; Powles, S.T.; Orchard, T.R.; Hanna, G.B.; Williams, H.R. Analysis of Exhaled Breath Volatile Organic Compounds in Inflammatory Bowel Disease: A Pilot Study. *Journal of Crohn's & colitis* **2015**, *9*, 731-737.

29. Shiomi, Y.; Nishiumi, S.; Ooi, M.; Hatano, N.; Shinohara, M.; Yoshie, T.; Kondo, Y.; Furumatsu, K.; Shiomi, H.; Kutsumi, H., et al. GCMS-based metabolomic study in mice with colitis induced by dextran sulfate sodium. *Inflammatory bowel diseases* **2011**, *17*, 2261-2274.

30. Williams, H.R.; Cox, I.J.; Walker, D.G.; Cobbold, J.F.; Taylor-Robinson, S.D.; Marshall, S.E.; Orchard, T. Differences in gut microbial metabolism are responsible for reduced hippurate synthesis in Crohn's disease. *Gastroenterology* **2010**, *138*, S579.

31. Zhang, Y.; Lin, L.; Xu, Y.; Lin, Y.; Jin, Y.; Zheng, C. 1H NMR-based spectroscopy detects metabolic alterations in serum of patients with early-stage ulcerative colitis. *Biochemical and biophysical research communications* **2013**, *433*, 547-551.

32. Jansson, J.; Willing, B.; Lucio, M.; Fekete, A.; Dicksved, J.; Halfvarson, J.; Tysk, C.; Schmitt-Kopplin, P. Metabolomics reveals metabolic biomarkers of Crohn's disease. *PloS one* **2009**, *4*, e6386, doi:10.1371/journal.pone.0006386.

33. Fathi, F.; Majari-Kasmaee, L.; Mani-Varnosfaderani, A.; Kyani, A.; Rostami-Nejad, M.; Sohrabzadeh, K.; Naderi, N.; Zali, M.R.; Rezaei-Tavirani, M.; Tafazzoli, M., et al. 1H NMR based metabolic profiling in Crohn's disease by random forest methodology. *Magnetic resonance in chemistry : MRC* **2014**, *52*, 370-376.
