# Supplementary material for: Translational Potential of Metabolomics on Animal Models of Inflammatory Bowel Disease—A Systematic Critical Review
Source: Int J Mol Sci. 2020 May 29;21(11):3856. doi: 10.3390/ijms21113856 (PMC7312423; doi:10.3390/ijms21113856)
Supplement: Supplementary file 1 [file ijms-21-03856-s001.zip › Supplementary Table S7_resubmission_proofread.docx]

**Supplementary Table S7: Metabolites significantly increased in IBD vs healthy controls in animal models**

| **Metabolites ↑ in IBD** | **Model** | **Species** | **Sample type** | **Age group** | **Platform** | **Reference** |
| --- | --- | --- | --- | --- | --- | --- |
| - | Adoptive transfer | Mouse | Urine | >8-24 | ^1^H-NMR | [1] |
| - | Adoptive transfer | Mouse | Plasma | >8-24 | ^1^H-NMR | [1] |
| - | DSS (A) | Rat | Plasma | >3-8 | LC-MS | [2] |
| - | DSS (A) | Rat | Plasma | >8-24 | LC-MS | [2] |
| - | DSS (C) | Rat | Plasma | >8-24 | LC-MS | [2] |
| - | *TNF*^ΔARE/WT^ | Mouse | Ileum (distal) | >3-8 | LC-MS | [3] |
| - | *IL10^-/-^* | Mouse | Urine | >3-8 | NMR | [4] |
| - | *IL10^-/-^* | Mouse | Urine | >3-8 | NMR | [4] |
| - | DSS (A) | Piglet | Distal colon | 0-3 | UHPLC/MS-MS | [5] |
| - | DSS (A) | Piglet | Red blood cells | 0-3 | UHPLC/MS-MS | [5] |
| - | DSS (A) | Piglet | Liver | 0-3 | UHPLC/MS-MS | [5] |
| - | DSS (A) | Piglet | Spiral colon | 0-3 | UHPLC/MS-MS | [5] |
| - | DSS (A) | Piglet | Small intestine | 0-3 | UHPLC/MS-MS | [5] |
| - | DSS (A) | Piglet | Longissimus dorsi | 0-3 | UHPLC/MS-MS | [5] |
| - | DSS (A) | Piglet | Masseter | 0-3 | UHPLC/MS-MS | [5] |
| - | DSS (A) | Mouse | Colon | >3-24 | UPLC-ESI-qTOFMS | [6] |
| - | DSS (A) | Mouse | Colon | >8-24 | UPLC-MS | [7] |
| (2)-Perillyl alcohol | *Winnie* | Mouse | Feces | >8-24 | GC-MS | [8] |
| (3-Carboxypropyl)trimethylammonium chloride | *Winnie* | Mouse | Feces | >8-24 | GC-MS | [8] |
| 1,1-diethoxypropane, 3-(trimethylsilyl)- | *Winnie* | Mouse | Feces | >8-24 | GC-MS | [8] |
| 11,12-Dihydroxy eicosatrienoic acid | DSS (C) | Rat | Plasma | >8-24 | LC-MS | [2] |
| 11,12-Dihydroxy eicosatrienoic acid | *TNF*^ΔARE/WT^ | Mouse | Ileum (distal) | >8-24 | LC-MS | [3] |
| 12,13-Dihydroxy octadecenoic acid | *IL10^-/-^* | Mouse | Plasma | >24 | LC/MS-MS | [9] |
| 12,13-Dihydroxy octadecenoic acid | *IL10^-/-^* | Mouse | Plasma | >24 | LC/MS-MS | [9] |
| 14,15-Dihydroxy eicosatrienoic acid | DSS (A) | Rat | Plasma | >8-24 | LC-MS | [2] |
| 14,15-Dihydroxy eicosatrienoic acid | DSS (C) | Rat | Plasma | >8-24 | LC-MS | [2] |
| 14,15-Dihydroxyeicosatrienoic acid | *IL10^-/-^* | Mouse | Plasma | >24 | LC/MS-MS | [9] |
| 14,15-Dihydroxyeicosatrienoic acid | *IL10^-/-^* | Mouse | Plasma | >24 | LC/MS-MS | [9] |
| 17-Hydroxydocosahexaenoic acid | DSS (A) | Mouse | Colon | >3-8 | HPLC-MS/MS | [10] |
| 1-Hexadecanol | T-syn deficiency | Mouse | Colon (distal), cecum | >8-24 | UPLC/ToF-MS | [11] |
| 1-Methylhistamine | H. hepaticus | Mouse | Serum | >24 | UPLC-ESI-TOF-MS | [12] |
| 1-Naphtylamine | T-syn deficiency | Mouse | Colon (distal), cecum | >8-24 | UPLC/ToF-MS | [11] |
| 1-O-Methyl-α-D-galactoside | *Winnie* | Mouse | Feces | >8-24 | GC-MS | [8] |
| 1-Phenyl-1-(2-trimethylsilylcyclopentyl)ethanol | *Winnie* | Mouse | Feces | >8-24 | GC-MS | [8] |
| 2,3-Butanediol diTMS, (+/2)- | *Winnie* | Mouse | Feces | >8-24 | GC-MS | [8] |
| 2,3-Dinor-8-iso prostaglandin F1α | T-syn deficiency | Mouse | Colon (distal), cecum | >3-8 | UPLC/ToF-MS | [11] |
| 20-Hydroxy eicosatetraenoic acid | DSS (A) | Rat | Plasma | >8-24 | LC-MS | [2] |
| 2-Hydroxyisocaproic acid | DSS (A) | Mouse | Serum | >8-24 | GC-MS | [13] |
| 2-Hydroxypyridine | *Winnie* | Mouse | Feces | >8-24 | GC-MS | [8] |
| 2-Methyl-3-hydroxybutyric acid | DSS (A) | Mouse | Serum | >8-24 | GC-MS | [13] |
| 2-Methyl-3-hydroxybutyric acid | DSS (A) | Mouse | Serum | >8-24 | GC-MS | [13] |
| 2-Methyl-4-phenyl-4-trimethylsilylbutan-2-ol | *Winnie* | Mouse | Feces | >8-24 | GC-MS | [8] |
| 2-Methylbutyroylcarnitine | DSS (C) | Mouse | Colon | >8-24 | LC-qTOF-MS | [14] |
| 2-Oxoglutarate | DSS (A) | Mouse | Urine | >8-24 | ^1^H-NMR | [15] |
| 2-Oxoglutarate | DSS (A) | Mouse | Urine | >8-24 | ^1^H-NMR | [15] |
| 2-Oxoglutarate | DSS (A) | Mouse | Urine | >8-24 | ^1^H-NMR | [15] |
| 2-Oxoglutarate | DSS (A) | Mouse | Urine | >8-24 | ^1^H-NMR | [15] |
| 2-Oxoglutarate | DSS (A) | Mouse | Urine | >8-24 | ^1^H-NMR | [15] |
| 2-Oxoglutarate | DSS (A) | Mouse | Urine | >8-24 | ^1^H-NMR | [15] |
| 2-Pyrrolidone-5-carboxylic acid, (R)-(+)- | DSS (C) | Mouse | Colon | >8-24 | LC-qTOF-MS | [14] |
| 3,4-Dihydroxybutyric acid | *IL10^-/-^* | Mouse | Urine | >3-8 | GC-MS | [16] |
| 3,4-Dihydroxybutyric acid | *IL10^-/-^* | Mouse | Urine | >3-8 | GC-MS | [16] |
| 3,4-Dihydroxybutyric acid | *IL10^-/-^* | Mouse | Urine | >3-8 | GC-MS | [16] |
| 3,4-Dihydroxybutyric acid | *IL10^-/-^* | Mouse | Urine | >8-24 | GC-MS | [16] |
| 3,4-Dihydroxybutyric acid | *IL10^-/-^* | Mouse | Urine | >8-24 | GC-MS | [16] |
| 3,4-Dihydroxybutyric acid | *IL10^-/-^* | Mouse | Urine | >3-8 | GC-MS | [16] |
| 3,4-Dihydroxybutyric acid | *IL10^-/-^* | Mouse | Urine | >8-24 | GC-MS | [16] |
| 3,4-Dihydroxymandelic acid | DSS (A) | Mouse | Colon | >8-24 | GC-MS | [13] |
| 3-Hydroxyanthranilic acid | *IL10^-/-^* | Mouse | Plasma | >8-24 | LC-MS | [16] |
| 3-Hydroxybutyrate | DSS (A) | Mouse | Serum | >3-8 | ^1^H-NMR | [17] |
| 3-Hydroxybutyric acid | DSS (A) | Mouse | Serum | >8-24 | GC-MS | [13] |
| 3-Hydroxybutyric acid | DSS (A) | Mouse | Serum | >8-24 | GC-MS | [13] |
| 3-Hydroxykynurenine | *IL10^-/-^* | Mouse | Plasma | >8-24 | LC-MS | [16] |
| 3-Hydroxykynurenine | *IL10^-/-^* | Mouse | Plasma | >8-24 | LC-MS | [16] |
| 3-Hydroxypropionic acid | DSS (A) | Mouse | Serum | >8-24 | GC-MS | [13] |
| 3-Hydroxypropionic acid | DSS (A) | Mouse | Colon | >8-24 | GC-MS | [13] |
| 3-Hydroxypropionic acid | DSS (A) | Mouse | Serum | >8-24 | GC-MS | [13] |
| 3-Methyl-2-phenyl-4-trimethylsilylbutan-2-ol | *Winnie* | Mouse | Feces | >8-24 | GC-MS | [8] |
| 3-Oxalomalate | *Winnie* | Mouse | Feces | >8-24 | GC-MS | [8] |
| 4-(2-Aminophenyl)-2,4-dioxobutanoic acid | TNBS | Rat | Urine | ? | UPLC-ESI-qTOF-MS | [18] |
| 4,6-Dihydroxyquinoline | TNBS | Rat | Urine | ? | UPLC-ESI-qTOF-MS | [18] |
| 4,8-Dihydroxyquinoline | T-syn deficiency | Mouse | Colon (distal), cecum | >3-8 | UPLC/ToF-MS | [11] |
| 4-Hydroxybenzenesulfonic acid | DSS (C) | Mouse | Spleen | >8-24 | LC-qTOF-MS | [14] |
| 4-Hydroxybenzoic acid | DSS (A) | Mouse | Colon | >8-24 | GC-MS | [13] |
| 4-Hydroxybenzoic acid | DSS (A) | Mouse | Serum | >8-24 | GC-MS | [13] |
| 4-Hydroxybenzoic acid | DSS (A) | Mouse | Colon | >8-24 | GC-MS | [13] |
| 4-Hydroxybutyric acid | DSS (A) | Mouse | Colon | >8-24 | GC-MS | [13] |
| 4-Hydroxyphenylacetic acid | DSS (A) | Mouse | Colon | >8-24 | GC-MS | [13] |
| 4-Hydroxyphenyllactic acid | DSS (A) | Mouse | Colon | >8-24 | GC-MS | [13] |
| 4-Hydroxyphenyllactic acid | *IL10^-/-^* | Mouse | Urine | >3-8 | GC-MS | [16] |
| 4-Hydroxyphenyllactic acid | *IL10^-/-^* | Mouse | Urine | >3-8 | GC-MS | [16] |
| 4-Hydroxyproline | DSS (A) | Mouse | Colon | >8-24 | GC-MS | [13] |
| 5,6-Dihydroxy eicosatrienoic acid | DSS (A) | Rat | Plasma | >8-24 | LC-MS | [2] |
| 5,6-Dihydroxy eicosatrienoic acid | DSS (C) | Rat | Plasma | >8-24 | LC-MS | [2] |
| 5-Aminovaleric acid | *IL10^-/-^* | Mouse | Urine | >3-8 | GC-MS | [16] |
| 5-Aminovaleric acid | *IL10^-/-^* | Mouse | Urine | >8-24 | GC-MS | [16] |
| 5-Aminovaleric acid | *IL10^-/-^* | Mouse | Urine | >8-24 | GC-MS | [16] |
| 5-Aminovaleric acid | *IL10^-/-^* | Mouse | Urine | >3-8 | GC-MS | [19] |
| 5-Aminovaleric acid | *IL10^-/-^* | Mouse | Urine | >8-24 | GC-MS | [19] |
| 5-Aminovaleric acid | *IL10^-/-^* | Mouse | Urine | >8-24 | GC-MS | [19] |
| 5-Aminovaleric acid | *IL10^-/-^* | Mouse | Urine | >8-24 | GC-MS | [19] |
| 5-Aminovaleric acid | *IL10^-/-^* | Mouse | Urine | >8-24 | GC-MS | [19] |
| 5-Hydroxy eicosatetraenoic acid | DSS (C) | Rat | Plasma | >8-24 | LC-MS | [2] |
| 5-Hydroxytryptamine | DSS (A) | Mouse | Plasma | >8-24 | UPLC-MS | [20] |
| 5-Hydroxytryptophan | DSS (A) | Mouse | Plasma | >8-24 | UPLC-MS | [20] |
| 5-Oxoproline | DSS (A) | Mouse | Colon | >8-24 | GC-MS | [13] |
| 5β-Cyprinolsulfate | T-syn deficiency | Mouse | Colon (distal), cecum | 0-3 | UPLC/ToF-MS | [11] |
| 8,9-Dihydroxy eicosatrienoic acid | DSS (A) | Rat | Plasma | >8-24 | LC-MS | [2] |
| 8,9-Dihydroxy eicosatrienoic acid | DSS (C) | Rat | Plasma | >8-24 | LC-MS | [2] |
| 8-Oxogeranial, (6E)- | T-syn deficiency | Mouse | Colon (distal), cecum | >3-8 | UPLC/ToF-MS | [11] |
| 9,12-Octadecadienoic acid (Z,Z)-, trimethylsilyl ester | *Winnie* | Mouse | Feces | >8-24 | GC-MS | [8] |
| 9-Hydroxy octadecadienoic acid | *TNF*^ΔARE/WT^ | Mouse | Ileum (distal) | >8-24 | LC-MS | [3] |
| Acetoacetate | DSS (A) | Mouse | Serum | >3-8 | ^1^H-NMR | [17] |
| Acetylcarnitine | DSS (C) | Mouse | Colon | >8-24 | LC-qTOF-MS | [14] |
| Acetylglutamine, N- | H. hepaticus | Mouse | Serum | >24 | UPLC-ESI-TOF-MS | [12] |
| Acetyl-L-aspartate | T-syn deficiency | Mouse | Colon (distal), cecum | 0-3 | UPLC/ToF-MS | [11] |
| Acylcarnitine C0 | *TNF*^ΔARE/WT^ | Mouse | Ileum (distal) | >8-24 | LC-MS | [3] |
| Acylcarnitine C16-OH | *TNF*^ΔARE/WT^ | Mouse | Ileum (distal) | >8-24 | LC-MS | [3] |
| Acylcarnitine C18 | *TNF*^ΔARE/WT^ | Mouse | Ileum (distal) | >8-24 | LC-MS | [3] |
| Acylcarnitine C2 | *TNF*^ΔARE/WT^ | Mouse | Ileum (distal) | >8-24 | LC-MS | [3] |
| Acylcarnitine C6:1 | *TNF*^ΔARE/WT^ | Mouse | Ileum (distal) | >8-24 | LC-MS | [3] |
| Adenine | DSS (C) | Mouse | Liver | >8-24 | LC-qTOF-MS | [14] |
| Adenosine 5'-monophosphate | DSS (A) | Mouse | Spleen | >8-24 | ^1^H-NMR | [15] |
| Alanine δ 1.48 | *IL10^-/-^* | Mouse | Plasma | >3-8 | ^1^H-NMR | [21] |
| Alanine δ 1.48 | *IL10^-/-^* | Mouse | Plasma | >8-24 | ^1^H-NMR | [21] |
| Alanine, L- | DSS (A) | Mouse | Colon | >8-24 | GC-MS | [13] |
| Aminobutyric acid, a- | DSS (A) | Mouse | Colon | >8-24 | GC-MS | [13] |
| Aminobutyric acid, a- | DSS (A) | Mouse | Colon | >8-24 | GC-MS | [13] |
| Arachidonate | T-syn deficiency | Mouse | Colon (distal), cecum | 0-3 | UPLC/ToF-MS | [11] |
| Arachidonic acid | *TNF*^ΔARE/WT^ | Mouse | Ileum (distal) | >8-24 | LC-MS | [3] |
| Arginine δ 1.68 | *IL10^-/-^* | Mouse | Plasma | >3-8 | ^1^H-NMR | [21] |
| Arginine δ 1.68 | *IL10^-/-^* | Mouse | Plasma | >8-24 | ^1^H-NMR | [21] |
| Arginine, L- | DSS (C) | Mouse | Liver | >8-24 | LC-qTOF-MS | [14] |
| Asparagine | DSS (A) | Mouse | Colon | >8-24 | GC-MS | [13] |
| Asparagine, L- | DSS (C) | Mouse | Liver | >8-24 | LC-qTOF-MS | [14] |
| Asparaginyl-Histidine/HistidinylAsparagine | DSS (C) | Mouse | Liver | >8-24 | LC-qTOF-MS | [14] |
| Benzenepropanoic acid, .α.-[(trimethylsilyl)oxy]-, trimethylsilyl ester | *Winnie* | Mouse | Feces | >8-24 | GC-MS | [8] |
| Benzenepropanoic acid, 3-[(trimethylsilyl)oxy]-, trimethylsilyl ester | *Winnie* | Mouse | Feces | >8-24 | GC-MS | [8] |
| Butanal, 2,3,4-tris[(trimethylsilyl)oxy]-3-[[(trimethylsilyl)oxy]methyl]-, O-methyloxime, (S)- | *Winnie* | Mouse | Feces | >8-24 | GC-MS | [8] |
| Butane, 2,3-bis(trimethylsiloxy)- | *Winnie* | Mouse | Feces | >8-24 | GC-MS | [8] |
| Butanoic acid | DSS (A) | Mouse | Colon | >8-24 | GC-MS | [22] |
| Butyl ester of (3-Trimethylsilyloxy-2-cyclopentenyl)-thioacetic acid, S-t- | *Winnie* | Mouse | Feces | >8-24 | GC-MS | [8] |
| Butyrate | DSS (A) | Mouse | Urine | >8-24 | ^1^H-NMR | [15] |
| Butyrate | DSS (A) | Mouse | Urine | >8-24 | ^1^H-NMR | [15] |
| Butyrate | DSS (A) | Mouse | Urine | >8-24 | ^1^H-NMR | [15] |
| Butyrate | DSS (A) | Mouse | Urine | >8-24 | ^1^H-NMR | [15] |
| Carnitine | DSS (C) | Mouse | Colon | >8-24 | LC-qTOF-MS | [14] |
| Ceramide 16 | DSS (A) | Mouse | Serum | >3-24 | UPLC-ESI-qTOFMS | [6] |
| Ceramide 20 | DSS (A) | Mouse | Serum | >3-24 | UPLC-ESI-qTOFMS | [6] |
| Cholesterol (Total) | Adoptive transfer | Mouse | Liver | >8-24 | ^1^H-NMR | [1] |
| Cholesterol (Total) | *TNF*^ΔARE/WT^ | Mouse | Ileum (distal) | >8-24 | ^1^H-NMR | [3] |
| Cholesterol (Total) | *TNF*^ΔARE/WT^ | Mouse | Colon (proximal) | >8-24 | ^1^H-NMR | [3] |
| Cholic acid | TNBS | Rat | Plasma | ? | UPLC-ESI-qTOF-MS | [18] |
| Choline in phospholipids | *TNF*^ΔARE/WT^ | Mouse | Ileum (distal) | >8-24 | ^1^H-NMR | [3] |
| Choline in phospholipids | *TNF*^ΔARE/WT^ | Mouse | Colon (proximal) | >8-24 | ^1^H-NMR | [3] |
| Choline in phospholipids δ 3.20-3.23 | *IL10^-/-^* | Mouse | Plasma | >3-8 | ^1^H-NMR | [21] |
| Choline in phospholipids δ 3.20-3.23 | *IL10^-/-^* | Mouse | Plasma | >8-24 | ^1^H-NMR | [21] |
| Citraconic acid | DSS (A) | Mouse | Colon | >8-24 | GC-MS | [13] |
| Citraconic acid | DSS (A) | Mouse | Colon | >8-24 | GC-MS | [13] |
| Citrate | DSS (A) | Mouse | Urine | >8-24 | ^1^H-NMR | [15] |
| Citrate | DSS (A) | Mouse | Urine | >8-24 | ^1^H-NMR | [15] |
| Citrate | DSS (A) | Mouse | Urine | >8-24 | ^1^H-NMR | [15] |
| Citrate | DSS (A) | Mouse | Urine | >8-24 | ^1^H-NMR | [15] |
| Citrate | TNBS | Rat | Urine | ? | UPLC-MS/MS | [23] |
| Citrate | TNBS | Rat | Urine | ? | UPLC-MS/MS | [23] |
| Citrate | TNBS | Rat | Serum | ? | UPLC-MS/MS | [23] |
| Citrate δ 2.55 | *IL10^-/-^* | Mouse | Plasma | 0-3 | ^1^H-NMR | [21] |
| Citrate δ 2.55 | *IL10^-/-^* | Mouse | Plasma | >8-24 | ^1^H-NMR | [21] |
| Citrulline | Adoptive transfer | Mouse | Stool | >8-24 | ^1^H-NMR | [1] |
| Compound_1 | *Winnie* | Mouse | Feces | >8-24 | GC-MS | [8] |
| Compound_105 | *Winnie* | Mouse | Feces | >8-24 | GC-MS | [8] |
| Compound_106 | *Winnie* | Mouse | Feces | >8-24 | GC-MS | [8] |
| Cortol | T-syn deficiency | Mouse | Colon (distal), cecum | >8-24 | UPLC/ToF-MS | [11] |
| Creatine | DSS (A) | Mouse | Serum | >3-8 | ^1^H-NMR | [17] |
| Creatine δ 3.03 | *IL10^-/-^* | Mouse | Plasma | >3-8 | ^1^H-NMR | [21] |
| Creatinine | *IL10^-/-^* | Mouse | Urine | >8-24 | NMR | [4] |
| Cresol glucuronide, p- | DSS (C) | Mouse | Colon | >8-24 | LC-qTOF-MS | [14] |
| Cresol glucuronide, p- | TNBS | Rat | Urine | ? | UPLC-ESI-qTOF-MS | [18] |
| Cresol sulfate, p- | DSS (C) | Mouse | Colon | >8-24 | LC-qTOF-MS | [14] |
| Cytidine 5'-monophosphate | DSS (A) | Mouse | Liver | >8-24 | ^1^H-NMR | [15] |
| Cytosine | *IL10^-/-^* | Mouse | Urine | >3-8 | GC-MS | [16] |
| Cytosine | *IL10^-/-^* | Mouse | Urine | >3-8 | GC-MS | [16] |
| Cytosine | *IL10^-/-^* | Mouse | Urine | >8-24 | GC-MS | [16] |
| Cytosine | *IL10^-/-^* | Mouse | Urine | >8-24 | GC-MS | [16] |
| Cytosine | *IL10^-/-^* | Mouse | Urine | >3-8 | GC-MS | [19] |
| Cytosine | *IL10^-/-^* | Mouse | Urine | >8-24 | GC-MS | [19] |
| Cytosine | *IL10^-/-^* | Mouse | Urine | >8-24 | GC-MS | [19] |
| Daidzein | H. hepaticus | Mouse | Serum | >8-24 | UPLC-ESI-TOF-MS | [12] |
| Daidzein | H. hepaticus | Mouse | Serum | >24 | UPLC-ESI-TOF-MS | [12] |
| Decanoic acid | DSS (A) | Mouse | Serum | >8-24 | GC-MS | [13] |
| Dehydroascorbic acid | DSS (C) | Mouse | Colon | >8-24 | LC-qTOF-MS | [14] |
| Dextran | DSS (A) | Mouse | Feces | >3-8 | ^1^H-NMR | [24] |
| Diacylglycerol | *TNF*^ΔARE/WT^ | Mouse | Colon (proximal) | >8-24 | ^1^H-NMR | [3] |
| Dihydroxy octadecenoic acids (incl. 9,10-Dihydroxy octadecenoic acid) | *IL10^-/-^* | Mouse | Plasma | >24 | LC/MS-MS | [9] |
| Dihydroxy octadecenoic acids (incl. 9,10-Dihydroxy octadecenoic acid) | *IL10^-/-^* | Mouse | Plasma | >24 | LC/MS-MS | [9] |
| Dihydroxyeicosatrienoic acids (incl. 11,12-Dihydroxyeicosatrienoic acid) | *IL10^-/-^* | Mouse | Plasma | >24 | LC/MS-MS | [9] |
| Dihydroxyeicosatrienoic acids (incl. 11,12-Dihydroxyeicosatrienoic acid) | *IL10^-/-^* | Mouse | Plasma | >24 | LC/MS-MS | [9] |
| Dimethylamine | TNBS | Rat | Urine | ? | UPLC-MS/MS | [23] |
| Dimethylamine | TNBS | Rat | Urine | ? | UPLC-MS/MS | [23] |
| Dimethylglycine δ 2.93 | *IL10^-/-^* | Mouse | Plasma | >3-8 | ^1^H-NMR | [21] |
| Ester cholesterol | *TNF*^ΔARE/WT^ | Mouse | Ileum (distal) | >8-24 | ^1^H-NMR | [3] |
| Ester cholesterol | *TNF*^ΔARE/WT^ | Mouse | Colon (proximal) | >8-24 | ^1^H-NMR | [3] |
| Ethanimidic acid, N-(trimethylsilyl)-, trimethylsilyl ester | *Winnie* | Mouse | Feces | >8-24 | GC-MS | [8] |
| Ethylmalonic acid | DSS (A) | Mouse | Colon | >8-24 | GC-MS | [13] |
| Ethylmalonic acid | DSS (A) | Mouse | Colon | >8-24 | GC-MS | [13] |
| Ferulic acid, cis- (2TMS) | *Winnie* | Mouse | Feces | >8-24 | GC-MS | [8] |
| Flavin adenine dinucleotide | H. hepaticus | Mouse | Serum | >24 | UPLC-ESI-TOF-MS | [12] |
| Free cholesterol | Adoptive transfer | Mouse | Liver | >8-24 | ^1^H-NMR | [1] |
| Free cholesterol | *TNF*^ΔARE/WT^ | Mouse | Ileum (distal) | >8-24 | ^1^H-NMR | [3] |
| Free cholesterol | *TNF*^ΔARE/WT^ | Mouse | Colon (proximal) | >8-24 | ^1^H-NMR | [3] |
| Fructose, D- (1MEOX) (5TMS) | *Winnie* | Mouse | Feces | >8-24 | GC-MS | [8] |
| Fucose | *IL10^-/-^* | Mouse | Urine | >3-8 | GC-MS | [16] |
| Fucose | *IL10^-/-^* | Mouse | Urine | >3-8 | GC-MS | [16] |
| Fucose | *IL10^-/-^* | Mouse | Urine | >8-24 | GC-MS | [16] |
| Fucose | *IL10^-/-^* | Mouse | Urine | >8-24 | GC-MS | [16] |
| Fucose | *IL10^-/-^* | Mouse | Urine | >8-24 | GC-MS | [16] |
| Fucose | *IL10^-/-^* | Mouse | Urine | >8-24 | GC-MS | [16] |
| Fucose | *IL10^-/-^* | Mouse | Urine | >3-8 | GC-MS | [19] |
| Fucose | *IL10^-/-^* | Mouse | Urine | >3-8 | GC-MS | [19] |
| Fucose | *IL10^-/-^* | Mouse | Urine | >3-8 | GC-MS | [19] |
| Fucose | *IL10^-/-^* | Mouse | Urine | >8-24 | GC-MS | [19] |
| Fucose | *IL10^-/-^* | Mouse | Urine | >8-24 | GC-MS | [19] |
| Fucose | *IL10^-/-^* | Mouse | Urine | >8-24 | GC-MS | [19] |
| Fucose | *IL10^-/-^* | Mouse | Urine | >8-24 | GC-MS | [19] |
| Fucose | *IL10^-/-^* | Mouse | Urine | >8-24 | NMR | [4] |
| Fucose | *IL10^-/-^* | Mouse | Urine | >8-24 | NMR | [4] |
| Fucose | *IL10^-/-^* | Mouse | Urine | >8-24 | NMR | [4] |
| Fucose, DL- (1MEOX) (4TMS) | *Winnie* | Mouse | Feces | >8-24 | GC-MS | [8] |
| Fumarate | DSS (A) | Mouse | Urine | >8-24 | ^1^H-NMR | [15] |
| Fumarate | DSS (A) | Mouse | Urine | >8-24 | ^1^H-NMR | [15] |
| Fumarate | DSS (A) | Mouse | Urine | >8-24 | ^1^H-NMR | [15] |
| Fumarate | DSS (A) | Mouse | Urine | >8-24 | ^1^H-NMR | [15] |
| Fumarate | DSS (A) | Mouse | Urine | >8-24 | ^1^H-NMR | [15] |
| Fumarate δ 6.51 | *IL10^-/-^* | Mouse | Plasma | >3-8 | ^1^H-NMR | [21] |
| Glucose | *IL10^-/-^* | Mouse | Urine | >8-24 | GC-MS | [19] |
| Glucose, D-(+)- | *Winnie* | Mouse | Feces | >8-24 | GC-MS | [8] |
| Glucose-1-phosphate, dipotassium salt dihydrate, α-D- | *Winnie* | Mouse | Feces | >8-24 | GC-MS | [8] |
| Glutamic acid, L- | DSS (A) | Mouse | Colon | >8-24 | GC-MS | [13] |
| Glutamine δ 2.45 | *IL10^-/-^* | Mouse | Plasma | 0-3 | ^1^H-NMR | [21] |
| Glutamine δ 2.45 | *IL10^-/-^* | Mouse | Plasma | >3-8 | ^1^H-NMR | [21] |
| Glutamine, L- | DSS (C) | Mouse | Colon | >8-24 | LC-qTOF-MS | [14] |
| Glutamine, L- | DSS (C) | Mouse | Colon | >8-24 | LC-qTOF-MS | [14] |
| Glycero-3-phosphocholine, sn- | T-syn deficiency | Mouse | Colon (distal), cecum | >3-8 | UPLC/ToF-MS | [11] |
| Glycerol | DSS (A) | Mouse | Plasma | >8-24 | ^1^H-NMR | [15] |
| Glycerol (3TMS) | *Winnie* | Mouse | Feces | >8-24 | GC-MS | [8] |
| Glycerol (3TMS) | *Winnie* | Mouse | Feces | >8-24 | GC-MS | [8] |
| Glycerophosphocholine δ 4.35 | *IL10^-/-^* | Mouse | Plasma | >3-8 | ^1^H-NMR | [21] |
| Glycerophospholipids | Adoptive transfer | Mouse | Liver | >8-24 | ^1^H-NMR | [1] |
| Glycerophospholipids | *TNF*^ΔARE/WT^ | Mouse | Ileum (distal) | >8-24 | ^1^H-NMR | [3] |
| Glycerophospholipids | *TNF*^ΔARE/WT^ | Mouse | Colon (proximal) | >8-24 | ^1^H-NMR | [3] |
| Glycine | Adoptive transfer | Mouse | Stool | >8-24 | ^1^H-NMR | [1] |
| Glycine | DSS (A) | Mouse | Colon | >8-24 | GC-MS | [22] |
| Glycine, N-(2-methyl-1-oxobutyl)-, trimethylsilyl ester | *Winnie* | Mouse | Feces | >8-24 | GC-MS | [8] |
| Glycocholic acid | DSS (C) | Mouse | Spleen | >8-24 | LC-qTOF-MS | [14] |
| Glycolate | DSS (A) | Mouse | Serum | >3-8 | ^1^H-NMR | [17] |
| Glycolic acid | DSS (A) | Mouse | Colon | >8-24 | GC-MS | [13] |
| Glycolic acid | DSS (A) | Mouse | Colon | >8-24 | GC-MS | [13] |
| Glycoproteins δ 4.16 | *IL10^-/-^* | Mouse | Plasma | >3-8 | ^1^H-NMR | [21] |
| Glycoproteins δ 4.16 | *IL10^-/-^* | Mouse | Plasma | >8-24 | ^1^H-NMR | [21] |
| Glyoxylic acid | DSS (A) | Mouse | Serum | >8-24 | GC-MS | [13] |
| Glyoxylic acid | DSS (A) | Mouse | Colon | >8-24 | GC-MS | [13] |
| Glyoxylic acid | DSS (A) | Mouse | Serum | >8-24 | GC-MS | [13] |
| Glyoxylic acid | DSS (A) | Mouse | Colon | >8-24 | GC-MS | [13] |
| GPL PCaa C32:2 | *TNF*^ΔARE/WT^ | Mouse | Ileum (distal) | >3-8 | LC-MS | [3] |
| GPL PCaa C36:6 | *TNF*^ΔARE/WT^ | Mouse | Ileum (distal) | >8-24 | LC-MS | [3] |
| GPL PCae C38:3 | *TNF*^ΔARE/WT^ | Mouse | Ileum (distal) | >8-24 | LC-MS | [3] |
| GPL PCae C38:4 | *TNF*^ΔARE/WT^ | Mouse | Ileum (distal) | >8-24 | LC-MS | [3] |
| GPL PCae C38:5 | *TNF*^ΔARE/WT^ | Mouse | Ileum (distal) | >8-24 | LC-MS | [3] |
| GPL PCae C38:6 | *TNF*^ΔARE/WT^ | Mouse | Ileum (distal) | >8-24 | LC-MS | [3] |
| GPL PCae C40:3 | *TNF*^ΔARE/WT^ | Mouse | Ileum (distal) | >8-24 | LC-MS | [3] |
| GPL PCae C42:3 | *TNF*^ΔARE/WT^ | Mouse | Ileum (distal) | >8-24 | LC-MS | [3] |
| Heptadecanoic acid tert-butyl-dimethylsilyl ester | *Winnie* | Mouse | Feces | >8-24 | GC-MS | [8] |
| Heptadecanoic acid, trimethylsilyl ester | *Winnie* | Mouse | Feces | >8-24 | GC-MS | [8] |
| Hexadecanoic acid, n- (1TMS) | *Winnie* | Mouse | Feces | >8-24 | GC-MS | [8] |
| Hexanoylglycine | DSS (A) | Mouse | Colon | >8-24 | GC-MS | [13] |
| Hippurate | TNBS | Rat | Urine | ? | UPLC-MS/MS | [23] |
| Hippurate | TNBS | Rat | Feces | ? | UPLC-MS/MS | [23] |
| Hippurate | TNBS | Rat | Feces | ? | UPLC-MS/MS | [23] |
| Hippurate | TNBS | Rat | Serum | ? | UPLC-MS/MS | [23] |
| Hippuric acid | *IL10^-/-^* | Mouse | Urine | >3-8 | GC-MS | [19] |
| Hippuric acid | *IL10^-/-^* | Mouse | Urine | >8-24 | GC-MS | [19] |
| Hippuric acid | *IL10^-/-^* | Mouse | Urine | >8-24 | GC-MS | [19] |
| Hippuric acid | *IL10^-/-^* | Mouse | Urine | >8-24 | GC-MS | [19] |
| Histidine, L- | DSS (C) | Mouse | Liver | >8-24 | LC-qTOF-MS | [14] |
| Hydroxyphenylbutazone glucuronide, γ- | DSS (C) | Mouse | Colon | >8-24 | LC-qTOF-MS | [14] |
| Hypoxanthine | DSS (A) | Mouse | Serum | >3-8 | ^1^H-NMR | [17] |
| Indole-1-acetic acid, 1H- | *Winnie* | Mouse | Feces | >8-24 | GC-MS | [8] |
| Inosine | DSS (A) | Mouse | Serum | >3-8 | ^1^H-NMR | [17] |
| Inositol, 1,2,3,4,5,6-hexakis-O-(trimethylsilyl)-, D-chiro- | *Winnie* | Mouse | Feces | >8-24 | GC-MS | [8] |
| Inositol, 1,2,3,4,5,6-hexakis-O-(trimethylsilyl)-, Neo- | *Winnie* | Mouse | Feces | >8-24 | GC-MS | [8] |
| Inulobiose (impurity: Sucrose) | *Winnie* | Mouse | Feces | >8-24 | GC-MS | [8] |
| Isobutyryl carnitine | DSS (C) | Mouse | Colon | >8-24 | LC-qTOF-MS | [14] |
| Isoleucine | Adoptive transfer | Mouse | Stool | >8-24 | ^1^H-NMR | [1] |
| Isoleucine δ 0.92 | *IL10^-/-^* | Mouse | Plasma | >8-24 | ^1^H-NMR | [21] |
| Isoleucine δ 0.92 | *IL10^-/-^* | Mouse | Plasma | >8-24 | ^1^H-NMR | [21] |
| Isoleucine, L- | DSS (A) | Mouse | Colon | >8-24 | GC-MS | [13] |
| Isoleucine, L- | DSS (A) | Mouse | Serum | >8-24 | GC-MS | [13] |
| Isoleucine, L- | DSS (A) | Mouse | Colon | >8-24 | GC-MS | [13] |
| Isovaleroylglycine, N- | DSS (A) | Mouse | Serum | >3-8 | ^1^H-NMR | [17] |
| Isovalerylglycine | DSS (A) | Mouse | Serum | >8-24 | GC-MS | [13] |
| Kynurenine | *IL10^-/-^* | Mouse | Plasma | >8-24 | LC-MS | [16] |
| Kynurenine | *IL10^-/-^* | Mouse | Plasma | >8-24 | LC-MS | [16] |
| Kynurenine | *IL10^-/-^* | Mouse | Plasma | >8-24 | LC-MS | [16] |
| Kynurenine, L- | DSS (A) | Mouse | Plasma | >8-24 | UPLC-MS | [20] |
| Kynurenine, L- | DSS (A) | Mouse | Plasma | >8-24 | UPLC-MS | [20] |
| Lactate | DSS (A) | Mouse | Colon | >8-24 | NMR(1H, 1C, 1P) | [25] |
| Lactate δ 4.11 | *IL10^-/-^* | Mouse | Plasma | >3-8 | ^1^H-NMR | [21] |
| Lactate δ 4.11 | *IL10^-/-^* | Mouse | Plasma | >8-24 | ^1^H-NMR | [21] |
| Leucine, L- | DSS (A) | Mouse | Serum | >8-24 | GC-MS | [13] |
| Leucine, L- | DSS (A) | Mouse | Colon | >8-24 | GC-MS | [13] |
| Leucine, L- | DSS (A) | Mouse | Serum | >8-24 | GC-MS | [13] |
| Leucine, L- | DSS (A) | Mouse | Colon | >8-24 | GC-MS | [13] |
| LeukotrieneF4 | T-syn deficiency | Mouse | Colon (distal), cecum | 0-3 | UPLC/ToF-MS | [11] |
| Linoleate | T-syn deficiency | Mouse | Colon (distal), cecum | >3-8 | UPLC/ToF-MS | [11] |
| Lipid | DSS (A) | Mouse | Plasma | >8-24 | ^1^H-NMR | [15] |
| Lipoproteins mainly HDL/LDL δ 0.81-0.88 | *IL10^-/-^* | Mouse | Plasma | 0-3 | ^1^H-NMR | [21] |
| Lipoproteins mainly HDL/LDL δ 0.81-0.88 | *IL10^-/-^* | Mouse | Plasma | >8-24 | ^1^H-NMR | [21] |
| Lipoproteins δ 1.18-1.25 | *IL10^-/-^* | Mouse | Plasma | 0-3 | ^1^H-NMR | [21] |
| Lipoproteins δ 1.18-1.25 | *IL10^-/-^* | Mouse | Plasma | >8-24 | ^1^H-NMR | [21] |
| Lithocholic acid | *Winnie* | Mouse | Feces | >8-24 | GC-MS | [8] |
| Lotaustralin | T-syn deficiency | Mouse | Colon (distal), cecum | 0-3 | UPLC/ToF-MS | [11] |
| Lysine | Adoptive transfer | Mouse | Stool | >8-24 | ^1^H-NMR | [1] |
| Lysine | DSS (A) | Mouse | Plasma | >8-24 | ^1^H-NMR | [15] |
| Lysine | DSS (A) | Mouse | Colon | >8-24 | ^1^H-NMR | [15] |
| Lysine | DSS (A) | Mouse | Liver | >8-24 | ^1^H-NMR | [15] |
| Lysine δ 1.72 | *IL10^-/-^* | Mouse | Plasma | >3-8 | ^1^H-NMR | [21] |
| LysoPC (16:0) | TNBS | Rat | Plasma | ? | UPLC-ESI-qTOF-MS | [18] |
| LysoPC (17:0) | DSS (C) | Mouse | Liver | >8-24 | LC-qTOF-MS | [14] |
| LysoPC (18:0) | TNBS | Rat | Plasma | ? | UPLC-ESI-qTOF-MS | [18] |
| LysoPC (18:1) [two indistinguishable isomers - 11Z and 9Z] | TNBS | Rat | Plasma | ? | UPLC-ESI-qTOF-MS | [18] |
| LysoPC (20 : 3(5Z,8Z,11Z)) | DSS (C) | Mouse | Colon | >8-24 | LC-qTOF-MS | [14] |
| LysoPC (20 : 3) | DSS (C) | Mouse | Colon | >8-24 | LC-qTOF-MS | [14] |
| LysoPC (20:4) | TNBS | Rat | Plasma | ? | UPLC-ESI-qTOF-MS | [18] |
| Maleic acid | DSS (A) | Mouse | Colon | >8-24 | GC-MS | [13] |
| Maleic acid | DSS (A) | Mouse | Colon | >8-24 | GC-MS | [13] |
| Malic acid | DSS (A) | Mouse | Serum | >8-24 | GC-MS | [22] |
| Malic acid | DSS (A) | Mouse | Colon | >8-24 | GC-MS | [13] |
| Mannopine | T-syn deficiency | Mouse | Colon (distal), cecum | 0-3 | UPLC/ToF-MS | [11] |
| Mannose | DSS (A) | Mouse | Serum | >3-8 | ^1^H-NMR | [17] |
| Methionine δ 2.14 | *IL10^-/-^* | Mouse | Plasma | >3-8 | ^1^H-NMR | [21] |
| Methionine, DL- (2TMS) | *Winnie* | Mouse | Feces | >8-24 | GC-MS | [8] |
| Methionine, L- | DSS (A) | Mouse | Colon | >8-24 | GC-MS | [13] |
| Methyl phosphate | DSS (A) | Mouse | Colon | >8-24 | ^1^H-NMR | [15] |
| Monosaccharide | DSS (A) | Mouse | Feces | >3-8 | ^1^H-NMR | [24] |
| Mucic acid | *Winnie* | Mouse | Feces | >8-24 | GC-MS | [8] |
| Myristoylcarnitine, (±)- | DSS (C) | Mouse | Colon | >8-24 | LC-qTOF-MS | [14] |
| Octadecanoic acid, n- (1TMS) | *Winnie* | Mouse | Feces | >8-24 | GC-MS | [8] |
| Octanoic acid | DSS (A) | Mouse | Serum | >8-24 | GC-MS | [13] |
| Octanoic acid | DSS (A) | Mouse | Serum | >8-24 | GC-MS | [13] |
| Octanoic acid | T-syn deficiency | Mouse | Colon (distal), cecum | >3-8 | UPLC/ToF-MS | [11] |
| Oleic acid, trimethylsilyl ester | *Winnie* | Mouse | Feces | >8-24 | GC-MS | [8] |
| Ornithine | Adoptive transfer | Mouse | Stool | >8-24 | ^1^H-NMR | [1] |
| Oxalic acid | DSS (A) | Mouse | Colon | >8-24 | GC-MS | [13] |
| Oxalic acid | DSS (A) | Mouse | Colon | >8-24 | GC-MS | [13] |
| Oxalic acid dihydrate | *Winnie* | Mouse | Feces | >8-24 | GC-MS | [8] |
| Oxidized glutathione | DSS (A) | Mouse | Spleen | >8-24 | ^1^H-NMR | [15] |
| Palmitoyl histidine, N- | DSS (C) | Mouse | Colon | >8-24 | LC-qTOF-MS | [14] |
| Palmitoyl-L-carnitine | DSS (C) | Mouse | Colon | >8-24 | LC-qTOF-MS | [14] |
| PC (10 : 2(2E,4E)/10 : 2(2E,4E))[S] | DSS (C) | Mouse | Colon | >8-24 | LC-qTOF-MS | [14] |
| PE (22:1(13Z)/14:0) | DSS (C) | Mouse | Liver | >8-24 | LC-qTOF-MS | [14] |
| PG (16:0/22:4(7Z,10Z,13Z,16Z)) | DSS (C) | Mouse | Liver | >8-24 | LC-qTOF-MS | [14] |
| PG (18:3(6Z,9Z,12Z)/18:3(6Z,9Z,12Z)) | DSS (C) | Mouse | Liver | >8-24 | LC-qTOF-MS | [14] |
| PG (18:3(9Z,12Z,15Z)/13:0) | DSS (C) | Mouse | Spleen | >8-24 | LC-qTOF-MS | [14] |
| Phenylacetate | DSS (A) | Mouse | Urine | >8-24 | ^1^H-NMR | [15] |
| Phenylacetate | DSS (A) | Mouse | Urine | >8-24 | ^1^H-NMR | [15] |
| Phenylacetate | DSS (A) | Mouse | Urine | >8-24 | ^1^H-NMR | [15] |
| Phenylacetate | DSS (A) | Mouse | Urine | >8-24 | ^1^H-NMR | [15] |
| Phenylacetate | DSS (A) | Mouse | Urine | >8-24 | ^1^H-NMR | [15] |
| Phenylacetylglycine | *IL10^-/-^* | Mouse | Urine | >8-24 | NMR | [4] |
| Phenylacetylglycine | *IL10^-/-^* | Mouse | Urine | >8-24 | NMR | [4] |
| Phenylacetylglycine | TNBS | Rat | Urine | ? | UPLC-ESI-qTOF-MS | [18] |
| Phenylacetylglycine | H. hepaticus | Mouse | Serum | >24 | UPLC-ESI-TOF-MS | [12] |
| Phenylacetylglycine | TNBS | Rat | Urine | ? | UPLC-MS/MS | [23] |
| Phenylacetylglycine | TNBS | Rat | Urine | ? | UPLC-MS/MS | [23] |
| Phenylacetylglycine | TNBS | Rat | Urine | ? | UPLC-MS/MS | [23] |
| Phenylalanine | Adoptive transfer | Mouse | Stool | >8-24 | ^1^H-NMR | [1] |
| Phenylalanine | DSS (A) | Mouse | Plasma | >8-24 | ^1^H-NMR | [15] |
| Phenylalanine (2TMS)/N,O-Bis(trimethylsilyl)-L-phenylalanine | *Winnie* | Mouse | Feces | >8-24 | GC-MS | [8] |
| Phenylalanine δ 7.44 | *IL10^-/-^* | Mouse | Plasma | >3-8 | ^1^H-NMR | [21] |
| Phenylalanine δ 7.44 | *IL10^-/-^* | Mouse | Plasma | >8-24 | ^1^H-NMR | [21] |
| Phenylalanine δ 7.44 | *IL10^-/-^* | Mouse | Plasma | >8-24 | ^1^H-NMR | [21] |
| Phenylalanine, L- | DSS (A) | Mouse | Serum | >8-24 | GC-MS | [13] |
| Phenylalanine, L- | DSS (A) | Mouse | Colon | >8-24 | GC-MS | [13] |
| Phenylalanine, L- | DSS (A) | Mouse | Serum | >8-24 | GC-MS | [13] |
| Phenylalanine, L- | DSS (A) | Mouse | Colon | >8-24 | GC-MS | [13] |
| Phenyllactic acid, L-(-)-3- | *Winnie* | Mouse | Feces | >8-24 | GC-MS | [8] |
| Phosphatidic acid (P-20:0/22:2(13Z,16Z)) | DSS (C) | Mouse | Spleen | >8-24 | LC-qTOF-MS | [14] |
| Phosphocholine | Adoptive transfer | Mouse | Liver | >8-24 | ^1^H-NMR | [1] |
| Phosphocholine | DSS (A) | Mouse | Liver | >8-24 | ^1^H-NMR | [15] |
| Phosphoinositol (13:0/18:3(6Z,9Z,12Z)) | DSS (C) | Mouse | Spleen | >8-24 | LC-qTOF-MS | [14] |
| Phosphoric acid (3TMS) | *Winnie* | Mouse | Feces | >8-24 | GC-MS | [8] |
| Phytol mixture of isomers | *Winnie* | Mouse | Feces | >8-24 | GC-MS | [8] |
| Plasmalogen | Adoptive transfer | Mouse | Liver | >8-24 | ^1^H-NMR | [1] |
| Plasmalogen | *TNF*^ΔARE/WT^ | Mouse | Ileum (distal) | >8-24 | ^1^H-NMR | [3] |
| Plasmalogen | *TNF*^ΔARE/WT^ | Mouse | Colon (proximal) | >8-24 | ^1^H-NMR | [3] |
| Platelet activating factor C-16, Enantio- | DSS (C) | Mouse | Colon | >8-24 | LC-qTOF-MS | [14] |
| Polyunsaturated fatty acids | Adoptive transfer | Mouse | Liver | >8-24 | ^1^H-NMR | [1] |
| Polyunsaturated fatty acids (δ=2.79-2.87) | *TNF*^ΔARE/WT^ | Mouse | Ileum (distal) | >8-24 | ^1^H-NMR | [3] |
| Polyunsaturated fatty acids (δ=2.79-2.87) | *TNF*^ΔARE/WT^ | Mouse | Colon (proximal) | >8-24 | ^1^H-NMR | [3] |
| Polyunsaturated fatty acids δ 2.73 | *IL10^-/-^* | Mouse | Plasma | 0-3 | ^1^H-NMR | [21] |
| Polyunsaturated fatty acids δ 2.73 | *IL10^-/-^* | Mouse | Plasma | >3-8 | ^1^H-NMR | [21] |
| Polyunsaturated fatty acids δ 2.73 | *IL10^-/-^* | Mouse | Plasma | >8-24 | ^1^H-NMR | [21] |
| Polyunsaturated fatty acids δ 2.76 | *IL10^-/-^* | Mouse | Plasma | 0-3 | ^1^H-NMR | [21] |
| Polyunsaturated fatty acids δ 2.76 | *IL10^-/-^* | Mouse | Plasma | >8-24 | ^1^H-NMR | [21] |
| Polyunsaturated fatty acids δ 2.78 | *IL10^-/-^* | Mouse | Plasma | >8-24 | ^1^H-NMR | [21] |
| Polyunsaturated fatty acids δ 2.78 | *IL10^-/-^* | Mouse | Plasma | >8-24 | ^1^H-NMR | [21] |
| Proline, L- | DSS (A) | Mouse | Colon | >8-24 | GC-MS | [13] |
| Proline, L- | DSS (A) | Mouse | Colon | >8-24 | GC-MS | [13] |
| Propionylglycine | DSS (A) | Mouse | Colon | >8-24 | GC-MS | [13] |
| Prostaglandin D2 | DSS (C) | Rat | Colon | >8-24 | LC-MS | [2] |
| Prostaglandin D3 | DSS (C) | Rat | Colon | >8-24 | LC-MS | [2] |
| Prostaglandin E1 | DSS (C) | Rat | Colon | >8-24 | LC-MS | [2] |
| Prostaglandin E2 | DSS (C) | Rat | Colon | >8-24 | LC-MS | [2] |
| Prostaglandin E3 | DSS (C) | Rat | Colon | >8-24 | LC-MS | [2] |
| Prostaglandin F1α | *TNF*^ΔARE/WT^ | Mouse | Ileum (distal) | >8-24 | LC-MS | [3] |
| Prostaglandin F2α | *TNF*^ΔARE/WT^ | Mouse | Ileum (distal) | >8-24 | LC-MS | [3] |
| Prostaglandin J2 | DSS (C) | Rat | Colon | >8-24 | LC-MS | [2] |
| PS (15:0/20:5(5Z,8Z,11Z,14Z,17Z)) | DSS (C) | Mouse | Liver | >8-24 | LC-qTOF-MS | [14] |
| PS (20:5(5Z,8Z,11Z,14Z,17Z)/18:1(9Z)) | DSS (C) | Mouse | Spleen | >8-24 | LC-qTOF-MS | [14] |
| PS (21:0/0:0) | DSS (C) | Mouse | Spleen | >8-24 | LC-qTOF-MS | [14] |
| PS(18:1(9Z)/20:5(5Z,8Z,11Z,14Z,17Z)) | DSS (C) | Mouse | Spleen | >8-24 | LC-qTOF-MS | [14] |
| Pyridoxal-5'-phosphate | *IL10^-/-^* | Mouse | Plasma | >8-24 | LC-MS | [16] |
| Pyruvate δ 2.41 | *IL10^-/-^* | Mouse | Plasma | >3-8 | ^1^H-NMR | [21] |
| Pyruvate δ 2.41 | *IL10^-/-^* | Mouse | Plasma | >8-24 | ^1^H-NMR | [21] |
| Pyruvic acid (1MEOX) (1TMS) | *Winnie* | Mouse | Feces | >8-24 | GC-MS | [8] |
| Ribose, D- (1MEOX) (4TMS) | *Winnie* | Mouse | Feces | >8-24 | GC-MS | [8] |
| Ribulose, D- | *Winnie* | Mouse | Feces | >8-24 | GC-MS | [8] |
| Serine, L- | DSS (A) | Mouse | Colon | >8-24 | GC-MS | [13] |
| Shikimic acid-3-phosphate | *Winnie* | Mouse | Feces | >8-24 | GC-MS | [8] |
| Sinapyl alcohol | *Winnie* | Mouse | Feces | >8-24 | GC-MS | [8] |
| Sphingomyelin | Adoptive transfer | Mouse | Liver | >8-24 | ^1^H-NMR | [1] |
| Sphingomyelin | *TNF*^ΔARE/WT^ | Mouse | Ileum (distal) | >8-24 | ^1^H-NMR | [3] |
| Sphingomyelin | *TNF*^ΔARE/WT^ | Mouse | Colon (proximal) | >8-24 | ^1^H-NMR | [3] |
| Sphingomyelin (d18:1/14:0) | DSS (A) | Mouse | Serum | >8-24 | UPLC-MS | [7] |
| Sphingomyelin (d18:1/16:0) | DSS (A) | Mouse | Serum | >8-24 | UPLC-MS | [7] |
| Sphingomyelin (d18:1/18:0) | DSS (A) | Mouse | Serum | >8-24 | UPLC-MS | [7] |
| Sphingomyelin 16:0 | DSS (A) | Mouse | Serum | >3-24 | UPLC-ESI-qTOFMS | [6] |
| Sphingomyelin 18:1 | DSS (A) | Mouse | Serum | >3-24 | UPLC-ESI-qTOFMS | [6] |
| Sphingomyelin 24:1 | DSS (A) | Mouse | Serum | >3-24 | UPLC-ESI-qTOFMS | [6] |
| Sphingomyelin C16:0 | *TNF*^ΔARE/WT^ | Mouse | Ileum (distal) | >8-24 | LC-MS | [3] |
| Sphingomyelin C16:1 | *TNF*^ΔARE/WT^ | Mouse | Ileum (distal) | >3-8 | LC-MS | [3] |
| Sphingomyelin C26:0 | *TNF*^ΔARE/WT^ | Mouse | Ileum (distal) | >3-8 | LC-MS | [3] |
| Succinate | TNBS | Rat | Urine | ? | UPLC-MS/MS | [23] |
| Succinate δ 2.37 | *IL10^-/-^* | Mouse | Plasma | >3-8 | ^1^H-NMR | [21] |
| Succinic acid | DSS (A) | Mouse | Colon | >8-24 | GC-MS | [13] |
| Succinic acid | *IL10^-/-^* | Mouse | Urine | >3-8 | GC-MS | [16] |
| Succinic acid | *IL10^-/-^* | Mouse | Urine | >3-8 | GC-MS | [16] |
| Succinic acid | *IL10^-/-^* | Mouse | Urine | >8-24 | GC-MS | [16] |
| Sugar H1 | *TNF*^ΔARE/WT^ | Mouse | Ileum (distal) | >8-24 | LC-MS | [3] |
| Taurocholate | T-syn deficiency | Mouse | Colon (distal), cecum | >3-8 | UPLC/ToF-MS | [11] |
| Taurodeoxycholic acid | DSS (C) | Mouse | Spleen | >8-24 | LC-qTOF-MS | [14] |
| Thiodiglycolic acid | DSS (A) | Mouse | Colon | >8-24 | GC-MS | [13] |
| Threonine, L- | DSS (A) | Mouse | Serum | >8-24 | GC-MS | [13] |
| Threonine, L- | DSS (A) | Mouse | Colon | >8-24 | GC-MS | [13] |
| Threonine, L- | DSS (A) | Mouse | Serum | >8-24 | GC-MS | [13] |
| Threonine, L- | DSS (A) | Mouse | Colon | >8-24 | GC-MS | [13] |
| Thromboxane B2 | *TNF*^ΔARE/WT^ | Mouse | Ileum (distal) | >3-8 | LC-MS | [3] |
| Thromboxane B2 | *TNF*^ΔARE/WT^ | Mouse | Ileum (distal) | >8-24 | LC-MS | [3] |
| Thromboxane B2 | *TNF*^ΔARE/WT^ | Mouse | Ileum (distal) | >8-24 | LC-MS | [3] |
| Thymine | DSS (A) | Mouse | Serum | >8-24 | GC-MS | [13] |
| Thymine | DSS (A) | Mouse | Colon | >8-24 | GC-MS | [13] |
| Thymine | DSS (A) | Mouse | Colon | >8-24 | GC-MS | [13] |
| Traumatic acid | T-syn deficiency | Mouse | Colon (distal), cecum | >3-8 | UPLC/ToF-MS | [11] |
| Trimethylamine | DSS (A) | Mouse | Feces | >3-8 | ^1^H-NMR | [24] |
| Trimethylamine | *IL10^-/-^* | Mouse | Urine | >8-24 | NMR | [4] |
| Trimethylamine | *IL10^-/-^* | Mouse | Urine | >8-24 | NMR | [4] |
| Trimethylamine | *IL10^-/-^* | Mouse | Urine | >8-24 | NMR | [4] |
| Trimethylamine | TNBS | Rat | Urine | ? | UPLC-MS/MS | [23] |
| Trimethylamine | TNBS | Rat | Urine | ? | UPLC-MS/MS | [23] |
| Trimethylsiloxy-trimethylsilylmethyl-phenylsulfide | *Winnie* | Mouse | Feces | >8-24 | GC-MS | [8] |
| Trimethylsilyl 3-(3,4-bis[(trimethylsilyl)oxy]phenyl)-2-[(trimethylsilyl)oxy]propanoate | *Winnie* | Mouse | Feces | >8-24 | GC-MS | [8] |
| Tryptophan | Adoptive transfer | Mouse | Stool | >8-24 | ^1^H-NMR | [1] |
| Tryptophan, Acetone | DSS (A) | Mouse | Serum | >3-8 | ^1^H-NMR | [17] |
| Tryptophan, L- | DSS (C) | Mouse | Liver | >8-24 | LC-qTOF-MS | [14] |
| Tyramine (3TMS) | *Winnie* | Mouse | Feces | >8-24 | GC-MS | [8] |
| Tyrosine | Adoptive transfer | Mouse | Stool | >8-24 | ^1^H-NMR | [1] |
| Tyrosine δ 6.9 | *IL10^-/-^* | Mouse | Plasma | >3-8 | ^1^H-NMR | [21] |
| Tyrosine, L- | DSS (A) | Mouse | Colon | >8-24 | GC-MS | [13] |
| Unknown at RT 1683 | *IL10^-/-^* | Mouse | Urine | >8-24 | GC-MS | [19] |
| Unknown at RT 1874 | *IL10^-/-^* | Mouse | Urine | >8-24 | GC-MS | [19] |
| Unknown at RT 1874 | *IL10^-/-^* | Mouse | Urine | >8-24 | GC-MS | [19] |
| Unknown at RT1369 | *IL10^-/-^* | Mouse | Urine | >3-8 | GC-MS | [19] |
| Unknown at RT1369 | *IL10^-/-^* | Mouse | Urine | >8-24 | GC-MS | [19] |
| Unknown at RT1369 | *IL10^-/-^* | Mouse | Urine | >8-24 | GC-MS | [19] |
| Unknown at RT1524 | *IL10^-/-^* | Mouse | Urine | >3-8 | GC-MS | [19] |
| Unknown at RT1524 | *IL10^-/-^* | Mouse | Urine | >8-24 | GC-MS | [19] |
| Unknown at RT1524 | *IL10^-/-^* | Mouse | Urine | >8-24 | GC-MS | [19] |
| Unknown at RT1683 | *IL10^-/-^* | Mouse | Urine | >3-8 | GC-MS | [19] |
| Unknown at RT1683 | *IL10^-/-^* | Mouse | Urine | >8-24 | GC-MS | [19] |
| Unknown at RT1683 | *IL10^-/-^* | Mouse | Urine | >8-24 | GC-MS | [19] |
| Unknown at RT1683 | *IL10^-/-^* | Mouse | Urine | >8-24 | GC-MS | [19] |
| Unknown at RT1874 | *IL10^-/-^* | Mouse | Urine | >3-8 | GC-MS | [19] |
| Unknown at RT1874 | *IL10^-/-^* | Mouse | Urine | >3-8 | GC-MS | [19] |
| Unknown at RT1874 | *IL10^-/-^* | Mouse | Urine | >8-24 | GC-MS | [19] |
| Unknown at RT1874 | *IL10^-/-^* | Mouse | Urine | >8-24 | GC-MS | [19] |
| Unknown at RT657 | *IL10^-/-^* | Mouse | Urine | >3-8 | GC-MS | [19] |
| Unknown at RT657 | *IL10^-/-^* | Mouse | Urine | >8-24 | GC-MS | [19] |
| Unknown at RT657 | *IL10^-/-^* | Mouse | Urine | >8-24 | GC-MS | [19] |
| Unknown at RT861 | *IL10^-/-^* | Mouse | Urine | >3-8 | GC-MS | [19] |
| Unknown at RT861 | *IL10^-/-^* | Mouse | Urine | >8-24 | GC-MS | [19] |
| Unknown at RT861 | *IL10^-/-^* | Mouse | Urine | >8-24 | GC-MS | [19] |
| Unknown at RT861 | *IL10^-/-^* | Mouse | Urine | >8-24 | GC-MS | [19] |
| Unknown at RT861 | *IL10^-/-^* | Mouse | Urine | >8-24 | GC-MS | [19] |
| Unknown RT1295n | *IL10^-/-^* | Mouse | Urine | >3-8 | GC-MS | [16] |
| Unknown RT1295n | *IL10^-/-^* | Mouse | Urine | >8-24 | GC-MS | [16] |
| Unknown RT1295n | *IL10^-/-^* | Mouse | Urine | >8-24 | GC-MS | [16] |
| Unknown RT1369 | *IL10^-/-^* | Mouse | Urine | >3-8 | GC-MS | [16] |
| Unknown RT1369 | *IL10^-/-^* | Mouse | Urine | >8-24 | GC-MS | [16] |
| Unknown RT1369 | *IL10^-/-^* | Mouse | Urine | >8-24 | GC-MS | [16] |
| Unknown RT1369 | *IL10^-/-^* | Mouse | Urine | >8-24 | GC-MS | [16] |
| Unknown RT1369 | *IL10^-/-^* | Mouse | Urine | >8-24 | GC-MS | [16] |
| Unknown RT1451n | *IL10^-/-^* | Mouse | Urine | >8-24 | GC-MS | [16] |
| Unknown RT1451n | *IL10^-/-^* | Mouse | Urine | >8-24 | GC-MS | [16] |
| Unknown RT1461n | *IL10^-/-^* | Mouse | Urine | >3-8 | GC-MS | [16] |
| Unknown RT1461n | *IL10^-/-^* | Mouse | Urine | >8-24 | GC-MS | [16] |
| Unknown RT1524 | *IL10^-/-^* | Mouse | Urine | >8-24 | GC-MS | [16] |
| Unknown RT1683 | *IL10^-/-^* | Mouse | Urine | >3-8 | GC-MS | [16] |
| Unknown RT1683 | *IL10^-/-^* | Mouse | Urine | >8-24 | GC-MS | [16] |
| Unknown RT1683 | *IL10^-/-^* | Mouse | Urine | >8-24 | GC-MS | [16] |
| Unknown RT1874 | *IL10^-/-^* | Mouse | Urine | >3-8 | GC-MS | [16] |
| Unknown RT1874 | *IL10^-/-^* | Mouse | Urine | >8-24 | GC-MS | [16] |
| Unknown RT1874 | *IL10^-/-^* | Mouse | Urine | >3-8 | GC-MS | [16] |
| Unknown RT2800 | *IL10^-/-^* | Mouse | Urine | >8-24 | GC-MS | [16] |
| Unknown RT2800 | *IL10^-/-^* | Mouse | Urine | >8-24 | GC-MS | [16] |
| Unknown RT3242n | *IL10^-/-^* | Mouse | Urine | >3-8 | GC-MS | [16] |
| Unknown RT3242n | *IL10^-/-^* | Mouse | Urine | >3-8 | GC-MS | [16] |
| Unknown RT3242n | *IL10^-/-^* | Mouse | Urine | >8-24 | GC-MS | [16] |
| Unknown RT861 | *IL10^-/-^* | Mouse | Urine | >3-8 | GC-MS | [16] |
| Unknown RT861 | *IL10^-/-^* | Mouse | Urine | >3-8 | GC-MS | [16] |
| Unknown RT861 | *IL10^-/-^* | Mouse | Urine | >8-24 | GC-MS | [16] |
| Unknown RT861 | *IL10^-/-^* | Mouse | Urine | >8-24 | GC-MS | [16] |
| Unknown RT954n | *IL10^-/-^* | Mouse | Urine | >3-8 | GC-MS | [16] |
| Unsaturated fatty acids δ 5.25 | *IL10^-/-^* | Mouse | Plasma | >3-8 | ^1^H-NMR | [21] |
| Unsaturated fatty acids δ 5.25 | *IL10^-/-^* | Mouse | Plasma | >8-24 | ^1^H-NMR | [21] |
| Uracil | DSS (A) | Mouse | Serum | >8-24 | GC-MS | [13] |
| Uracil | DSS (A) | Mouse | Colon | >8-24 | GC-MS | [13] |
| Uracil | DSS (A) | Mouse | Serum | >8-24 | GC-MS | [13] |
| Uracil | DSS (A) | Mouse | Colon | >8-24 | GC-MS | [13] |
| Uracil | *IL10^-/-^* | Mouse | Urine | >3-8 | GC-MS | [16] |
| Uracil | *IL10^-/-^* | Mouse | Urine | >8-24 | GC-MS | [16] |
| Uracil | *IL10^-/-^* | Mouse | Urine | >8-24 | GC-MS | [16] |
| Uracil | *IL10^-/-^* | Mouse | Urine | >8-24 | GC-MS | [16] |
| Uracil | *IL10^-/-^* | Mouse | Urine | >8-24 | GC-MS | [19] |
| Uracil | *IL10^-/-^* | Mouse | Urine | >3-8 | NMR | [4] |
| Uracil | *IL10^-/-^* | Mouse | Urine | >8-24 | NMR | [4] |
| Uracil | *IL10^-/-^* | Mouse | Urine | >8-24 | NMR | [4] |
| Urea | DSS (A) | Mouse | Serum | >8-24 | GC-MS | [22] |
| Uric acid | DSS (A) | Mouse | Colon | >8-24 | GC-MS | [13] |
| Uric acid | DSS (A) | Mouse | Colon | >8-24 | GC-MS | [13] |
| Uridine 5'-diphosphate | DSS (A) | Mouse | Liver | >8-24 | ^1^H-NMR | [15] |
| Uridine 5'-monophosphate | DSS (A) | Mouse | Liver | >8-24 | ^1^H-NMR | [15] |
| Uridine 5'-triphosphate | DSS (A) | Mouse | Liver | >8-24 | ^1^H-NMR | [15] |
| Uridine TMS | *Winnie* | Mouse | Feces | >8-24 | GC-MS | [8] |
| Urobilinogen, L- | T-syn deficiency | Mouse | Colon (distal), cecum | 0-3 | UPLC/ToF-MS | [11] |
| Vaccenyl carnitine | DSS (C) | Mouse | Colon | >8-24 | LC-qTOF-MS | [14] |
| Valine | DSS (A) | Mouse | Plasma | >8-24 | ^1^H-NMR | [15] |
| Valine, L- | DSS (A) | Mouse | Serum | >8-24 | GC-MS | [13] |
| Valine, L- | DSS (A) | Mouse | Colon | >8-24 | GC-MS | [13] |
| Valine, L- | DSS (A) | Mouse | Serum | >8-24 | GC-MS | [13] |
| Valine, L- | DSS (A) | Mouse | Colon | >8-24 | GC-MS | [13] |
| Xanthine | T-syn deficiency | Mouse | Colon (distal), cecum | >8-24 | UPLC/ToF-MS | [11] |
| Xanthurenic acid | *IL10^-/-^* | Mouse | Urine | >3-8 | GC-MS | [16] |
| Xanthurenic acid | *IL10^-/-^* | Mouse | Urine | >3-8 | GC-MS | [16] |
| Xanthurenic acid | *IL10^-/-^* | Mouse | Urine | >3-8 | GC-MS | [16] |
| Xanthurenic acid | *IL10^-/-^* | Mouse | Urine | >3-8 | GC-MS | [16] |
| Xanthurenic acid | *IL10^-/-^* | Mouse | Urine | >8-24 | GC-MS | [16] |
| Xanthurenic acid | *IL10^-/-^* | Mouse | Urine | >8-24 | GC-MS | [16] |
| Xanthurenic acid | *IL10^-/-^* | Mouse | Urine | >8-24 | GC-MS | [16] |
| Xanthurenic acid | *IL10^-/-^* | Mouse | Urine | >8-24 | GC-MS | [16] |
| Xanthurenic acid | *IL10^-/-^* | Mouse | Urine | >3-8 | GC-MS | [19] |
| Xanthurenic acid | *IL10^-/-^* | Mouse | Urine | >3-8 | GC-MS | [19] |
| Xanthurenic acid | *IL10^-/-^* | Mouse | Urine | >3-8 | GC-MS | [19] |
| Xanthurenic acid | *IL10^-/-^* | Mouse | Urine | >8-24 | GC-MS | [19] |
| Xanthurenic acid | *IL10^-/-^* | Mouse | Urine | >8-24 | GC-MS | [19] |
| Xanthurenic acid | *IL10^-/-^* | Mouse | Urine | >8-24 | GC-MS | [19] |
| Xanthurenic acid | *IL10^-/-^* | Mouse | Urine | >8-24 | GC-MS | [19] |
| Xanthurenic acid | *IL10^-/-^* | Mouse | Urine | >8-24 | GC-MS | [19] |
| Xanthurenic acid | *IL10^-/-^* | Mouse | Urine | >8-24 | LC-MS | [26] |
| Xylose, D- (1MEOX) (4TMS) | *Winnie* | Mouse | Feces | >8-24 | GC-MS | [8] |
| Xylose, D- (1MEOX) (4TMS) | *Winnie* | Mouse | Feces | >8-24 | GC-MS | [8] |
| Zeatin | T-syn deficiency | Mouse | Colon (distal), cecum | 0-3 | UPLC/ToF-MS | [11] |
| ω-3 fatty acid | DSS (A) | Mouse | Plasma | >8-24 | ^1^H-NMR | [15] |

‘-‘ indicates that no metabolites were found to be significantly increased in the respective sample.

Model: (A): acute; ARE: AU-rich elements; (C): chronic; DSS: dextran sodium sulfate; H. hepaticus: Helicobacter hepaticus; IL: interleukin; T-syn: T-synthase; TNBS: 2,4,6-trinitrobenzenesulfonic acid; TNF: tumor necrosis factor; WT: wild-type. Platform: GC-MS: gas chromatography-mass spectrometry; HPLC-MS/MS: high performance liquid chromatography tandem mass spectrometry; LC-MS: liquid chromatography-mass spectrometry; LC-qTOF-MS: liquid chromatography quadropole time-of-flight mass spectrometry; MRS: magnetic resonance spectroscopy; NMR: nuclear magnetic resonance; SIFT-MS: selected-ion flow-tube mass spectrometry; UPLC-ESI-(q)TOF-MS: ultra performance liquid chromatography electrospray ionization (quadropole) time-of-flight mass spectrometry; UPLC-ESI (q)ToFMS: ultra performance liquid chromatography electrospray ionization (quadrupole) time-of-flight mass spectrometry; UPLC-MS: ultra performance liquid chromatography mass spectrometry; UPLC-MS/MS: ultra performance liquid chromatography tandem mass spectrometry; UPLC/ToFMS: ultra performance liquid chromatography time-of-flight mass spectrometry. GPL PCae: cholin glycerophospholipid with an ether bond; GPL PCaa: cholin glycerophospholipid; LysoPC: lysophosphatidylcholine; PC: phosphatidylcholine; PE: phosphatidylethanolamine; PG: phosphatidylglycerol; PS: phosphatidylserine.

**References**

1. Martin, F.P.J.; Lichti, P.; Bosco, N.; Brahmbhatt, V.; Oliveira, M.; Haller, D.; Benyacoub, J. Metabolic phenotyping of an adoptive transfer mouse model of experimental colitis and impact of dietary fish oil intake. *Journal of proteome research* **2015**, *14*, 1911-1919.

2. Willenberg, I.; Ostermann, A.I.; Giovannini, S.; Kershaw, O.; Von Keutz, A.; Steinberg, P.; Schebb, N.H. Effect of acute and chronic DSS induced colitis on plasma eicosanoid and oxylipin levels in the rat. *Prostaglandins and Other Lipid Mediators* **2015**, *120*, 155-160.

3. Baur, P.; Martin, F.P.; Gruber, L.; Bosco, N.; Brahmbhatt, V.; Collino, S.; Guy, P.; Montoliu, I.; Rozman, J.; Klingenspor, M., et al. Metabolic phenotyping of the Crohn's disease-like IBD etiopathology in the TNFDELTAARE/WT mouse model. *Journal of proteome research* **2011**, *10*, 5523-5535.

4. Murdoch, T.B.; Fu, H.; MacFarlane, S.; Sydora, B.C.; Fedorak, R.N.; Slupsky, C.M. Urinary metabolic profiles of inflammatory bowel disease in interleukin-10 gene-deficient mice. *Analytical Chemistry* **2008**, *80*, 5524-5531.

5. Vassilyadi, P.; Harding, S.V.; Nitschmann, E.; Wykes, L.J. Experimental colitis and malnutrition differentially affect the metabolism of glutathione and related sulfhydryl metabolites in different tissues. *European Journal of Nutrition* **2016**, *55*, 1769-1776.

6. Qi, Y.; Jiang, C.; Tanaka, N.; Krausz, K.W.; Brocker, C.N.; Fang, Z.Z.; Bredell, B.X.; Shah, Y.M.; Gonzalez, F.J. PPARalpha-dependent exacerbation of experimental colitis by the hypolipidemic drug fenofibrate. *American Journal of Physiology - Gastrointestinal and Liver Physiology* **2014**, *307*, G564-G573.

7. Wang, R.; Gu, X.; Dai, W.; Ye, J.; Lu, F.; Chai, Y.; Fan, G.; Gonzalez, F.J.; Duan, G.; Qi, Y. A lipidomics investigation into the intervention of celastrol in experimental colitis. *Mol Biosyst* **2016**, *12*, 1436-1444, doi:10.1039/c5mb00864f.

8. Robinson, A.M.; Gondalia, S.V.; Karpe, A.V.; Eri, R.; Beale, D.J.; Morrison, P.D.; Palombo, E.A.; Nurgali, K. Fecal microbiota and metabolome in a mouse model of spontaneous chronic colitis: Relevance to human inflammatory bowel disease. *Inflammatory bowel diseases* **2016**, *22*, 2767-2787.

9. Zhang, W.; Liao, J.; Li, H.; Dong, H.; Bai, H.; Yang, A.; Hammock, B.D.; Yang, G.Y. Reduction of inflammatory bowel disease-induced tumor development in IL-10 knockout mice with soluble epoxide hydrolase gene deficiency. *Molecular Carcinogenesis* **2013**, *52*, 726-738.

10. Kohnke, T.; Gomolka, B.; Bilal, S.; Zhou, X.; Sun, Y.; Rothe, M.; Baumgart, D.C.; Weylandt, K.H. Acetylsalicylic Acid reduces the severity of dextran sodium sulfate-induced colitis and increases the formation of anti-inflammatory lipid mediators. *BioMed research international* **2013**, *2013*, 748160, doi:10.1155/2013/748160.

11. Jacobs, J.P.; Lin, L.; Goudarzi, M.; Ruegger, P.; McGovern, D.P.B.; Fornace, A.J.; Borneman, J.; Xia, L.; Braun, J. Microbial, metabolomic, and immunologic dynamics in a relapsing genetic mouse model of colitis induced by T-synthase deficiency. *Gut microbes* **2017**, *8*, 1-16.

12. Lu, K.; Knutson, C.G.; Wishnok, J.S.; Fox, J.G.; Tannenbaum, S.R. Serum metabolomics in a helicobacter hepaticus mouse model of inflammatory bowel disease reveal important changes in the microbiome, serum peptides, and intermediary metabolism. *Journal of proteome research* **2012**, *11*, 4916-4926.

13. Shiomi, Y.; Nishiumi, S.; Ooi, M.; Hatano, N.; Shinohara, M.; Yoshie, T.; Kondo, Y.; Furumatsu, K.; Shiomi, H.; Kutsumi, H., et al. GCMS-based metabolomic study in mice with colitis induced by dextran sulfate sodium. *Inflammatory bowel diseases* **2011**, *17*, 2261-2274.

14. Liu, J.; Xiao, H.T.; Wang, H.S.; Mu, H.X.; Zhao, L.; Du, J.; Yang, D.; Wang, D.; Bian, Z.X.; Lin, S.H. Halofuginone reduces the inflammatory responses of DSS-induced colitis through metabolic reprogramming. *Mol Biosyst* **2016**, *12*, 2296-2303, doi:10.1039/c6mb00154h.

15. Dong, F.; Zhang, L.; Hao, F.; Tang, H.; Wang, Y. Systemic responses of mice to dextran sulfate sodium-induced acute ulcerative colitis using 1H NMR spectroscopy. *Journal of proteome research* **2013**, *12*, 2958-2966.

16. Lin, H.M.; Barnett, M.P.G.; Roy, N.C.; Joyce, N.I.; Zhu, S.; Armstrong, K.; Helsby, N.A.; Ferguson, L.R.; Rowan, D.D. Metabolomic analysis identifies inflammatory and noninflammatory metabolic effects of genetic modification in a mouse model of Crohn?s disease. *Journal of proteome research* **2010**, *9*, 1965-1975.

17. Schicho, R.; Nazyrova, A.; Shaykhutdinov, R.; Duggan, G.; Vogel, H.J.; Storr, M. Quantitative metabolomic profiling of serum and urine in DSS-induced ulcerative colitis of mice by 1H NMR spectroscopy. *Journal of proteome research* **2010**, *9*, 6265-6273.

18. Zhang, X.; Choi, F.F.; Zhou, Y.; Leung, F.P.; Tan, S.; Lin, S.; Xu, H.; Jia, W.; Sung, J.J.; Cai, Z., et al. Metabolite profiling of plasma and urine from rats with TNBS-induced acute colitis using UPLC-ESI-QTOF-MS-based metabonomics--a pilot study. *The FEBS journal* **2012**, *279*, 2322-2338, doi:10.1111/j.1742-4658.2012.08612.x.

19. Lin, H.M.; Edmunds, S.J.; Helsby, N.A.; Ferguson, L.R.; Rowan, D.D. Nontargeted urinary metabolite profiling of a mouse model of crohn's disease. *Journal of proteome research* **2009**, *8*, 2045-2057.

20. Qu, C.; Yuan, Z.W.; Yu, X.T.; Huang, Y.F.; Yang, G.H.; Chen, J.N.; Lai, X.P.; Su, Z.R.; Zeng, H.F.; Xie, Y., et al. Patchouli alcohol ameliorates dextran sodium sulfate-induced experimental colitis and suppresses tryptophan catabolism. *Pharmacological research* **2017**, *121*, 70-82.

21. Martin, F.P.J.; Rezzi, S.; Montoliu, I.; Philippe, D.; Tornier, L.; Messlik, A.; Holzlwimmer, G.; Baur, P.; Quintanilla-Fend, L.; Loh, G., et al. Metabolic assessment of gradual development of moderate experimental colitis in IL-10 deficient mice. *Journal of proteome research* **2009**, *8*, 2376-2387.

22. Gu, X.; Song, Y.; Chai, Y.; Lu, F.; Gonzalez, F.J.; Fan, G.; Qi, Y. GC-MS metabolomics on PPARalpha-dependent exacerbation of colitis. *Molecular bioSystems* **2015**, *11*, 1329-1337.

23. Hou, W.; Zhong, D.; Zhang, P.; Li, Y.; Lin, M.; Liu, G.; Yao, M.; Liao, Q.; Xie, Z. A strategy for the targeted metabolomics analysis of 11 gut microbiota-host co-metabolites in rat serum, urine and feces by ultra high performance liquid chromatography-tandem mass spectrometry. *Journal of Chromatography A* **2016**, *1429*, 207-217.

24. Hong, Y.S.; Ahn, Y.T.; Park, J.C.; Lee, J.H.; Lee, H.; Huh, C.S.; Kim, D.H.; Ryu, D.H.; Hwang, G.S. 1H NMR-based metabonomic assessment of probiotic effects in a colitis mouse model. *Archives of pharmacal research* **2010**, *33*, 1091-1101.

25. Kominsky, D.J.; Keely, S.; MacManus, C.F.; Glover, L.E.; Scully, M.; Collins, C.B.; Bowers, B.E.; Campbell, E.L.; Colgan, S.P. An endogenously anti-inflammatory role for methylation in mucosal inflammation identified through metabolite profiling. *J Immunol* **2011**, *186*, 6505-6514, doi:10.4049/jimmunol.1002805.

26. Otter, D.; Cao, M.; Lin, H.M.; Fraser, K.; Edmunds, S.; Lane, G.; Rowan, D. Identification of urinary biomarkers of colon inflammation in IL10-/- mice using Short-Column LCMS metabolomics. *Journal of biomedicine & biotechnology* **2011**, *2011*, 974701, doi:10.1155/2011/974701.
