# Supplementary material for: Translational Potential of Metabolomics on Animal Models of Inflammatory Bowel Disease—A Systematic Critical Review
Source: Int J Mol Sci. 2020 May 29;21(11):3856. doi: 10.3390/ijms21113856 (PMC7312423; doi:10.3390/ijms21113856)
Supplement: Supplementary file 1 [file ijms-21-03856-s001.zip › Supplementary Table S8_resubmission_proofread.docx]

**Supplementary Table S8: Metabolites significantly decreased in IBD vs healthy controls in animal models**

| **Metabolites ↓ in IBD** | **Model** | **Species** | **Sample type** | **Age group** | **Platform** | **Reference** |
| --- | --- | --- | --- | --- | --- | --- |
| - | Adoptive transfer | Mouse | Plasma | >8-24 | ^1^H-NMR | [1] |
| - | DSS (A) | Mouse | Colon | >3-8 | HPLC-MS/MS | [2] |
| - | *IL10^-/-^* | Mouse | Plasma | >24 | LC/MS-MS | [3] |
| - | *IL10^-/-^* | Mouse | Plasma | >24 | LC/MS-MS | [3] |
| - | DSS (A) | Rat | Plasma | >3-8 | LC-MS | [4] |
| - | DSS (A) | Rat | Plasma | >8-24 | LC-MS | [4] |
| - | DSS (A) | Rat | Plasma | >8-24 | LC-MS | [4] |
| - | DSS (C) | Rat | Plasma | >8-24 | LC-MS | [4] |
| - | DSS (C) | Rat | Plasma | >8-24 | LC-MS | [4] |
| - | DSS (C) | Rat | Plasma | >8-24 | LC-MS | [4] |
| - | *TNF*^ΔARE/WT^ | Mouse | Ileum (distal) | >3-8 | LC-MS | [5] |
| - | *TNF*^ΔARE/WT^ | Mouse | Ileum (distal) | >3-8 | LC-MS | [5] |
| - | *IL10^-/-^* | Mouse | Plasma | >8-24 | LC-MS | [6] |
| - | *IL10^-/-^* | Mouse | Urine | >3-8 | NMR | [7] |
| - | *IL10^-/-^* | Mouse | Urine | >3-8 | NMR | [7] |
| - | *IL10^-/-^* | Mouse | Urine | >3-8 | NMR | [7] |
| - | *IL10^-/-^* | Mouse | Urine | >8-24 | NMR | [7] |
| - | DSS (A) | Piglet | Liver | 0-3 | UHPLC/MS-MS | [8] |
| - | DSS (A) | Piglet | Spiral colon | 0-3 | UHPLC/MS-MS | [8] |
| - | DSS (A) | Piglet | Small intestine | 0-3 | UHPLC/MS-MS | [8] |
| - | DSS (A) | Piglet | Longissimus dorsi | 0-3 | UHPLC/MS-MS | [8] |
| - | DSS (A) | Piglet | Masseter | 0-3 | UHPLC/MS-MS | [8] |
| - | DSS (A) | Mouse | Serum | >8-24 | UPLC-ESI-qTOFMS | [9] |
| - | DSS (A) | Mouse | Colon | >8-24 | UPLC-ESI-qTOFMS | [9] |
| (R)-3-((R)-3-Hydroxybutanoyloxy)butanoate | T-syn deficiency | Mouse | Colon (distal), cecum | >3-8 | UPLC/ToF-MS | [10] |
| 11,12-Dihydroxy eicosatrienoic acid | *TNF*^ΔARE/WT^ | Mouse | Ileum (distal) | >8-24 | LC-MS | [5] |
| 12α-Hydroxy-3-oxocholadienic acid | TNBS | Rat | Urine | ? | UPLC-ESI-qTOF-MS | [11] |
| 12α-Hydroxy-3-oxocholadienic acid | TNBS | Rat | Plasma | ? | UPLC-ESI-qTOF-MS | [11] |
| 13-Hydroxy octadecadienoic acid | *TNF*^ΔARE/WT^ | Mouse | Ileum (distal) | >8-24 | LC-MS | [5] |
| 13-Hydroxy octadecadienoic acid | *TNF*^ΔARE/WT^ | Mouse | Ileum (distal) | >8-24 | LC-MS | [5] |
| 14,15 -Dihydroxy eicosatrienoic acid | *TNF*^ΔARE/WT^ | Mouse | Ileum (distal) | >8-24 | LC-MS | [5] |
| 19,20-Dihydroxydocosapentaenoate | H. hepaticus | Mouse | Serum | >8-24 | UPLC-ESI-TOF-MS | [12] |
| 2-(4-hydroxyphenyl)propanoic Acid | DSS (A) | Mouse | Urine | >8-24 | ^1^H-NMR | [13] |
| 2-(4-hydroxyphenyl)propanoic Acid | DSS (A) | Mouse | Urine | >8-24 | ^1^H-NMR | [13] |
| 2-(4-hydroxyphenyl)propanoic Acid | DSS (A) | Mouse | Urine | >8-24 | ^1^H-NMR | [13] |
| 2-(4-hydroxyphenyl)propanoic Acid | DSS (A) | Mouse | Urine | >8-24 | ^1^H-NMR | [13] |
| 2-(6'-Methylthio)hexylmalic acid | T-syn deficiency | Mouse | Colon (distal), cecum | >3-8 | UPLC/ToF-MS | [10] |
| 2-Aminooctanoic acid, DL- | H. hepaticus | Mouse | Serum | >24 | UPLC-ESI-TOF-MS | [12] |
| 2-Hydroxyadipic acid | *IL10^-/-^* | Mouse | Urine | >3-8 | GC-MS | [6] |
| 2-Hydroxyadipic acid | *IL10^-/-^* | Mouse | Urine | >3-8 | GC-MS | [6] |
| 2-Hydroxyadipic acid | *IL10^-/-^* | Mouse | Urine | >3-8 | GC-MS | [6] |
| 2-Hydroxyadipic acid | *IL10^-/-^* | Mouse | Urine | >3-8 | GC-MS | [6] |
| 2-Hydroxyadipic acid | *IL10^-/-^* | Mouse | Urine | >8-24 | GC-MS | [6] |
| 2-Hydroxyadipic acid | *IL10^-/-^* | Mouse | Urine | >8-24 | GC-MS | [6] |
| 2-Hydroxyadipic acid | *IL10^-/-^* | Mouse | Urine | >8-24 | GC-MS | [6] |
| 2-Hydroxyadipic acid | *IL10^-/-^* | Mouse | Urine | >8-24 | GC-MS | [6] |
| 2-Hydroxyadipic acid | *IL10^-/-^* | Mouse | Urine | >3-8 | GC-MS | [14] |
| 2-Hydroxyadipic acid | *IL10^-/-^* | Mouse | Urine | >3-8 | GC-MS | [14] |
| 2-Hydroxyadipic acid | *IL10^-/-^* | Mouse | Urine | >3-8 | GC-MS | [14] |
| 2-Hydroxyadipic acid | *IL10^-/-^* | Mouse | Urine | >8-24 | GC-MS | [14] |
| 2-Hydroxyadipic acid | *IL10^-/-^* | Mouse | Urine | >8-24 | GC-MS | [14] |
| 2-Hydroxyadipic acid | *IL10^-/-^* | Mouse | Urine | >8-24 | GC-MS | [14] |
| 2-Hydroxyadipic acid | *IL10^-/-^* | Mouse | Urine | >8-24 | GC-MS | [14] |
| 2-Hydroxyadipic acid | *IL10^-/-^* | Mouse | Urine | >8-24 | GC-MS | [14] |
| 2-Hydroxyglutaric acid | *IL10^-/-^* | Mouse | Urine | >3-8 | GC-MS | [6] |
| 2-Hydroxyglutaric acid | *IL10^-/-^* | Mouse | Urine | >3-8 | GC-MS | [6] |
| 2-Hydroxyglutaric acid | *IL10^-/-^* | Mouse | Urine | >3-8 | GC-MS | [6] |
| 2-Hydroxyglutaric acid | *IL10^-/-^* | Mouse | Urine | >3-8 | GC-MS | [6] |
| 2-Hydroxyglutaric acid | *IL10^-/-^* | Mouse | Urine | >8-24 | GC-MS | [6] |
| 2-Hydroxyglutaric acid | *IL10^-/-^* | Mouse | Urine | >8-24 | GC-MS | [6] |
| 2-Hydroxyglutaric acid | *IL10^-/-^* | Mouse | Urine | >8-24 | GC-MS | [6] |
| 2-Hydroxyglutaric acid | *IL10^-/-^* | Mouse | Urine | >8-24 | GC-MS | [6] |
| 2-Hydroxyglutaric acid | *IL10^-/-^* | Mouse | Urine | >3-8 | GC-MS | [14] |
| 2-Hydroxyglutaric acid | *IL10^-/-^* | Mouse | Urine | >3-8 | GC-MS | [14] |
| 2-Hydroxyglutaric acid | *IL10^-/-^* | Mouse | Urine | >3-8 | GC-MS | [14] |
| 2-Hydroxyglutaric acid | *IL10^-/-^* | Mouse | Urine | >8-24 | GC-MS | [14] |
| 2-Hydroxyglutaric acid | *IL10^-/-^* | Mouse | Urine | >8-24 | GC-MS | [14] |
| 2-Hydroxyglutaric acid | *IL10^-/-^* | Mouse | Urine | >8-24 | GC-MS | [14] |
| 2-Hydroxyglutaric acid | *IL10^-/-^* | Mouse | Urine | >8-24 | GC-MS | [14] |
| 2-Hydroxyglutaric acid | *IL10^-/-^* | Mouse | Urine | >8-24 | GC-MS | [14] |
| 2-Hydroxyisovaleric acid | DSS (A) | Mouse | Colon | >8-24 | GC-MS | [15] |
| 2-Hydroxyphenylacetic acid | DSS (A) | Mouse | Serum | >8-24 | GC-MS | [15] |
| 2-Hydroxyphenylacetic acid | DSS (A) | Mouse | Serum | >8-24 | GC-MS | [15] |
| 2-Oxoglutarate | DSS (A) | Mouse | Serum | >3-8 | ^1^H-NMR | [16] |
| 2-Oxoglutarate | *IL10^-/-^* | Mouse | Urine | >8-24 | NMR | [7] |
| 2-Oxoglutarate | *IL10^-/-^* | Mouse | Urine | >8-24 | NMR | [7] |
| 2-Piperidinecarboxylic acid, 1-(trimethylsilyl)-, trimethylsilyl ester | *Winnie* | Mouse | Feces | >8-24 | GC-MS | [17] |
| 2-trans,6-trans-Farnesal | T-syn deficiency | Mouse | Colon (distal), cecum | >8-24 | UPLC/ToF-MS | [10] |
| 3,6-Epoxydodecanedioic acid | DSS (A) | Mouse | Serum | >8-24 | GC-MS | [15] |
| 3-Hydroxydodecanedioic acid | DSS (A) | Mouse | Colon | >8-24 | GC-MS | [15] |
| 3-Hydroxydodecanedioic acid | DSS (A) | Mouse | Colon | >8-24 | GC-MS | [15] |
| 3-Hydroxysebacic acid | DSS (A) | Mouse | Colon | >8-24 | GC-MS | [15] |
| 3-Hydroxyvaleric acid | DSS (A) | Mouse | Colon | >8-24 | GC-MS | [15] |
| 3-Indolebutyric acid | H. hepaticus | Mouse | Serum | >24 | UPLC-ESI-TOF-MS | [12] |
| 3-Indolepropionic acid | H. hepaticus | Mouse | Serum | >8-24 | UPLC-ESI-TOF-MS | [12] |
| 3-Indolepropionic acid | H. hepaticus | Mouse | Serum | >24 | UPLC-ESI-TOF-MS | [12] |
| 3-Methyldioxyindole | T-syn deficiency | Mouse | Colon (distal), cecum | >3-8 | UPLC/ToF-MS | [10] |
| 3S-Bromobutanoic acid | DSS (C) | Mouse | Colon | >8-24 | LC-qTOF-MS | [18] |
| 3α,7α,12α-Trihydroxy-5β-cholestanoate | T-syn deficiency | Mouse | Colon (distal), cecum | >3-8 | UPLC/ToF-MS | [10] |
| 4-(2-Aminophenyl-2,4-dioxobutanoate | T-syn deficiency | Mouse | Colon (distal), cecum | >3-8 | UPLC/ToF-MS | [10] |
| 4-Cresol glucuronide | DSS (A) | Mouse | Urine | >8-24 | ^1^H-NMR | [13] |
| 4-Cresol glucuronide | DSS (A) | Mouse | Urine | >8-24 | ^1^H-NMR | [13] |
| 4-Cresol glucuronide | DSS (A) | Mouse | Urine | >8-24 | ^1^H-NMR | [13] |
| 4-Cresol glucuronide | DSS (A) | Mouse | Urine | >8-24 | ^1^H-NMR | [13] |
| 4-Cresol glucuronide | DSS (A) | Mouse | Urine | >8-24 | ^1^H-NMR | [13] |
| 4-Cresol sulfate | DSS (A) | Mouse | Urine | >8-24 | ^1^H-NMR | [13] |
| 4-Cresol sulfate | DSS (A) | Mouse | Urine | >8-24 | ^1^H-NMR | [13] |
| 4-Cresol sulfate | DSS (A) | Mouse | Urine | >8-24 | ^1^H-NMR | [13] |
| 4-Cresol sulfate | DSS (A) | Mouse | Urine | >8-24 | ^1^H-NMR | [13] |
| 4-Cresol sulfate | DSS (A) | Mouse | Urine | >8-24 | ^1^H-NMR | [13] |
| 4-Hydroxyphenyllactic acid | DSS (A) | Mouse | Serum | >8-24 | GC-MS | [15] |
| 4-Hydroxyphenyllactic acid | *IL10^-/-^* | Mouse | Urine | >8-24 | GC-MS | [6] |
| 4-Hydroxyphenyllactic acid | *IL10^-/-^* | Mouse | Urine | >8-24 | GC-MS | [6] |
| 4-Hydroxyphenyllactic acid | *IL10^-/-^* | Mouse | Urine | >8-24 | GC-MS | [6] |
| 5,6-Dihydroxy eicosatrienoic acid | *TNF*^ΔARE/WT^ | Mouse | Ileum (distal) | >8-24 | LC-MS | [5] |
| 5,8,11-Eicosatrienoic acid | H. hepaticus | Mouse | Serum | >24 | UPLC-ESI-TOF-MS | [12] |
| 5-Hydroxyindoleacetic acid | *IL10^-/-^* | Mouse | Urine | >3-8 | GC-MS | [6] |
| 5-Hydroxyindoleacetic acid | *IL10^-/-^* | Mouse | Urine | >3-8 | GC-MS | [6] |
| 5-Hydroxyindoleacetic acid | *IL10^-/-^* | Mouse | Urine | >8-24 | GC-MS | [6] |
| 5-Hydroxyindoleacetic acid | *IL10^-/-^* | Mouse | Urine | >8-24 | GC-MS | [6] |
| 5-Hydroxyindoleacetic acid | *IL10^-/-^* | Mouse | Urine | >8-24 | GC-MS | [6] |
| 5-Hydroxyindoleacetic acid | H. hepaticus | Mouse | Serum | >8-24 | UPLC-ESI-TOF-MS | [12] |
| 5-Hydroxyindoleacetic acid | H. hepaticus | Mouse | Serum | >24 | UPLC-ESI-TOF-MS | [12] |
| 7-Hydroxymethyl-12-methylbenz[a]anthracene sulfate | T-syn deficiency | Mouse | Colon (distal), cecum | >3-8 | UPLC/ToF-MS | [10] |
| 7-Hydroxymethyl-12-methylbenz[a]anthracene sulfate | T-syn deficiency | Mouse | Colon (distal), cecum | >8-24 | UPLC/ToF-MS | [10] |
| 8,9-Dihydroxy eicosatrienoic acid | *TNF*^ΔARE/WT^ | Mouse | Ileum (distal) | >8-24 | LC-MS | [5] |
| 9-Hydroxy eicosatetraenoic acid | *TNF*^ΔARE/WT^ | Mouse | Ileum (distal) | >8-24 | LC-MS | [5] |
| 9-Hydroxy octadecadienoic acid | *TN*F^ΔARE/WT^ | Mouse | Ileum (distal) | >8-24 | LC-MS | [5] |
| Acetate | DSS (A) | Mouse | Plasma | >8-24 | ^1^H-NMR | [13] |
| Acetate | DSS (A) | Mouse | Serum | >3-8 | ^1^H-NMR | [16] |
| Acetylcarnitine | DSS (C) | Mouse | Spleen | >8-24 | LC-qTOF-MS | [18] |
| Acetylglutamic acid, N- | H. hepaticus | Mouse | Serum | >24 | UPLC-ESI-TOF-MS | [12] |
| Acetyl-L-phenylalanine, N- | H. hepaticus | Mouse | Serum | >24 | UPLC-ESI-TOF-MS | [12] |
| Aconitic acid, cis- | *IL10^-/-^* | Mouse | Urine | >3-8 | GC-MS | [6] |
| Aconitic acid, cis- | *IL10^-/-^* | Mouse | Urine | >3-8 | GC-MS | [6] |
| Aconitic acid, cis- | *IL10^-/-^* | Mouse | Urine | >8-24 | GC-MS | [6] |
| Aconitic acid, cis- | *IL10^-/-^* | Mouse | Urine | >8-24 | GC-MS | [6] |
| Aconitic acid, cis- | *IL10^-/-^* | Mouse | Urine | >8-24 | GC-MS | [6] |
| Aconitic acid, cis- | *IL10^-/-^* | Mouse | Urine | >3-8 | GC-MS | [14] |
| Aconitic acid, cis- | *IL10^-/-^* | Mouse | Urine | >8-24 | GC-MS | [14] |
| Acylcarnitine C10 | *TNF*^ΔARE/WT^ | Mouse | Ileum (distal) | >8-24 | LC-MS | [5] |
| Acylcarnitine C10 | *TNF*^ΔARE/WT^ | Mouse | Ileum (distal) | >8-24 | LC-MS | [5] |
| Acylcarnitine C10:1 | *TNF*^ΔARE/WT^ | Mouse | Ileum (distal) | >8-24 | LC-MS | [5] |
| Acylcarnitine C10:1 | *TNF*^ΔARE/WT^ | Mouse | Ileum (distal) | >8-24 | LC-MS | [5] |
| Acylcarnitine C10:2 | *TNF*^ΔARE/WT^ | Mouse | Ileum (distal) | >8-24 | LC-MS | [5] |
| Acylcarnitine C12 | *TNF*^ΔARE/WT^ | Mouse | Ileum (distal) | >8-24 | LC-MS | [5] |
| Acylcarnitine C12:1 | *TNF*^ΔARE/WT^ | Mouse | Ileum (distal) | >8-24 | LC-MS | [5] |
| Acylcarnitine C12:1 | *TNF*^ΔARE/WT^ | Mouse | Ileum (distal) | >8-24 | LC-MS | [5] |
| Acylcarnitine C12-DC | *TNF*^ΔARE/WT^ | Mouse | Ileum (distal) | >8-24 | LC-MS | [5] |
| Acylcarnitine C12-DC | *TNF*^ΔARE/WT^ | Mouse | Ileum (distal) | >8-24 | LC-MS | [5] |
| Acylcarnitine C14 | *TNF*^ΔARE/WT^ | Mouse | Ileum (distal) | >8-24 | LC-MS | [5] |
| Acylcarnitine C14 | *TNF*^ΔARE/WT^ | Mouse | Ileum (distal) | >8-24 | LC-MS | [5] |
| Acylcarnitine C14:1 | *TNF*^ΔARE/WT^ | Mouse | Ileum (distal) | >8-24 | LC-MS | [5] |
| Acylcarnitine C14:1 | *TNF*^ΔARE/WT^ | Mouse | Ileum (distal) | >8-24 | LC-MS | [5] |
| Acylcarnitine C14:1-OH | *TNF*^ΔARE/WT^ | Mouse | Ileum (distal) | >8-24 | LC-MS | [5] |
| Acylcarnitine C14:2-OH | *TNF*^ΔARE/WT^ | Mouse | Ileum (distal) | >8-24 | LC-MS | [5] |
| Acylcarnitine C14:2-OH | *TNF*^ΔARE/WT^ | Mouse | Ileum (distal) | >8-24 | LC-MS | [5] |
| Acylcarnitine C16:1 | *TNF*^ΔARE/WT^ | Mouse | Ileum (distal) | >8-24 | LC-MS | [5] |
| Acylcarnitine C16:1 | *TNF*^ΔARE/WT^ | Mouse | Ileum (distal) | >8-24 | LC-MS | [5] |
| Acylcarnitine C16:1-OH | *TNF*^ΔARE/WT^ | Mouse | Ileum (distal) | >8-24 | LC-MS | [5] |
| Acylcarnitine C3-OH | *TNF*^ΔARE/WT^ | Mouse | Ileum (distal) | >8-24 | LC-MS | [5] |
| Acylcarnitine C4-OH (C3-DC) | *TNF*^ΔARE/WT^ | Mouse | Ileum (distal) | >8-24 | LC-MS | [5] |
| Acylcarnitine C5-M-DC | *TNF*^ΔARE/WT^ | Mouse | Ileum (distal) | >8-24 | LC-MS | [5] |
| Acylcarnitine C5-M-DC | *TNF*^ΔARE/WT^ | Mouse | Ileum (distal) | >8-24 | LC-MS | [5] |
| Acylcarnitine C6 (C4:1-DC) | *TNF*^ΔARE/WT^ | Mouse | Ileum (distal) | >8-24 | LC-MS | [5] |
| Acylcarnitine C6 (C4:1-DC) | *TNF*^ΔARE/WT^ | Mouse | Ileum (distal) | >3-8 | LC-MS | [5] |
| Acylcarnitine C6:1 | *TNF*^ΔARE/WT^ | Mouse | Ileum (distal) | >8-24 | LC-MS | [5] |
| Acylcarnitine C8 | *TNF*^ΔARE/WT^ | Mouse | Ileum (distal) | >8-24 | LC-MS | [5] |
| Acylcarnitine C8:1 | *TNF*^ΔARE/WT^ | Mouse | Ileum (distal) | >8-24 | LC-MS | [5] |
| Acylcarnitine C9 | *TNF*^ΔARE/WT^ | Mouse | Ileum (distal) | >8-24 | LC-MS | [5] |
| Adenine | H. hepaticus | Mouse | Serum | >24 | UPLC-ESI-TOF-MS | [12] |
| Adenosine 5'-monophosphate | DSS (A) | Mouse | Colon | >8-24 | ^1^H-NMR | [13] |
| Adenosylhomocysteine, S- | H. hepaticus | Mouse | Serum | >24 | UPLC-ESI-TOF-MS | [12] |
| Adipate | DSS (A) | Mouse | Urine | >8-24 | ^1^H-NMR | [13] |
| Adipate | DSS (A) | Mouse | Urine | >8-24 | ^1^H-NMR | [13] |
| Adipate | DSS (A) | Mouse | Urine | >8-24 | ^1^H-NMR | [13] |
| Adipate | DSS (A) | Mouse | Urine | >8-24 | ^1^H-NMR | [13] |
| Adipate | DSS (A) | Mouse | Urine | >8-24 | ^1^H-NMR | [13] |
| Adrenic acid | H. hepaticus | Mouse | Serum | >24 | UPLC-ESI-TOF-MS | [12] |
| Agmatine | T-syn deficiency | Mouse | Colon (distal), cecum | 0-3 | UPLC/ToF-MS | [10] |
| Alanine | Adoptive transfer | Mouse | Urine | >8-24 | ^1^H-NMR | [1] |
| Alanine | DSS (A) | Mouse | Serum | >3-8 | ^1^H-NMR | [16] |
| Aminoimidazole ribotide | T-syn deficiency | Mouse | Colon (distal), cecum | 0-3 | UPLC/ToF-MS | [10] |
| Arachidonic acid | *TNF*^ΔARE/WT^ | Mouse | Ileum (distal) | >8-24 | LC-MS | [5] |
| Arachidonic acid | H. hepaticus | Mouse | Serum | >8-24 | UPLC-ESI-TOF-MS | [12] |
| Arachidonic acid | H. hepaticus | Mouse | Serum | >24 | UPLC-ESI-TOF-MS | [12] |
| Aspartate | DSS (A) | Mouse | Feces | >3-8 | ^1^H-NMR | [19] |
| Aspartyl-L-proline | H. hepaticus | Mouse | Serum | >24 | UPLC-ESI-TOF-MS | [12] |
| Azelate | DSS (A) | Mouse | Urine | >8-24 | ^1^H-NMR | [13] |
| Azelate | DSS (A) | Mouse | Urine | >8-24 | ^1^H-NMR | [13] |
| Azelate | DSS (A) | Mouse | Urine | >8-24 | ^1^H-NMR | [13] |
| Azelate | DSS (A) | Mouse | Urine | >8-24 | ^1^H-NMR | [13] |
| Betaine | DSS (A) | Mouse | Serum | >3-8 | ^1^H-NMR | [16] |
| Betaine | DSS (A) | Mouse | Colon | >8-24 | NMR(1H, 1C, 1P) | [20] |
| Butyrate | DSS (A) | Mouse | Urine | >8-24 | ^1^H-NMR | [13] |
| Butyrate | TNBS | Rat | Urine | ? | UPLC-MS/MS | [21] |
| Butyrate | TNBS | Rat | Feces | ? | UPLC-MS/MS | [21] |
| Butyrate | TNBS | Rat | Feces | ? | UPLC-MS/MS | [21] |
| Butyric acid, 4-amino- (2TMS) | *Winnie* | Mouse | Feces | >8-24 | GC-MS | [17] |
| Cadaverine 4TMS | *Winnie* | Mouse | Feces | >8-24 | GC-MS | [17] |
| Carnitine | DSS (A) | Mouse | Serum | >3-8 | ^1^H-NMR | [16] |
| CEHC glucuronide, α- | *IL10^-/-^* | Mouse | Urine | >8-24 | LC-MS | [22] |
| Chenodeoxycholate | T-syn deficiency | Mouse | Colon (distal), cecum | >8-24 | UPLC/ToF-MS | [10] |
| Cholestane-3,7,12,24,25-pentol | TNBS | Rat | Plasma | ? | UPLC-ESI-qTOF-MS | [11] |
| Cholic acid | T-syn deficiency | Mouse | Colon (distal), cecum | >3-8 | UPLC/ToF-MS | [10] |
| Cholic acid | T-syn deficiency | Mouse | Colon (distal), cecum | >8-24 | UPLC/ToF-MS | [10] |
| Cholines (Total) | DSS (A) | Mouse | Colon | >8-24 | NMR(1H, 1C, 1P) | [20] |
| Citrate | DSS (A) | Mouse | Serum | >3-8 | ^1^H-NMR | [16] |
| Citrate | *IL10^-/-^* | Mouse | Urine | >8-24 | NMR | [7] |
| Citric acid | DSS (A) | Mouse | Serum | >8-24 | GC-MS | [15] |
| Citric acid | DSS (A) | Mouse | Serum | >8-24 | GC-MS | [15] |
| Citric acid | H. hepaticus | Mouse | Serum | >8-24 | UPLC-ESI-TOF-MS | [12] |
| Citric acid | H. hepaticus | Mouse | Serum | >24 | UPLC-ESI-TOF-MS | [12] |
| Citric acid | DSS (A) | Mouse | Plasma | >8-24 | UPLC-MS | [23] |
| Coumaric acid | H. hepaticus | Mouse | Serum | >8-24 | UPLC-ESI-TOF-MS | [12] |
| Creatine δ 3.03 | *IL10^-/-^* | Mouse | Plasma | >8-24 | ^1^H-NMR | [24] |
| Cysteine | DSS (A) | Piglet | Colon (distal), red blood cells | 0-3 | UHPLC/MS-MS | [8] |
| Cysteineglutathione disulfide | H. hepaticus | Mouse | Serum | >24 | UPLC-ESI-TOF-MS | [12] |
| Cytidine | DSS (A) | Mouse | Spleen | >8-24 | ^1^H-NMR | [13] |
| Cytidine 5'-diphosphate | DSS (A) | Mouse | Colon | >8-24 | ^1^H-NMR | [13] |
| Deoxyinosine | H. hepaticus | Mouse | Serum | >24 | UPLC-ESI-TOF-MS | [12] |
| Diacylglycerol | *TNF*^ΔARE/WT^ | Mouse | Ileum (distal) | >8-24 | ^1^H-NMR | [5] |
| Dihydroxyphenylbenzoic acid | H. hepaticus | Mouse | Serum | >8-24 | UPLC-ESI-TOF-MS | [12] |
| Dimethylglycine δ 2.93 | *IL10^-/-^* | Mouse | Plasma | 0-3 | ^1^H-NMR | [24] |
| Dimethylglycine δ 2.93 | *IL10^-/-^* | Mouse | Plasma | >8-24 | ^1^H-NMR | [24] |
| Docosahexaenoic acid | H. hepaticus | Mouse | Serum | >8-24 | UPLC-ESI-TOF-MS | [12] |
| Docosahexaenoic acid | H. hepaticus | Mouse | Serum | >24 | UPLC-ESI-TOF-MS | [12] |
| Docosapentaenoic acid | T-syn deficiency | Mouse | Colon (distal), cecum | >3-8 | UPLC/ToF-MS | [10] |
| Docosapentaenoic acid | T-syn deficiency | Mouse | Colon (distal), cecum | >8-24 | UPLC/ToF-MS | [10] |
| Docosapentaenoic acid | H. hepaticus | Mouse | Serum | >8-24 | UPLC-ESI-TOF-MS | [12] |
| Docosapentaenoic acid | H. hepaticus | Mouse | Serum | >24 | UPLC-ESI-TOF-MS | [12] |
| Docosatetraenoic acid | T-syn deficiency | Mouse | Colon (distal), cecum | >3-8 | UPLC/ToF-MS | [10] |
| Docosatetraenoic acid | T-syn deficiency | Mouse | Colon (distal), cecum | >8-24 | UPLC/ToF-MS | [10] |
| Eicosapentaenoic acid | H. hepaticus | Mouse | Serum | >8-24 | UPLC-ESI-TOF-MS | [12] |
| Eicosapentaenoic acid | H. hepaticus | Mouse | Serum | >24 | UPLC-ESI-TOF-MS | [12] |
| Emetine | T-syn deficiency | Mouse | Colon (distal), cecum | >3-8 | UPLC/ToF-MS | [10] |
| Ergothioneine | H. hepaticus | Mouse | Serum | >8-24 | UPLC-ESI-TOF-MS | [12] |
| Ergothioneine | H. hepaticus | Mouse | Serum | >24 | UPLC-ESI-TOF-MS | [12] |
| Estrone | T-syn deficiency | Mouse | Colon (distal), cecum | >8-24 | UPLC/ToF-MS | [10] |
| Fructose 6-phosphate, D- | T-syn deficiency | Mouse | Colon (distal), cecum | 0-3 | UPLC/ToF-MS | [10] |
| Fucose 1-phosphate, β-L- | DSS (C) | Mouse | Colon | >8-24 | LC-qTOF-MS | [18] |
| Fumarate | DSS (A) | Mouse | Liver | >8-24 | ^1^H-NMR | [13] |
| Fumarate | DSS (A) | Mouse | Serum | >3-8 | ^1^H-NMR | [16] |
| Fumarate | *IL10^-/-^* | Mouse | Urine | >8-24 | NMR | [7] |
| Fumarate δ 6.51 | *IL10^-/-^* | Mouse | Plasma | 0-3 | ^1^H-NMR | [24] |
| Fumaric acid | DSS (A) | Mouse | Serum | >8-24 | GC-MS | [15] |
| Gentisic acid | H. hepaticus | Mouse | Serum | >24 | UPLC-ESI-TOF-MS | [12] |
| Glucose | DSS (A) | Mouse | Plasma | >8-24 | ^1^H-NMR | [13] |
| Glucose | DSS (A) | Mouse | Liver | >8-24 | ^1^H-NMR | [13] |
| Glucose | DSS (A) | Mouse | Serum | >3-8 | ^1^H-NMR | [16] |
| Glucose | DSS (A) | Mouse | Serum | >8-24 | GC-MS | [25] |
| Glucose | *IL10^-/-^* | Mouse | Urine | >3-8 | GC-MS | [6] |
| Glucose | *IL10^-/-^* | Mouse | Urine | >3-8 | GC-MS | [6] |
| Glucose | *IL10^-/-^* | Mouse | Urine | >3-8 | GC-MS | [6] |
| Glucose | *IL10^-/-^* | Mouse | Urine | >8-24 | GC-MS | [6] |
| Glucose | *IL10^-/-^* | Mouse | Urine | >8-24 | GC-MS | [6] |
| Glucose | *IL10^-/-^* | Mouse | Urine | >8-24 | GC-MS | [6] |
| Glucose | *IL10^-/-^* | Mouse | Urine | >8-24 | GC-MS | [6] |
| Glucose | *IL10^-/-^* | Mouse | Urine | >3-8 | GC-MS | [14] |
| Glucose | *IL10^-/-^* | Mouse | Urine | >8-24 | GC-MS | [14] |
| Glucose | *IL10^-/-^* | Mouse | Urine | >8-24 | GC-MS | [14] |
| Glucose δ 4.64 | *IL10^-/-^* | Mouse | Plasma | 0-3 | ^1^H-NMR | [24] |
| Glucose δ 4.64 | *IL10^-/-^* | Mouse | Plasma | >8-24 | ^1^H-NMR | [24] |
| Glucuronoic lactone | DSS (A) | Mouse | Serum | >8-24 | GC-MS | [15] |
| Glutamate | DSS (A) | Mouse | Feces | >3-8 | ^1^H-NMR | [19] |
| Glutamine | Adoptive transfer | Mouse | Stool | >8-24 | ^1^H-NMR | [1] |
| Glutamine | DSS (A) | Mouse | Feces | >3-8 | ^1^H-NMR | [19] |
| Glutamine | DSS (A) | Mouse | Liver | >8-24 | ^1^H-NMR | [13] |
| Glutamine | DSS (A) | Mouse | Serum | >3-8 | ^1^H-NMR | [16] |
| Glutamine δ 2.45 | *IL10^-/-^* | Mouse | Plasma | >8-24 | ^1^H-NMR | [24] |
| Glutamine, L- | DSS (A) | Mouse | Serum | >8-24 | GC-MS | [15] |
| Glutamine, L- | DSS (A) | Mouse | Colon | >8-24 | GC-MS | [15] |
| Glutamine, L- | DSS (A) | Mouse | Serum | >8-24 | GC-MS | [15] |
| Glutamylcysteine, γ- | DSS (A) | Piglet | Colon (distal), red blood cells | 0-3 | UHPLC/MS-MS | [8] |
| Glutamylglutamic acid, γ- | H. hepaticus | Mouse | Serum | >24 | UPLC-ESI-TOF-MS | [12] |
| Glutamyl-L-leucine, L-γ- | H. hepaticus | Mouse | Serum | >8-24 | UPLC-ESI-TOF-MS | [12] |
| Glutaric acid | *IL10^-/-^* | Mouse | Urine | >3-8 | GC-MS | [6] |
| Glutaric acid | *IL10^-/-^* | Mouse | Urine | >3-8 | GC-MS | [6] |
| Glutaric acid | *IL10^-/-^* | Mouse | Urine | >3-8 | GC-MS | [6] |
| Glutaric acid | *IL10^-/-^* | Mouse | Urine | >3-8 | GC-MS | [6] |
| Glutaric acid | *IL10^-/-^* | Mouse | Urine | >8-24 | GC-MS | [6] |
| Glutaric acid | *IL10^-/-^* | Mouse | Urine | >8-24 | GC-MS | [6] |
| Glutaric acid | *IL10^-/-^* | Mouse | Urine | >8-24 | GC-MS | [6] |
| Glutaric acid | *IL10^-/-^* | Mouse | Urine | >8-24 | GC-MS | [6] |
| Glutaric acid | *IL10^-/-^* | Mouse | Urine | >3-8 | GC-MS | [14] |
| Glutaric acid | *IL10^-/-^* | Mouse | Urine | >3-8 | GC-MS | [14] |
| Glutaric acid | *IL10^-/-^* | Mouse | Urine | >3-8 | GC-MS | [14] |
| Glutaric acid | *IL10^-/-^* | Mouse | Urine | >8-24 | GC-MS | [14] |
| Glutaric acid | *IL10^-/-^* | Mouse | Urine | >8-24 | GC-MS | [14] |
| Glutaric acid | *IL10^-/-^* | Mouse | Urine | >8-24 | GC-MS | [14] |
| Glutaric acid | *IL10^-/-^* | Mouse | Urine | >8-24 | GC-MS | [14] |
| Glutathione | DSS (A) | Piglet | Colon (distal) | 0-3 | UHPLC/MS-MS | [8] |
| Glutathione (Total) | DSS (A) | Mouse | Colon | >8-24 | NMR(1H, 1C, 1P) | [20] |
| Glycerophosphocholine | DSS (A) | Mouse | Colon | >8-24 | ^1^H-NMR | [13] |
| Glycine | DSS (A) | Mouse | Serum | >3-8 | ^1^H-NMR | [16] |
| Glycine (2TMS) | *Winnie* | Mouse | Feces | >8-24 | GC-MS | [17] |
| Glycine, L- | DSS (A) | Mouse | Serum | >8-24 | GC-MS | [15] |
| Glycochenodeoxycholic acid | DSS (A) | Mouse | Plasma | >8-24 | UPLC-MS | [23] |
| Glycocholic acid 3- glucuronide | H. hepaticus | Mouse | Serum | >24 | UPLC-ESI-TOF-MS | [12] |
| Glycocholic acid 3-glucuronide | H. hepaticus | Mouse | Serum | >8-24 | UPLC-ESI-TOF-MS | [12] |
| GPL lysoPCa C14:0 | *TNF*^ΔARE/WT^ | Mouse | Ileum (distal) | >8-24 | LC-MS | [5] |
| GPL lysoPCa C14:0 | *TNF*^ΔARE/WT^ | Mouse | Ileum (distal) | >8-24 | LC-MS | [5] |
| GPL lysoPCa C20:3 | *TNF*^ΔARE/WT^ | Mouse | Ileum (distal) | >8-24 | LC-MS | [5] |
| GPL lysoPCa C20:3 | *TNF*^ΔARE/WT^ | Mouse | Ileum (distal) | >8-24 | LC-MS | [5] |
| GPL lysoPCa C26:0 | *TNF*^ΔARE/WT^ | Mouse | Ileum (distal) | >8-24 | LC-MS | [5] |
| GPL lysoPCa C26:1 | *TNF*^ΔARE/WT^ | Mouse | Ileum (distal) | >8-24 | LC-MS | [5] |
| GPL lysoPCa C26:1 | *TNF*^ΔARE/WT^ | Mouse | Ileum (distal) | >8-24 | LC-MS | [5] |
| GPL lysoPCa C6:0 | *TNF*^ΔARE/WT^ | Mouse | Ileum (distal) | >8-24 | LC-MS | [5] |
| GPL PCaa C26:0 | *TNF*^ΔARE/WT^ | Mouse | Ileum (distal) | >8-24 | LC-MS | [5] |
| GPL PCaa C26:0 | *TNF*^ΔARE/WT^ | Mouse | Ileum (distal) | >8-24 | LC-MS | [5] |
| GPL PCaa C36:6 | *TNF*^ΔARE/WT^ | Mouse | Ileum (distal) | >3-8 | LC-MS | [5] |
| GPL PCaa C36:6 | *TNF*^ΔARE/WT^ | Mouse | Ileum (distal) | >8-24 | LC-MS | [5] |
| GPL PCaa C40:1 | *TNF*^ΔARE/WT^ | Mouse | Ileum (distal) | >8-24 | LC-MS | [5] |
| GPL PCaa C40:6 | *TNF*^ΔARE/WT^ | Mouse | Ileum (distal) | >8-24 | LC-MS | [5] |
| GPL PCaa C40:6 | *TNF*^ΔARE/WT^ | Mouse | Ileum (distal) | >8-24 | LC-MS | [5] |
| GPL PCaa C42:5 | *TNF*^ΔARE/WT^ | Mouse | Ileum (distal) | >8-24 | LC-MS | [5] |
| GPL PCaa C42:6 | *TNF*^ΔARE/WT^ | Mouse | Ileum (distal) | >8-24 | LC-MS | [5] |
| GPL PCaa C42:6 | *TNF*^ΔARE/WT^ | Mouse | Ileum (distal) | >8-24 | LC-MS | [5] |
| GPL PCae 42:4 | *TNF*^ΔARE/WT^ | Mouse | Ileum (distal) | >8-24 | LC-MS | [5] |
| GPL PCae C36:0 | *TNF*^ΔARE/WT^ | Mouse | Ileum (distal) | >8-24 | LC-MS | [5] |
| GPL PCae C40:0 | *TNF*^ΔARE/WT^ | Mouse | Ileum (distal) | >8-24 | LC-MS | [5] |
| GPL PCae C40:0 | *TNF*^ΔARE/WT^ | Mouse | Ileum (distal) | >8-24 | LC-MS | [5] |
| GPL PCae C40:4 | *TNF*^ΔARE/WT^ | Mouse | Ileum (distal) | >8-24 | LC-MS | [5] |
| GPL PCae C42:0 | *TNF*^ΔARE/WT^ | Mouse | Ileum (distal) | >8-24 | LC-MS | [5] |
| GPL PCae C42:5 | *TNF*^ΔARE/WT^ | Mouse | Ileum (distal) | >8-24 | LC-MS | [5] |
| GPL PCae C42:5 | *TNF*^ΔARE/WT^ | Mouse | Ileum (distal) | >8-24 | LC-MS | [5] |
| GPL PCae C44:6 | *TNF*^ΔARE/WT^ | Mouse | Ileum (distal) | >8-24 | LC-MS | [5] |
| Guanidinopropionic acid, β- | H. hepaticus | Mouse | Serum | >24 | UPLC-ESI-TOF-MS | [12] |
| Hexahomomethionine | T-syn deficiency | Mouse | Colon (distal), cecum | >3-8 | UPLC/ToF-MS | [10] |
| Hexahomomethionine | T-syn deficiency | Mouse | Colon (distal), cecum | >8-24 | UPLC/ToF-MS | [10] |
| Hexanoylcarnitine | DSS (A) | Mouse | Plasma | >8-24 | UPLC-MS | [23] |
| Hexanoylglycine | *IL10^-/-^* | Mouse | Urine | >8-24 | GC-MS | [6] |
| Hexanoylglycine | *IL10^-/-^* | Mouse | Urine | >8-24 | GC-MS | [6] |
| Hexanoylglycine | *IL10^-/-^* | Mouse | Urine | >3-8 | GC-MS | [14] |
| Hexanoylglycine | *IL10^-/-^* | Mouse | Urine | >3-8 | GC-MS | [14] |
| Hexanoylglycine | *IL10^-/-^* | Mouse | Urine | >8-24 | GC-MS | [14] |
| Hexanoylglycine | *IL10^-/-^* | Mouse | Urine | >8-24 | GC-MS | [14] |
| Hippurate | DSS (A) | Mouse | Urine | >8-24 | ^1^H-NMR | [13] |
| Hippurate | DSS (A) | Mouse | Urine | >8-24 | ^1^H-NMR | [13] |
| Hippurate | DSS (A) | Mouse | Urine | >8-24 | ^1^H-NMR | [13] |
| Hippurate | DSS (A) | Mouse | Urine | >8-24 | ^1^H-NMR | [13] |
| Hippurate | DSS (A) | Mouse | Urine | >8-24 | ^1^H-NMR | [13] |
| Hippuric acid | H. hepaticus | Mouse | Serum | >24 | UPLC-ESI-TOF-MS | [12] |
| Histidine | DSS (A) | Mouse | Serum | >3-8 | ^1^H-NMR | [16] |
| Hypoxanthine | DSS (A) | Mouse | Spleen | >8-24 | ^1^H-NMR | [13] |
| Indol-3-acetic acid | DSS (A) | Mouse | Serum | >8-24 | GC-MS | [15] |
| Indol-3-acetic acid | DSS (A) | Mouse | Colon | >8-24 | GC-MS | [15] |
| Indol-3-acetic acid | DSS (A) | Mouse | Serum | >8-24 | GC-MS | [15] |
| Indoleacrylic acid | H. hepaticus | Mouse | Serum | >8-24 | UPLC-ESI-TOF-MS | [12] |
| Indoleacrylic acid | H. hepaticus | Mouse | Serum | >24 | UPLC-ESI-TOF-MS | [12] |
| Indolelactic acid | H. hepaticus | Mouse | Serum | >24 | UPLC-ESI-TOF-MS | [12] |
| Indoxylsulfate | DSS (A) | Mouse | Urine | >8-24 | ^1^H-NMR | [13] |
| Indoxylsulfate | DSS (A) | Mouse | Urine | >8-24 | ^1^H-NMR | [13] |
| Indoxylsulfate | DSS (A) | Mouse | Urine | >8-24 | ^1^H-NMR | [13] |
| Indoxylsulfate | DSS (A) | Mouse | Urine | >8-24 | ^1^H-NMR | [13] |
| Indoxylsulfate | DSS (A) | Mouse | Urine | >8-24 | ^1^H-NMR | [13] |
| Indoxylsulfate | DSS (A) | Mouse | Urine | >8-24 | ^1^H-NMR | [13] |
| Inosine | T-syn deficiency | Mouse | Colon (distal), cecum | 0-3 | UPLC/ToF-MS | [10] |
| Inositol 1,3,4,5,6-pentakisphosphate, 1D-myo- | T-syn deficiency | Mouse | Colon (distal), cecum | >3-8 | UPLC/ToF-MS | [10] |
| Iridotrial | T-syn deficiency | Mouse | Colon (distal), cecum | >3-8 | UPLC/ToF-MS | [10] |
| Isocitric acid | DSS (A) | Mouse | Serum | >8-24 | GC-MS | [15] |
| Isocitric acid | DSS (A) | Mouse | Serum | >8-24 | GC-MS | [15] |
| Isocitric acid | *IL10^-/-^* | Mouse | Urine | >3-8 | GC-MS | [6] |
| Isocitric acid | *IL10^-/-^* | Mouse | Urine | >3-8 | GC-MS | [6] |
| Isocitric acid | *IL10^-/-^* | Mouse | Urine | >8-24 | GC-MS | [6] |
| Isocitric acid | *IL10^-/-^* | Mouse | Urine | >8-24 | GC-MS | [6] |
| Isocitric acid | *IL10^-/-^* | Mouse | Urine | >8-24 | GC-MS | [6] |
| Isocitric acid | *IL10^-/-^* | Mouse | Urine | >3-8 | GC-MS | [14] |
| Isocitric acid | *IL10^-/-^* | Mouse | Urine | >8-24 | GC-MS | [14] |
| Isoleucine, N-(trimethylsilyl)-, trimethylsilyl ester, L- | *Winnie* | Mouse | Feces | >8-24 | GC-MS | [17] |
| Isovaleroylglycine, N- | *IL10^-/-^* | Mouse | Urine | >8-24 | NMR | [7] |
| Isovaleroylglycine, N- | *IL10^-/-^* | Mouse | Urine | >8-24 | NMR | [7] |
| Ketoglutarate, α- | TNBS | Rat | Urine | ? | UPLC-MS/MS | [21] |
| Ketoglutarate, α- | TNBS | Rat | Urine | ? | UPLC-MS/MS | [21] |
| Ketoglutarate, α- | TNBS | Rat | Urine | ? | UPLC-MS/MS | [21] |
| Ketoglutaric acid, α- | DSS (A) | Mouse | Plasma | >8-24 | UPLC-MS | [23] |
| Kynurenic acid | *IL10^-/-^* | Mouse | Plasma | >8-24 | LC-MS | [6] |
| Kynurenic acid | H. hepaticus | Mouse | Serum | >24 | UPLC-ESI-TOF-MS | [12] |
| Lactate | DSS (A) | Mouse | Serum | >3-8 | ^1^H-NMR | [16] |
| Leucine δ 1.01 | *IL10^-/-^* | Mouse | Plasma | >8-24 | ^1^H-NMR | [24] |
| Linoleic acid | H. hepaticus | Mouse | Serum | >24 | UPLC-ESI-TOF-MS | [12] |
| Linolenic acid, α- | H. hepaticus | Mouse | Serum | >24 | UPLC-ESI-TOF-MS | [12] |
| Lipoproteins mainly VLDL δ 1.56 | *IL10^-/-^* | Mouse | Plasma | 0-3 | ^1^H-NMR | [24] |
| Lipoproteins mainly VLDL δ 1.56 | *IL10^-/-^* | Mouse | Plasma | >3-8 | ^1^H-NMR | [24] |
| Lipoproteins mainly VLDL δ 1.56 | *IL10^-/-^* | Mouse | Plasma | >8-24 | ^1^H-NMR | [24] |
| Lipoproteins δ 0.88 | *IL10^-/-^* | Mouse | Plasma | 0-3 | ^1^H-NMR | [24] |
| Lipoproteins δ 0.88 | *IL10^-/-^* | Mouse | Plasma | >3-8 | ^1^H-NMR | [24] |
| Lipoproteins δ 1.25-1.27 | *IL10^-/-^* | Mouse | Plasma | >3-8 | ^1^H-NMR | [24] |
| Lipoproteins δ 1.27-1.33 | *IL10^-/-^* | Mouse | Plasma | 0-3 | ^1^H-NMR | [24] |
| Lipoproteins δ 1.27-1.33 | *IL10^-/-^* | Mouse | Plasma | >3-8 | ^1^H-NMR | [24] |
| Lysine | DSS (A) | Mouse | Feces | >3-8 | ^1^H-NMR | [19] |
| LysoPC (14:0) | H. hepaticus | Mouse | Serum | >24 | UPLC-ESI-TOF-MS | [12] |
| LysoPC (15:0) | H. hepaticus | Mouse | Serum | >24 | UPLC-ESI-TOF-MS | [12] |
| LysoPC (16:1) | H. hepaticus | Mouse | Serum | >8-24 | UPLC-ESI-TOF-MS | [12] |
| LysoPC (16:1) | H. hepaticus | Mouse | Serum | >24 | UPLC-ESI-TOF-MS | [12] |
| LysoPC (17:0) | H. hepaticus | Mouse | Serum | >8-24 | UPLC-ESI-TOF-MS | [12] |
| LysoPC (18:1) | H. hepaticus | Mouse | Serum | >8-24 | UPLC-ESI-TOF-MS | [12] |
| LysoPC (18:2) | H. hepaticus | Mouse | Serum | >24 | UPLC-ESI-TOF-MS | [12] |
| LysoPC (18:3) | H. hepaticus | Mouse | Serum | >24 | UPLC-ESI-TOF-MS | [12] |
| LysoPC (18:4) | H. hepaticus | Mouse | Serum | >8-24 | UPLC-ESI-TOF-MS | [12] |
| LysoPC (18:4) | H. hepaticus | Mouse | Serum | >24 | UPLC-ESI-TOF-MS | [12] |
| LysoPC (20:3) | H. hepaticus | Mouse | Serum | >24 | UPLC-ESI-TOF-MS | [12] |
| LysoPC (20:4(5Z,8Z,11Z,14Z)) | DSS (C) | Mouse | Liver | >8-24 | LC-qTOF-MS | [18] |
| LysoPC (20:4(5Z,8Z,11Z,14Z)) | DSS (C) | Mouse | Liver | >8-24 | LC-qTOF-MS | [18] |
| LysoPC (20:4) | H. hepaticus | Mouse | Serum | >24 | UPLC-ESI-TOF-MS | [12] |
| LysoPC (20:5) | H. hepaticus | Mouse | Serum | >8-24 | UPLC-ESI-TOF-MS | [12] |
| LysoPC (20:5) | H. hepaticus | Mouse | Serum | >24 | UPLC-ESI-TOF-MS | [12] |
| LysoPC (22:6) | H. hepaticus | Mouse | Serum | >24 | UPLC-ESI-TOF-MS | [12] |
| LysoPC (O-18:0) | DSS (C) | Mouse | Liver | >8-24 | LC-qTOF-MS | [18] |
| LysoPC18:1 | DSS (A) | Mouse | Serum | >8-24 | UPLC-MS | [26] |
| LysoPC18:1 | DSS (A) | Mouse | Colon | >8-24 | UPLC-MS | [26] |
| LysoPC18:2 | DSS (A) | Mouse | Serum | >8-24 | UPLC-MS | [26] |
| LysoPC18:2 | DSS (A) | Mouse | Colon | >8-24 | UPLC-MS | [26] |
| LysoPE (0:0/22:2(13Z,16Z)) | DSS (C) | Mouse | Liver | >8-24 | LC-qTOF-MS | [18] |
| LysoPE (18:1) | H. hepaticus | Mouse | Serum | >8-24 | UPLC-ESI-TOF-MS | [12] |
| LysoPE (18:1) | H. hepaticus | Mouse | Serum | >24 | UPLC-ESI-TOF-MS | [12] |
| LysoPE (18:2) | H. hepaticus | Mouse | Serum | >24 | UPLC-ESI-TOF-MS | [12] |
| LysoPE (20:1) | H. hepaticus | Mouse | Serum | >24 | UPLC-ESI-TOF-MS | [12] |
| LysoPE (22:5) | H. hepaticus | Mouse | Serum | >8-24 | UPLC-ESI-TOF-MS | [12] |
| LysoPE (22:5) | H. hepaticus | Mouse | Serum | >24 | UPLC-ESI-TOF-MS | [12] |
| Malic acid | DSS (A) | Mouse | Serum | >8-24 | GC-MS | [15] |
| Maltotriose | DSS (C) | Mouse | Liver | >8-24 | LC-qTOF-MS | [18] |
| Methionine | DSS (A) | Mouse | Serum | >3-8 | ^1^H-NMR | [16] |
| Methionine δ 2.14 | *IL10^-/-^* | Mouse | Plasma | >8-24 | ^1^H-NMR | [24] |
| Methyl in FA | *TNF*^ΔARE/WT^ | Mouse | Colon (proximal) | >8-24 | ^1^H-NMR | [5] |
| Methyl in FA | *TNF*^ΔARE/WT^ | Mouse | Ileum (distal) | >8-24 | ^1^H-NMR | [5] |
| Methylamine | DSS (A) | Mouse | Urine | >8-24 | ^1^H-NMR | [13] |
| Methylamine | DSS (A) | Mouse | Urine | >8-24 | ^1^H-NMR | [13] |
| Methylamine | DSS (A) | Mouse | Urine | >8-24 | ^1^H-NMR | [13] |
| Methylamine | DSS (A) | Mouse | Urine | >8-24 | ^1^H-NMR | [13] |
| Methylamine | DSS (A) | Mouse | Urine | >8-24 | ^1^H-NMR | [13] |
| Methylamine | DSS (A) | Mouse | Urine | >8-24 | ^1^H-NMR | [13] |
| Methylguanidine | DSS (A) | Mouse | Urine | >8-24 | ^1^H-NMR | [13] |
| Methylguanidine | DSS (A) | Mouse | Urine | >8-24 | ^1^H-NMR | [13] |
| Methylguanidine | DSS (A) | Mouse | Urine | >8-24 | ^1^H-NMR | [13] |
| Methylguanidine | DSS (A) | Mouse | Urine | >8-24 | ^1^H-NMR | [13] |
| Methylimidazoleacetic acid | H. hepaticus | Mouse | Serum | >8-24 | UPLC-ESI-TOF-MS | [12] |
| Methylnicotinate, N- | DSS (A) | Mouse | Urine | >8-24 | ^1^H-NMR | [13] |
| Methylnicotinate, N- | DSS (A) | Mouse | Urine | >8-24 | ^1^H-NMR | [13] |
| Methylnicotinate, N- | DSS (A) | Mouse | Urine | >8-24 | ^1^H-NMR | [13] |
| Myoinositol | DSS (A) | Mouse | Colon | >8-24 | GC-MS | [25] |
| Norleucine, N-(trimethylsilyl)-, trimethylsilyl ester, L- | *Winnie* | Mouse | Feces | >8-24 | GC-MS | [17] |
| Octadecanedioic acid | H. hepaticus | Mouse | Serum | >8-24 | UPLC-ESI-TOF-MS | [12] |
| Oleic acid C18:1 | DSS (A) | Mouse | Serum | >8-24 | GC-MS | [26] |
| Opht A | DSS (A) | Mouse | Plasma | >8-24 | UPLC-MS | [23] |
| Pantothenic acid | *IL10^-/-^* | Mouse | Urine | >3-8 | GC-MS | [14] |
| Pantothenic acid | *IL10^-/-^* | Mouse | Urine | >8-24 | GC-MS | [14] |
| Phenylacetylglycine | DSS (A) | Mouse | Urine | >8-24 | ^1^H-NMR | [13] |
| Phenylacetylglycine | DSS (A) | Mouse | Urine | >8-24 | ^1^H-NMR | [13] |
| Phenylacetylglycine | DSS (A) | Mouse | Urine | >8-24 | ^1^H-NMR | [13] |
| Phenylalanine 1 TM, 3-phenyl-, trimethylsilyl ester, DL- | *Winnie* | Mouse | Feces | >8-24 | GC-MS | [17] |
| Phenyllactic acid | H. hepaticus | Mouse | Serum | >8-24 | UPLC-ESI-TOF-MS | [12] |
| Phosphatidic acid (18:0/22:0)[U] | DSS (C) | Mouse | Liver | >8-24 | LC-qTOF-MS | [18] |
| Phosphatidic acid (18:0/22:2(13Z,16Z)) | DSS (C) | Mouse | Liver | >8-24 | LC-qTOF-MS | [18] |
| PC (14:0/20:0) | DSS (C) | Mouse | Liver | >8-24 | LC-qTOF-MS | [18] |
| PC (16:0/18:1(11E)) | DSS (C) | Mouse | Liver | >8-24 | LC-qTOF-MS | [18] |
| PC (18:3(9Z,12Z,15Z)/16:0) | DSS (C) | Mouse | Liver | >8-24 | LC-qTOF-MS | [18] |
| PG (12:0/15:0) | DSS (C) | Mouse | Spleen | >8-24 | LC-qTOF-MS | [18] |
| PS (18:0/18:4(6Z,9Z,12Z,15Z)) | DSS (C) | Mouse | Spleen | >8-24 | LC-qTOF-MS | [18] |
| PS (18:2(9Z,12Z)/12:0) | DSS (C) | Mouse | Spleen | >8-24 | LC-qTOF-MS | [18] |
| Phosphocholine | DSS (A) | Mouse | Colon | >8-24 | ^1^H-NMR | [13] |
| Phytyl diphosphate | T-syn deficiency | Mouse | Colon (distal), cecum | >3-8 | UPLC/ToF-MS | [10] |
| Phytyl diphosphate | T-syn deficiency | Mouse | Colon (distal), cecum | >8-24 | UPLC/ToF-MS | [10] |
| Pimelate | T-syn deficiency | Mouse | Colon (distal), cecum | >3-8 | UPLC/ToF-MS | [10] |
| Pipecolate | Adoptive transfer | Mouse | Urine | >8-24 | ^1^H-NMR | [1] |
| Pipecolic acid | H. hepaticus | Mouse | Serum | >8-24 | UPLC-ESI-TOF-MS | [12] |
| Polyunsaturated fatty acid (δ=2.74-2.79) (pC) | *TNF*^ΔARE/WT^ | Mouse | Colon (proximal) | >8-24 | ^1^H-NMR | [5] |
| Proline | Adoptive transfer | Mouse | Urine | >8-24 | ^1^H-NMR | [1] |
| Proline | DSS (A) | Mouse | Serum | >3-8 | ^1^H-NMR | [16] |
| Protoporphyrinogen IX | T-syn deficiency | Mouse | Colon (distal), cecum | >3-8 | UPLC/ToF-MS | [10] |
| Purine | DSS (A) | Mouse | Colon | >8-24 | GC-MS | [25] |
| Putrescine 4TMS | *Winnie* | Mouse | Feces | >8-24 | GC-MS | [17] |
| Quinolinic acid | *IL10^-/-^* | Mouse | Plasma | >8-24 | LC-MS | [6] |
| Retinyl-conjugates | Adoptive transfer | Mouse | Liver | >8-24 | ^1^H-NMR | [1] |
| Sebacic acid, bis(trimethylsilyl) ester | *Winnie* | Mouse | Feces | >8-24 | GC-MS | [17] |
| Sphingomyelin (d18:1/20:0) | H. hepaticus | Mouse | Serum | >24 | UPLC-ESI-TOF-MS | [12] |
| Sphingomyelin (OH) C14:1 | *TNF*^ΔARE/WT^ | Mouse | Ileum (distal) | >8-24 | LC-MS | [5] |
| Sphingomyelin C16:1 | *TNF*^ΔARE/WT^ | Mouse | Ileum (distal) | >3-8 | LC-MS | [5] |
| Succinate | Adoptive transfer | Mouse | Urine | >8-24 | ^1^H-NMR | [1] |
| Succinate | *IL10^-/-^* | Mouse | Urine | >8-24 | NMR | [7] |
| Succinic acid | DSS (A) | Mouse | Serum | >8-24 | GC-MS | [15] |
| Sucrose | T-syn deficiency | Mouse | Colon (distal), cecum | >8-24 | UPLC/ToF-MS | [10] |
| Tartrate | DSS (A) | Mouse | Serum | >3-8 | ^1^H-NMR | [16] |
| Taurine | DSS (A) | Mouse | Colon | >8-24 | ^1^H-NMR | [13] |
| Taurine | DSS (A) | Mouse | Spleen | >8-24 | ^1^H-NMR | [13] |
| Threonine | DSS (A) | Mouse | Feces | >3-8 | ^1^H-NMR | [19] |
| Tiglylglycine | DSS (A) | Mouse | Serum | >8-24 | GC-MS | [15] |
| Traumatic acid | H. hepaticus | Mouse | Serum | >24 | UPLC-ESI-TOF-MS | [12] |
| Triacylglycerides | Adoptive transfer | Mouse | Liver | >8-24 | ^1^H-NMR | [1] |
| Triglycerides | *TNF*^ΔARE/WT^ | Mouse | Colon (proximal) | >8-24 | ^1^H-NMR | [5] |
| Triglycerides | *TNF*^ΔARE/WT^ | Mouse | Ileum (distal) | >8-24 | ^1^H-NMR | [5] |
| Trimethylamine δ 2.87 | *IL10^-/-^* | Mouse | Plasma | >8-24 | ^1^H-NMR | [24] |
| Trimethylamine δ 2.87 | *IL10^-/-^* | Mouse | Plasma | >8-24 | ^1^H-NMR | [24] |
| Tryptophan | *IL10^-/-^* | Mouse | Plasma | >8-24 | LC-MS | [6] |
| Tryptophan | *IL10^-/-^* | Mouse | Plasma | >8-24 | LC-MS | [6] |
| Tryptophan, L- | DSS (A) | Mouse | Serum | >8-24 | GC-MS | [15] |
| Tryptophan, L- | DSS (A) | Mouse | Serum | >8-24 | GC-MS | [15] |
| Tryptophan, L- | DSS (A) | Mouse | Plasma | >8-24 | UPLC-MS | [23] |
| Tyrosine | DSS (A) | Mouse | Serum | >3-8 | ^1^H-NMR | [16] |
| Tyrosine δ 6.9 | *IL10^-/-^* | Mouse | Plasma | >8-24 | ^1^H-NMR | [24] |
| Tyrosine, DL- (3TMS) | *Winnie* | Mouse | Feces | >8-24 | GC-MS | [17] |
| Tyrosine, L- | DSS (A) | Mouse | Serum | >8-24 | GC-MS | [15] |
| Tyrosine, L- | DSS (A) | Mouse | Serum | >8-24 | GC-MS | [15] |
| Tyrosine, o- | DSS (A) | Mouse | Plasma | >8-24 | UPLC-MS | [23] |
| Unidentified | TNBS | Rat | Plasma | ? | UPLC-ESI-qTOF-MS | [11] |
| Unknown at RT 193 | *IL10^-/-^* | Mouse | Urine | >3-8 | GC-MS | [14] |
| Unknown at RT 1933 | *IL10^-/-^* | Mouse | Urine | >3-8 | GC-MS | [14] |
| Unknown at RT 1933 | *IL10^-/-^* | Mouse | Urine | >8-24 | GC-MS | [14] |
| Unknown at RT 1933 | *IL10^-/-^* | Mouse | Urine | >8-24 | GC-MS | [14] |
| Unknown at RT 1933 | *IL10^-/-^* | Mouse | Urine | >8-24 | GC-MS | [14] |
| Unknown at RT 1933 | *IL10^-/-^* | Mouse | Urine | >8-24 | GC-MS | [14] |
| Unknown at RT 2800 | *IL10^-/-^* | Mouse | Urine | >3-8 | GC-MS | [14] |
| Unknown at RT1933 | *IL10^-/-^* | Mouse | Urine | >8-24 | GC-MS | [14] |
| Unknown at RT2800 | *IL10^-/-^* | Mouse | Urine | >8-24 | GC-MS | [14] |
| Unknown m/z 308- | *IL10^-/-^* | Mouse | Urine | >8-24 | LC-MS | [22] |
| Unknown m/z 495-/497+ | *IL10^-/-^* | Mouse | Urine | >8-24 | LC-MS | [22] |
| Unknown RT1177n | *IL10^-/-^* | Mouse | Urine | >8-24 | GC-MS | [6] |
| Unknown RT1177n | *IL10^-/-^* | Mouse | Urine | >8-24 | GC-MS | [6] |
| Unknown RT1451n | *IL10^-/-^* | Mouse | Urine | >3-8 | GC-MS | [6] |
| Unknown RT1891n | *IL10^-/-^* | Mouse | Urine | >3-8 | GC-MS | [6] |
| Unknown RT1891n | *IL10^-/-^* | Mouse | Urine | >3-8 | GC-MS | [6] |
| Unknown RT1891n | *IL10^-/-^* | Mouse | Urine | >3-8 | GC-MS | [6] |
| Unknown RT1891n | *IL10^-/-^* | Mouse | Urine | >8-24 | GC-MS | [6] |
| Unknown RT1891n | *IL10^-/-^* | Mouse | Urine | >8-24 | GC-MS | [6] |
| Unknown RT1933 | *IL10^-/-^* | Mouse | Urine | >3-8 | GC-MS | [6] |
| Unknown RT1933 | *IL10^-/-^* | Mouse | Urine | >8-24 | GC-MS | [6] |
| Unknown RT1933 | *IL10^-/-^* | Mouse | Urine | >8-24 | GC-MS | [6] |
| Unknown RT1933 | *IL10^-/-^* | Mouse | Urine | >8-24 | GC-MS | [6] |
| Unknown RT1933 | *IL10^-/-^* | Mouse | Urine | >8-24 | GC-MS | [6] |
| Unknown RT2081n | *IL10^-/-^* | Mouse | Urine | >8-24 | GC-MS | [6] |
| Unknown RT2800 | *IL10^-/-^* | Mouse | Urine | >3-8 | GC-MS | [6] |
| Unknown RT2800 | *IL10^-/-^* | Mouse | Urine | >8-24 | GC-MS | [6] |
| Unknown RT2800 | *IL10^-/-^* | Mouse | Urine | >8-24 | GC-MS | [6] |
| Unknown RT910n | *IL10^-/-^* | Mouse | Urine | >8-24 | GC-MS | [6] |
| Unknown RT954n | *IL10^-/-^* | Mouse | Urine | >8-24 | GC-MS | [6] |
| Unknown RT954n | *IL10^-/-^* | Mouse | Urine | >8-24 | GC-MS | [6] |
| Unsaturated fatty acids | Adoptive transfer | Mouse | Liver | >8-24 | ^1^H-NMR | [1] |
| Unsaturated fatty acids | *TNF*^ΔARE/WT^ | Mouse | Colon (proximal) | >8-24 | ^1^H-NMR | [5] |
| Unsaturated fatty acids | *TNF*^ΔARE/WT^ | Mouse | Ileum (distal) | >8-24 | ^1^H-NMR | [5] |
| Uracil | DSS (A) | Mouse | Feces | >3-8 | ^1^H-NMR | [19] |
| Uracil | DSS (A) | Mouse | Colon | >8-24 | ^1^H-NMR | [13] |
| Uracil | *IL10^-/-^* | Mouse | Urine | >3-8 | GC-MS | [6] |
| Uridine | DSS (A) | Mouse | Colon | >8-24 | ^1^H-NMR | [13] |
| Urocanic acid | H. hepaticus | Mouse | Serum | >8-24 | UPLC-ESI-TOF-MS | [12] |
| Xanthosine | DSS (A) | Mouse | Plasma | >8-24 | UPLC-MS | [23] |
| Xanthurenic acid glucuronide | *IL10^-/-^* | Mouse | Urine | >8-24 | LC-MS | [22] |

‘-‘ indicates that no metabolites were found to be significantly increased in the respective sample.

Model: (A): acute; ARE: AU-rich elements; (C): chronic; DSS: dextran sodium sulfate; H. hepaticus: Helicobacter hepaticus; IL: interleukin; T-syn: T-synthase; TNBS: 2,4,6-trinitrobenzenesulfonic acid; TNF: tumor necrosis factor; WT: wild-type. Platform: GC-MS: gas chromatography-mass spectrometry; HPLC-MS/MS: high performance liquid chromatography tandem mass spectrometry; LC-MS: liquid chromatography-mass spectrometry; LC-qTOF-MS: liquid chromatography quadropole time-of-flight mass spectrometry; MRS: magnetic resonance spectroscopy; NMR: nuclear magnetic resonance; UPLC-ESI-(q)TOF-MS: ultra performance liquid chromatography electrospray ionization (quadropole) time-of-flight mass spectrometry; UPLC-ESI (q)ToFMS: ultra performance liquid chromatography electrospray ionization (quadrupole) time-of-flight mass spectrometry; UPLC-MS: ultra performance liquid chromatography mass spectrometry; UPLC-MS/MS: ultra performance liquid chromatography tandem mass spectrometry; UPLC/ToFMS: ultra performance liquid chromatography time-of-flight mass spectrometry. GPL PCae: cholin glycerophospholipid with an ether bond; GPL PCaa: cholin glycerophospholipid; GPL LysoPCa/LysoPC: lysophosphatidylcholine; LysoPE: lysophosphatidylethanolamine; PC: phosphatidylcholine; PE: phosphatidylethanolamine; PG: phosphatidylglycerol; PS: phosphatidylserine.

**References**

1. Martin, F.P.J.; Lichti, P.; Bosco, N.; Brahmbhatt, V.; Oliveira, M.; Haller, D.; Benyacoub, J. Metabolic phenotyping of an adoptive transfer mouse model of experimental colitis and impact of dietary fish oil intake. *Journal of proteome research* **2015**, *14*, 1911-1919.

2. Kohnke, T.; Gomolka, B.; Bilal, S.; Zhou, X.; Sun, Y.; Rothe, M.; Baumgart, D.C.; Weylandt, K.H. Acetylsalicylic Acid reduces the severity of dextran sodium sulfate-induced colitis and increases the formation of anti-inflammatory lipid mediators. *BioMed research international* **2013**, *2013*, 748160, doi:10.1155/2013/748160.

3. Zhang, W.; Liao, J.; Li, H.; Dong, H.; Bai, H.; Yang, A.; Hammock, B.D.; Yang, G.Y. Reduction of inflammatory bowel disease-induced tumor development in IL-10 knockout mice with soluble epoxide hydrolase gene deficiency. *Molecular Carcinogenesis* **2013**, *52*, 726-738.

4. Willenberg, I.; Ostermann, A.I.; Giovannini, S.; Kershaw, O.; Von Keutz, A.; Steinberg, P.; Schebb, N.H. Effect of acute and chronic DSS induced colitis on plasma eicosanoid and oxylipin levels in the rat. *Prostaglandins and Other Lipid Mediators* **2015**, *120*, 155-160.

5. Baur, P.; Martin, F.P.; Gruber, L.; Bosco, N.; Brahmbhatt, V.; Collino, S.; Guy, P.; Montoliu, I.; Rozman, J.; Klingenspor, M., et al. Metabolic phenotyping of the Crohn's disease-like IBD etiopathology in the TNFDELTAARE/WT mouse model. *Journal of proteome research* **2011**, *10*, 5523-5535.

6. Lin, H.M.; Barnett, M.P.G.; Roy, N.C.; Joyce, N.I.; Zhu, S.; Armstrong, K.; Helsby, N.A.; Ferguson, L.R.; Rowan, D.D. Metabolomic analysis identifies inflammatory and noninflammatory metabolic effects of genetic modification in a mouse model of Crohn?s disease. *Journal of proteome research* **2010**, *9*, 1965-1975.

7. Murdoch, T.B.; Fu, H.; MacFarlane, S.; Sydora, B.C.; Fedorak, R.N.; Slupsky, C.M. Urinary metabolic profiles of inflammatory bowel disease in interleukin-10 gene-deficient mice. *Analytical Chemistry* **2008**, *80*, 5524-5531.

8. Vassilyadi, P.; Harding, S.V.; Nitschmann, E.; Wykes, L.J. Experimental colitis and malnutrition differentially affect the metabolism of glutathione and related sulfhydryl metabolites in different tissues. *European Journal of Nutrition* **2016**, *55*, 1769-1776.

9. Qi, Y.; Jiang, C.; Tanaka, N.; Krausz, K.W.; Brocker, C.N.; Fang, Z.Z.; Bredell, B.X.; Shah, Y.M.; Gonzalez, F.J. PPARalpha-dependent exacerbation of experimental colitis by the hypolipidemic drug fenofibrate. *American Journal of Physiology - Gastrointestinal and Liver Physiology* **2014**, *307*, G564-G573.

10. Jacobs, J.P.; Lin, L.; Goudarzi, M.; Ruegger, P.; McGovern, D.P.B.; Fornace, A.J.; Borneman, J.; Xia, L.; Braun, J. Microbial, metabolomic, and immunologic dynamics in a relapsing genetic mouse model of colitis induced by T-synthase deficiency. *Gut microbes* **2017**, *8*, 1-16.

11. Zhang, X.; Choi, F.F.; Zhou, Y.; Leung, F.P.; Tan, S.; Lin, S.; Xu, H.; Jia, W.; Sung, J.J.; Cai, Z., et al. Metabolite profiling of plasma and urine from rats with TNBS-induced acute colitis using UPLC-ESI-QTOF-MS-based metabonomics--a pilot study. *The FEBS journal* **2012**, *279*, 2322-2338, doi:10.1111/j.1742-4658.2012.08612.x.

12. Lu, K.; Knutson, C.G.; Wishnok, J.S.; Fox, J.G.; Tannenbaum, S.R. Serum metabolomics in a helicobacter hepaticus mouse model of inflammatory bowel disease reveal important changes in the microbiome, serum peptides, and intermediary metabolism. *Journal of proteome research* **2012**, *11*, 4916-4926.

13. Dong, F.; Zhang, L.; Hao, F.; Tang, H.; Wang, Y. Systemic responses of mice to dextran sulfate sodium-induced acute ulcerative colitis using 1H NMR spectroscopy. *Journal of proteome research* **2013**, *12*, 2958-2966.

14. Lin, H.M.; Edmunds, S.J.; Helsby, N.A.; Ferguson, L.R.; Rowan, D.D. Nontargeted urinary metabolite profiling of a mouse model of crohn's disease. *Journal of proteome research* **2009**, *8*, 2045-2057.

15. Shiomi, Y.; Nishiumi, S.; Ooi, M.; Hatano, N.; Shinohara, M.; Yoshie, T.; Kondo, Y.; Furumatsu, K.; Shiomi, H.; Kutsumi, H., et al. GCMS-based metabolomic study in mice with colitis induced by dextran sulfate sodium. *Inflammatory bowel diseases* **2011**, *17*, 2261-2274.

16. Schicho, R.; Nazyrova, A.; Shaykhutdinov, R.; Duggan, G.; Vogel, H.J.; Storr, M. Quantitative metabolomic profiling of serum and urine in DSS-induced ulcerative colitis of mice by 1H NMR spectroscopy. *Journal of proteome research* **2010**, *9*, 6265-6273.

17. Robinson, A.M.; Gondalia, S.V.; Karpe, A.V.; Eri, R.; Beale, D.J.; Morrison, P.D.; Palombo, E.A.; Nurgali, K. Fecal microbiota and metabolome in a mouse model of spontaneous chronic colitis: Relevance to human inflammatory bowel disease. *Inflammatory bowel diseases* **2016**, *22*, 2767-2787.

18. Liu, J.; Xiao, H.T.; Wang, H.S.; Mu, H.X.; Zhao, L.; Du, J.; Yang, D.; Wang, D.; Bian, Z.X.; Lin, S.H. Halofuginone reduces the inflammatory responses of DSS-induced colitis through metabolic reprogramming. *Mol Biosyst* **2016**, *12*, 2296-2303, doi:10.1039/c6mb00154h.

19. Hong, Y.S.; Ahn, Y.T.; Park, J.C.; Lee, J.H.; Lee, H.; Huh, C.S.; Kim, D.H.; Ryu, D.H.; Hwang, G.S. 1H NMR-based metabonomic assessment of probiotic effects in a colitis mouse model. *Archives of pharmacal research* **2010**, *33*, 1091-1101.

20. Kominsky, D.J.; Keely, S.; MacManus, C.F.; Glover, L.E.; Scully, M.; Collins, C.B.; Bowers, B.E.; Campbell, E.L.; Colgan, S.P. An endogenously anti-inflammatory role for methylation in mucosal inflammation identified through metabolite profiling. *J Immunol* **2011**, *186*, 6505-6514, doi:10.4049/jimmunol.1002805.

21. Hou, W.; Zhong, D.; Zhang, P.; Li, Y.; Lin, M.; Liu, G.; Yao, M.; Liao, Q.; Xie, Z. A strategy for the targeted metabolomics analysis of 11 gut microbiota-host co-metabolites in rat serum, urine and feces by ultra high performance liquid chromatography-tandem mass spectrometry. *Journal of Chromatography A* **2016**, *1429*, 207-217.

22. Otter, D.; Cao, M.; Lin, H.M.; Fraser, K.; Edmunds, S.; Lane, G.; Rowan, D. Identification of urinary biomarkers of colon inflammation in IL10-/- mice using Short-Column LCMS metabolomics. *Journal of biomedicine & biotechnology* **2011**, *2011*, 974701, doi:10.1155/2011/974701.

23. Qu, C.; Yuan, Z.W.; Yu, X.T.; Huang, Y.F.; Yang, G.H.; Chen, J.N.; Lai, X.P.; Su, Z.R.; Zeng, H.F.; Xie, Y., et al. Patchouli alcohol ameliorates dextran sodium sulfate-induced experimental colitis and suppresses tryptophan catabolism. *Pharmacological research* **2017**, *121*, 70-82.

24. Martin, F.P.J.; Rezzi, S.; Montoliu, I.; Philippe, D.; Tornier, L.; Messlik, A.; Holzlwimmer, G.; Baur, P.; Quintanilla-Fend, L.; Loh, G., et al. Metabolic assessment of gradual development of moderate experimental colitis in IL-10 deficient mice. *Journal of proteome research* **2009**, *8*, 2376-2387.

25. Gu, X.; Song, Y.; Chai, Y.; Lu, F.; Gonzalez, F.J.; Fan, G.; Qi, Y. GC-MS metabolomics on PPARalpha-dependent exacerbation of colitis. *Molecular bioSystems* **2015**, *11*, 1329-1337.

26. Wang, R.; Gu, X.; Dai, W.; Ye, J.; Lu, F.; Chai, Y.; Fan, G.; Gonzalez, F.J.; Duan, G.; Qi, Y. A lipidomics investigation into the intervention of celastrol in experimental colitis. *Mol Biosyst* **2016**, *12*, 1436-1444, doi:10.1039/c5mb00864f.
